# Cost-effectiveness of 4CMenB Vaccination Against Gonorrhea: Importance of Dosing Schedule, Vaccine Sentiment, Targeting Strategy, and Duration of Protection

**DOI:** 10.1093/infdis/jiae123

**Published:** 2024-04-17

**Authors:** Dariya Nikitin, Lilith K Whittles, Jeffrey W Imai-Eaton, Peter J White

**Affiliations:** Medical Research Council Centre for Global Infectious Disease Analysis and National Institute for Health and Care Research Health Protection Research Unit in Modelling and Health Economics, Imperial College London, London, United Kingdom; Medical Research Council Centre for Global Infectious Disease Analysis and National Institute for Health and Care Research Health Protection Research Unit in Modelling and Health Economics, Imperial College London, London, United Kingdom; Medical Research Council Centre for Global Infectious Disease Analysis and National Institute for Health and Care Research Health Protection Research Unit in Modelling and Health Economics, Imperial College London, London, United Kingdom; Center for Communicable Disease Dynamics, Department of Epidemiology, Harvard T. H. Chan School of Public Health, Boston, Massachusetts, United States of America; Medical Research Council Centre for Global Infectious Disease Analysis and National Institute for Health and Care Research Health Protection Research Unit in Modelling and Health Economics, Imperial College London, London, United Kingdom; Modelling and Economics Unit, UK Health Security Agency, London, United Kingdom

**Keywords:** gonorrhea, cost-effectiveness, transmission-dynamic modeling, vaccine targeting, vaccine sentiment

## Abstract

**Background:**

Observational evidence suggests the 4CMenB meningococcal vaccine may partially protect against gonorrhea, with 1 dose being two-thirds as protective as 2 doses. We examined the cost-effectiveness of vaccinating men who have sex with men (MSM) in England, with 1- or 2-dose primary vaccination.

**Methods:**

Integrated transmission-dynamic health-economic modeling explored the effects of targeting strategy, first- and second-dose uptake levels, and duration of vaccine protection, using observational estimates of vaccine protection.

**Results:**

Vaccination with 1 or 2 primary doses is always cost-saving, irrespective of uptake, although vaccine sentiment is an important determinant of impact and cost-effectiveness. The most impactful and cost-effective targeting is offering “vaccination according to risk” (VaR), to all patients with gonorrhea plus those reporting high numbers of sexual partners. If VaR is not feasible to implement then the more restrictive strategy of “vaccination on diagnosis” (VoD) with gonorrhea is cost-effective, but much less impactful. Under conservative assumptions, VaR (2-dose) saves £7.62M (95% credible interval [CrI], 1.15–17.52) and gains 81.41 (95% CrI, 28.67–164.23) quality-adjusted life-years (QALYs) over 10 years; VoD (2-dose) saves £3.40M (95% CrI, .48–7.71) and gains 41.26 (95% CrI, 17.52–78.25) QALYs versus no vaccination. Optimistic versus pessimistic vaccine-sentiment assumptions increase net benefits by approximately 30% (VoD) or approximately 60% (VaR).

**Conclusions:**

At UK costs, targeted 4CMenB vaccination of MSM gains QALYs and is cost-saving at any uptake level. Promoting uptake maximizes benefits and is an important role for behavioral science.

## Teaser

Targeted vaccination of higher-risk MSM in England is cost-saving regardless of uptake level, even under conservative assumptions about population vaccine sentiment and duration of vaccine protection. Two-dose primary vaccination saves more money and gains more QALYs than 1-dose vaccination.

**(See the Editorial Commentary by Cohen and Marrazzo on pages 37–9.)**

*Neisseria gonorrhoeae* is a World Health Organization “high priority” pathogen for new antibiotic development [1], with increasing antimicrobial resistance limiting treatment options [2, 3]. This concern is increased by high diagnosis rates internationally [4]. Multiple observational studies suggest that the 4CMenB meningitis B vaccine protects against gonorrhea [5–12], with controlled trials underway [12–14]. Despite the current lack of trial evidence, the United Kingdom (UK) Joint Committee on Vaccination and Immunisation (JCVI) advised using 4CMenB to protect MSM against gonorrhea [15, 16], underpinned by our health-economic modeling that used hypothetical effectiveness estimates, and assumed 2-dose primary vaccination is required for any protection [17]. Now there are observational estimates that a single dose provides 26% (95% confidence interval [CI], 12%–37%) protection, two-thirds that of 2 doses (40% [95% CI, 22%–53%]) [5], raising the possibility that single-dose primary vaccination—which would reduce costs and simplify delivery—could be cost-effective.

Vaccination program impact depends on coverage of the relevant population group(s), and understanding vaccine sentiment regarding gonorrhea has been highlighted as important [18]. Men who have sex with men (MSM) are a highly affected group for gonorrhea internationally [2, 3, 13, 18, 19], and evidence to date indicates that gonorrhea vaccination is highly acceptable to MSM [20]. However, uptake of MSM-specific vaccination is often below expectations [21]; for example, recent human papillomavirus (HPV) vaccine uptake by MSM attending sexual health clinics in England [22] has been much lower than anticipated [23]. Vaccination uptake may be determined by a variety of factors ranging from the practical to the principled: some individuals may be uninterested or unwilling to be vaccinated, while others may be willing to be vaccinated but lack motivation at the time of offer [21, 24, 25]. Therefore, it is important to understand how the impact and cost-effectiveness of gonorrhea vaccination are affected by coverage, and how variation in vaccine sentiment in the population affects the build-up of coverage over time, to inform decisions about appropriate budgets for promotional campaigns.

We present the first analysis of 4CMenB vaccination against gonorrhea to investigate how impact and cost-effectiveness are affected by patterns of population vaccine sentiment. We examine 2 alternative realistic approaches to targeting MSM in sexual health clinics according to risk profile. We explore the importance of uptake of the first primary dose and, if offered, the second primary dose; the vaccine's duration of protection; and the cost per dose.

## METHODS

**Figure 1.**
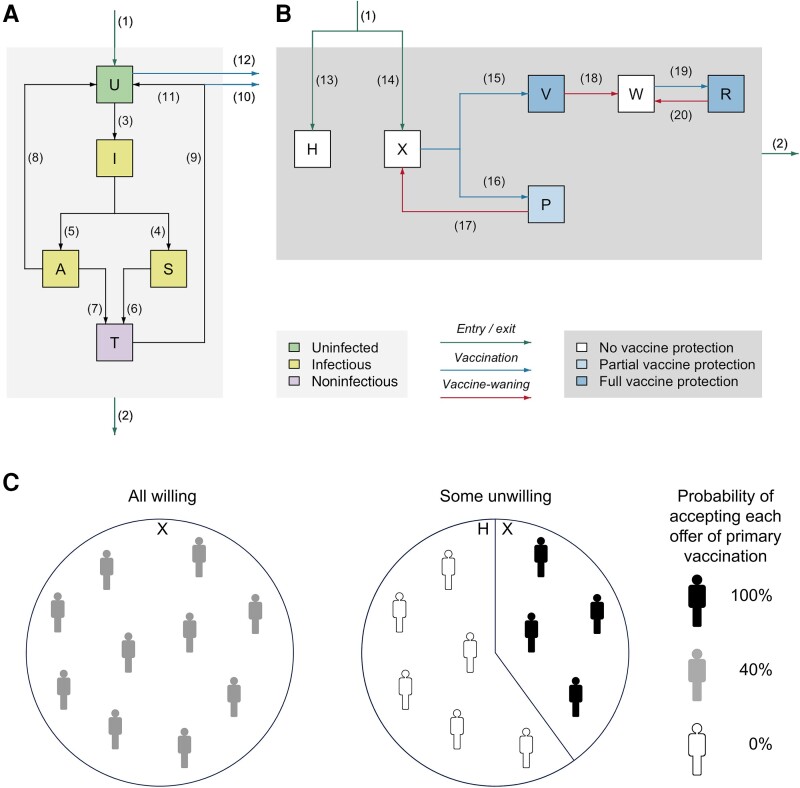
Model structure diagram. *A*, The population is divided into compartments representing different states of infection, with changes of state occurring through various processes. Individuals entering the sexually active population (1) are uninfected U; those leaving the sexually active population through aging (2) leave from any state. Individuals who become infected (3) pass through an incubating state I, before either developing symptoms S (4) or remaining asymptomatic A (5). Symptomatic individuals seek treatment (6) and enter the treatment state T. Asymptomatic infections can be identiﬁed through screening (7), with individuals entering the treatment state T, or there can be natural recovery (8), returning individuals to the uninfected state U. All treated infections are cured (9). Infection does not confer natural immunity, and recovered individuals are as susceptible as those never infected. Under the “vaccination on diagnosis” (VoD) and “vaccination according to risk” (VaR) strategies, vaccination is offered on diagnosis with gonorrhea, with those who accept (10) entering the uninfected state in a vaccine-protected stratum and those who do not accept entering the uninfected state in their current stratum (11). Under the VaR strategy, vaccination is also offered to uninfected individuals in the high sexual activity groups who attend sexual health clinics for screening, with those who accept entering the uninfected state in a vaccine-protected stratum (12) and those who do not accept remaining in the uninfected state in their current stratum. *B*, Stratification of the population representing vaccine sentiment and vaccination status (unwilling to be vaccinated, unvaccinated but willing to be vaccinated, partially vaccinated, fully vaccinated, waned, and revaccinated). A proportion of the population is vaccine-unwilling and resides in the H stratum, with the same proportion of new entrants entering that stratum (13). The remainder of the population is vaccine-willing, and this proportion of new entrants enters the unvaccinated (X) stratum (14). Unvaccinated individuals in stratum X who accept vaccination enter a vaccine-protected stratum (P or V), while those who decline vaccination remain in the unvaccinated stratum. Full primary vaccination comprises 2 doses, with individuals who receive both doses entering the fully vaccinated (V) stratum (15) and those who receive 1 dose entering the partially vaccinated (P) stratum (16). When vaccine protection wanes, partially vaccinated individuals return to the unvaccinated stratum (17), while fully vaccinated individuals enter the waned (W) stratum (18). Individuals in the waned stratum are eligible for single-dose booster vaccination, with those who accept entering the revaccinated (R) stratum (19). When protection from booster vaccination wanes, individuals move from the revaccinated stratum back to the waned stratum (20) and become eligible for revaccination once more. Vaccination is *offered* to unvaccinated individuals (ie, those in strata H and X) and those whose protection has waned (stratum W); however, as those in stratum H do not accept vaccination, vaccine is *administered* only to those in strata X and W. Those in strata V and R have full vaccine protection, which reduces their susceptibility to infection by 40% (95% confidence interval [CI], 22%–53%) [5]; those in stratum P have partial protection, which reduces their susceptibility by 26% (95% CI, 12%–37%) [5]; the other strata have no vaccine protection. There are no flows of individuals between the vaccine-willing strata (X, P, V, W, R) and the vaccine-unwilling stratum (H). Note that there are separate sets of compartments for those in the low and high sexual activity groups, which have identical arrangements of compartments and vaccination-status strata. *C*, Illustration of the 2 vaccine-sentiment scenarios examined. In one of the scenarios (“All-willing”), everyone has the same probability of accepting vaccination each time it is offered, which we varied 0%–100%ie, being “willing” does not necessarily mean that someone accepts vaccination the first time it is offered: some “willing” individuals will decline several offers before finally accepting. In contrast, “unwilling” individuals never accept, no matter how many times vaccination is offered. In the alternative scenario (“Some-unwilling”), there are 2 groups—those who are unwilling to be vaccinated (whose acceptance probability is 0%) and those who are willing to be vaccinated (whose acceptance probability in this scenario is set at 100%)—with the proportion of the population in the vaccine-willing group varied 0%–100%. In the example shown, 40% of people accept vaccination when offered at the start of a program, which corresponds to all people in the All-willing scenario having a 40% probability of accepting vaccination each time it is offered, or 40% of people in the Some-unwilling scenario being willing to be vaccinated (and all of them accepting when first offered), with the remainder always declining vaccination. In the All-willing scenario, everyone starts in stratum X in (*B*) before vaccination begins, and all new entrants to the population enter stratum X. In the Some-unwilling scenario, proportions of the population start in strata H and X, and proportions of new entrants enter strata H and X.

We developed a deterministic transmission-dynamic compartmental model of gonorrhea in MSM in England (Figure 1), extending previous work [17, 26, 27]. The model incorporates treatment from symptom-motivated care-seeking and asymptomatic infection screening, and natural recovery of untreated infection. Heterogeneous sexual behavior is represented by low and high sexual activity groups; the latter have higher rates of partner change and sexual health clinic attendance for screening. We introduced additional stratification to represent (*i*) differential protection from 1- and 2-dose primary vaccination and (*ii*) different population vaccine-sentiment scenarios.

We compared 2 strategies for targeting higher-risk MSM attending sexual health clinics, which is where the vast majority of gonorrhea is managed in England [28]. Under “vaccination on diagnosis” (VoD), vaccination is offered to all individuals diagnosed with gonorrhea infection through symptomatic care-seeking or asymptomatic screening. This pragmatically targets higher-risk individuals because gonorrhea diagnosis is an indicator of risk of future infection [17]. Under the alternative “vaccination according to risk” (VaR) strategy future risk is indicated by current infection with gonorrhea or by patient-reported high numbers of sexual partners (>5/year), so eligibility is expanded beyond VoD to include uninfected individuals in the high-activity group who attend the sexual health clinic for screening. For each targeting strategy, we considered primary vaccination using 1 or 2 doses, with subsequent revaccination using a single booster dose for those who received 2-dose primary vaccination while those who received only 1-dose primary vaccination are offered another course of primary vaccination.

Vaccine sentiment about 4CMenB will vary [23, 25], resulting in segments of the MSM population having different vaccination rates (including zero, for those who are unwilling), but we lack information on the shape of this distribution. So, we examined the effect of different patterns of population vaccine sentiment by comparing 2 scenarios that bracket the range of possibilities (Figure 1*C*). In one of the scenarios (“All-willing”), everyone has the same probability of accepting vaccination each time it is offered, which we varied 0%–100%—that is, being “willing” does not necessarily mean that someone accepts vaccination the first time it is offered: some “willing” individuals will decline several offers before finally accepting. In contrast, “unwilling” individuals never accept no matter how many times vaccination is offered. In the alternative scenario (“Some-unwilling”), there are 2 groups—those who are unwilling to be vaccinated (whose acceptance probability is 0%) and those who are willing to be vaccinated (whose acceptance probability in this scenario is set at 100%)—with the proportion of the population in the vaccine-willing group varied 0%–100%. In the example shown in Figure 1*C*, if 40% of people accept vaccination when offered at the start of a program then this could be because (in the All-willing scenario) all people have a 40% probability of accepting vaccination each time it is offered, or because (in the Some-unwilling scenario) 40% of people are willing to be vaccinated and all of those accept when first offered. The difference is that in the All-willing scenario, if those who declined vaccination were offered it again subsequently then 40% would accept so uptake would remain at 40% through time, whereas in the Some-unwilling scenario the proportion of unvaccinated people who accept vaccination declines below 40% over time because the vaccine-willing group becomes depleted by being vaccinated while the vaccine-unwilling group does not. Second primary dose uptake (which requires an additional clinic visit) was varied 0%–100%. We assumed all individuals offered single-dose booster vaccination accept it (as it is offered when attending clinic for screening or diagnostic testing).

Health-economic analysis took the perspective of sexual health clinics in the National Health Service (NHS), with costs (2020–2021 GB£) and quality-adjusted life-years (QALYs) discounted at 3.5% per annum [29, 30]. We calculated net monetary benefit (NMB) of vaccination over a 10-year time horizon [17] by summing the averted costs of gonorrhea testing and treatment [31] and the monetary value of averted QALY losses and subtracting the vaccination cost. Symptomatic infections incur QALY losses, with a disutility of 0.16 [32] lasting for the time until obtaining care plus half the duration of treatment. QALYs were valued at £20 000 (or £30 000 in sensitivity analysis in the Supplementary Materials) [29, 30]. Each vaccination strategy was compared against a baseline of no vaccination. 4CMenB is currently used by the NHS to protect infants against serogroup B meningococcal disease; the price paid is confidential, but it was estimated this use would be cost-effective at £8 (inflation-adjusted) per dose (excluding administration cost) [33]. A £10 administration fee [17, 34] makes the cost £18/dose administered. We also performed calculations using the £75 list price [35], that is, £85/dose administered.

Epidemiological parameters were previously estimated by calibration, in a Bayesian framework, to data on numbers of tests, numbers of diagnoses, and the proportion of diagnoses that were symptomatic [17]. Primary analysis assumed 1.5 years protection after primary vaccination and 3 years after booster vaccination, durations that JCVI estimates 4CMenB protects infants against serogroup B meningococcal disease [36]. However, as protection lasting 4 years and even 7.5 years has been suggested for adolescents and young adults [37], we considered these durations (for both primary and booster vaccination protection) in scenario analysis. In all analyses, health-economic, epidemiological, and vaccine-effectiveness parameters were varied probabilistically using 1000 parameter sets to account for statistical uncertainty in estimation. Duration of protection was varied deterministically in scenario analyses.

Full model details are shown in the Supplementary Materials. Informed consent was not required as no patients were involved and only aggregate/anonymized data from public sources were used.

## RESULTS

**Figure 2.**
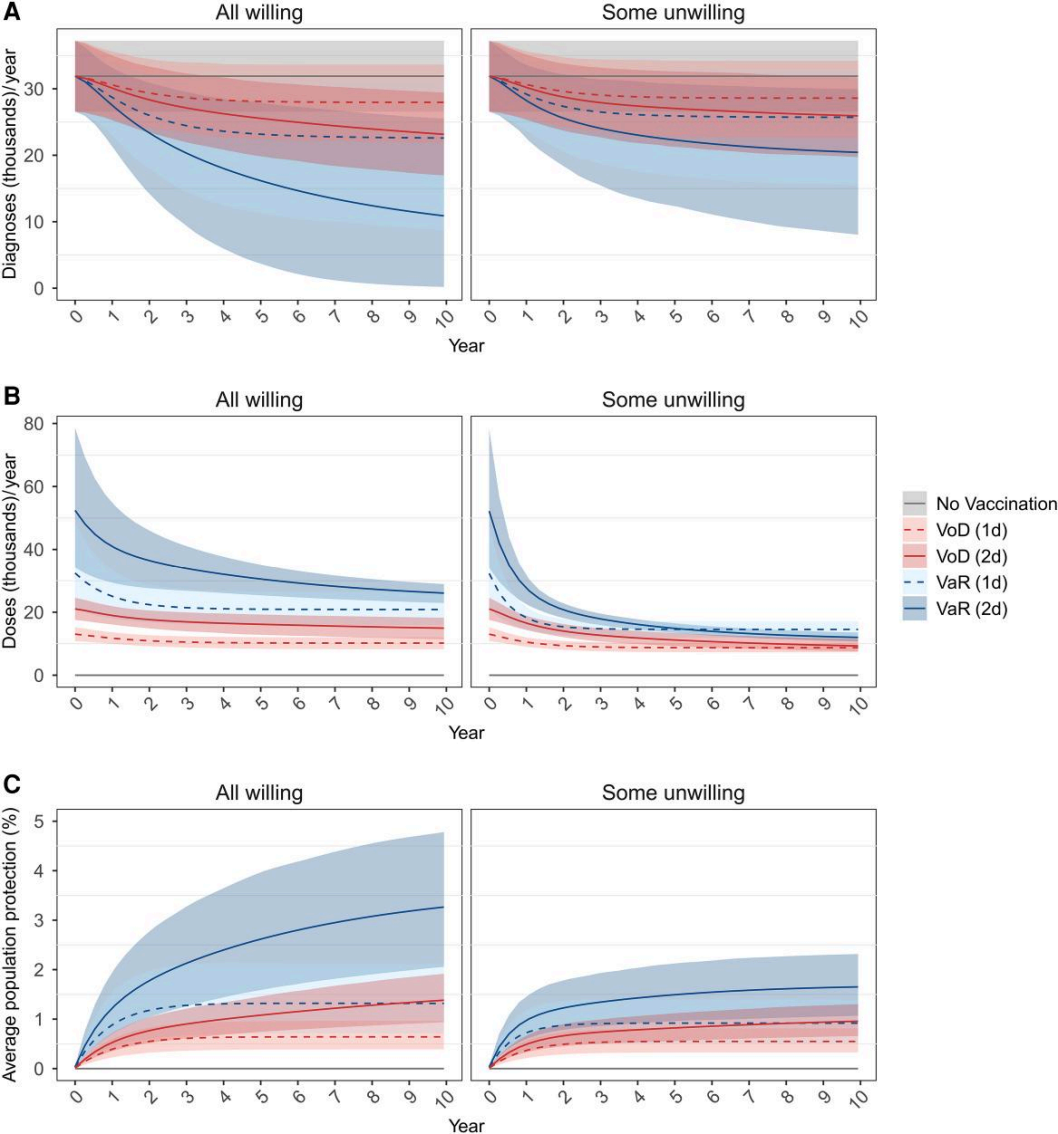
Simulations of gonorrhea transmission in men who have sex with men in England over 10 y after the introduction of vaccination, under different vaccination strategies targeting higher-risk individuals, with different patterns of population vaccine sentiment. Targeting strategies are “vaccination on diagnosis” (VoD) and “vaccination according to risk” (VaR), with 1- and 2-dose primary vaccination strategies denoted “(1d)” and “(2d)”, respectively. In the primary analysis scenario shown, 4CMenB provides protection against gonorrhea for 1.5 and 3 y after 2-dose primary vaccination and single-dose booster vaccination, respectively, with effectiveness of 40% (95% confidence interval [CI], 22%–53%). One-dose primary vaccination has 26% (95% CI, 12%–37%) effectiveness lasting 1.5 y, after which further single doses protect for 1.5 y each. Initial uptake of vaccination (ie, first-dose uptake at the start of the program) is the same in the 2 vaccine-sentiment scenarios: in the All-willing scenario 40.8% (95% credible interval [CrI], 40.6%–41.0%) of those offered vaccination accept the first dose (each time it is offered), whereas in the Some-unwilling scenario 40.8% (95% CrI, 40.6%–41.0%) are willing to be vaccinated, all of whom accept the first dose when offered. In both vaccine-sentiment scenarios, uptake of the second dose (where offered, ie, under the “vaccination on diagnosis” 2-dose strategy [“VoD(2d)”] and the “vaccination according to risk” 2-dose strategy [“VaR(2d)”]) by those who received the first is 61.7% (95% CrI, 61.2%–62.1%), and uptake of single-dose booster vaccination by those offered it is 100%. *A*, Rate of gonorrhea diagnoses. *B*, Rate of vaccine dose administration. *C*, Average protection against gonorrhea of a person in the population, accounting for the proportions of the population with no protection, partial vaccine protection of 26% (95% CI, 12%–37%) from 1-dose primary vaccination and full vaccine protection of 40% (95% CI, 22%–53%) from 2-dose primary vaccination and booster vaccination. (Supplementary Figure S2 shows the proportions of the population with partial and full vaccine protection.) Note that vaccination is mostly given to the high-activity group, which is a minority of the population, so the overall proportion of the population with protection is small. Lines represent means and shaded regions represent 95% CrIs of simulations comparing each vaccination strategy against no vaccination, using 1000 sets of sampled epidemiological parameters.

Figure 2 shows the impact of alternative 4CMenB vaccination strategies with the same uptake as HPV vaccination (ie, 1-dose 40.8% [95% credible interval {CrI}, 40.6%–41.0%], 2-dose 61.7% [95% CrI, 61.2%–62.1%]) [22], and protection lasting 1.5 and 3 years after primary and booster vaccination, respectively. Gonorrhea diagnoses fall rapidly at first, then the rate of decline slows (Figure 2*A*). Vaccine doses administered (Figure 2*B*) decrease over time as the number of eligible unvaccinated people declines with accumulating coverage, and as declining symptomatic cases due to averted transmission result in fewer clinic attendances where vaccination is offered. Vaccination has a greater impact in the All-willing vaccine-sentiment scenario than the Some-unwilling scenario, as fewer vaccinations are administered in the latter. Initially the rate of vaccine administration is the same for both vaccine-sentiment scenarios (for a particular vaccine-targeting strategy) but declines more steeply in the Some-unwilling scenario, due to faster relative depletion of individuals willing to accept vaccination when offered.

Vaccination strategies using VaR targeting have greater impact than those using VoD targeting (Figure 2*A*), due to VaR's broader eligibility increasing the doses administered (Figure 2*B*). Regardless of targeting strategy, 2-dose primary vaccination has more impact than 1-dose vaccination (Figure 2*A*) due to the greater protection given by 2 doses: Figure 2*C* shows the average protection of the population, accounting for the numbers protected by 1- and 2-dose primary vaccination and booster vaccination and the levels of protection conferred. Although more doses are administered with 2-dose primary vaccination, at £18/dose administered the greater vaccination cost is outweighed by greater cost savings on gonorrhea testing and treatment in all targeting strategies and vaccine-sentiment scenarios (Table 1). At £18/dose administered, all vaccination strategies are cost-saving (Table 1). The dominant strategy is VaR (2-dose) if VaR targeting is feasible, otherwise it is VoD (2-dose), regardless of the vaccine-sentiment scenario. Under the Some-unwilling scenario, VoD (2-dose) and VaR (2-dose) strategies have NMBs of £4.22M (95% CrI, .87–9.01) and £9.25M (95% CrI, 1.84–20.65), respectively. Corresponding values under the All-willing scenario are £5.50M (95% CrI, 1.16–11.71) (∼30% greater) and £14.69M (95% CrI, 2.95–29.57) (∼60% greater). Longer durations of protection increase net savings and QALY gains (Supplementary Table S4(b) and S4(c)) and increase the probability of vaccination being cost-effective at higher costs per dose (Supplementary Figures S3 and S4). However, at £85/dose administered, vaccination is only cost-effective if protection lasts 7.5 years, the All-willing vaccine-sentiment scenario applies, and the VaR (2-dose) strategy is used (Supplementary Tables S4(a)–(c) and S6(m)–(x), and Supplementary Figures S3 and S4). Note that a strategy of offering vaccination to all MSM attending sexual health clinics is not cost-effective because it administers many more doses than other strategies (∼3.6 to 4.7 times as much as VaR) and therefore has a much greater cost, but VaR has a very similar impact (compare Supplementary Tables S4(a)–(c) and S5).

**Table 1.** Health-Economic Analysis of Vaccination of Men Who Have Sex With Men in England Over 10 Y, Under Different Vaccination Strategies Targeting Higher-Risk Individuals and Different Patterns of Population Vaccine Sentiment

Vaccination Strategy	Gonorrhea Cases Averted, Thousands		Testing and Treatment Costs Saved, £M	Vaccine Doses Administered, Thousands		Vaccination Costs Incurred, £M	Net Costs Saved, £M	QALYs Gained	NMB, £M
	Undiscounted	Discounted		Undiscounted	Discounted				
All-willing scenario									
VoD (1d)	32.17 (13.06–59.58)	26.46 (10.81–48.88)	3.96 (1.60–7.33)	106.19 (87.53–124.56)	90.31 (74.53–105.88)	1.63 (1.34–1.91)	2.34 (−.13 to 5.88)	30.94 (11.34–63.05)	2.96 (.08–7.10)
VoD (2d)	57.19 (28.06–91.14)	46.44 (22.95–74.16)	6.97 (3.50–11.56)	166.09 (134.26–196.56)	141.68 (115.02–167.11)	2.55 (2.07–3.01)	4.42 (.75–9.29)	54.48 (24.46–100.47)	5.51 (1.35–11.01)
VaR (1d)	74.88 (21.04–139.98)	61.58 (17.42–135.73)	9.22 (2.66–20.33)	220.25 (165.45–284.95)	187.72 (140.64–243.41)	3.38 (2.53–4.38)	5.84 (−.05 to 16.16)	72.05 (19.00–168.30)	7.28 (.39–19.50)
VaR (2d)	139.98 (51.77–238.91)	113.68 (42.06–194.70)	17.05 (5.98–30.51)	323.60 (260.53–390.85)	278.32 (222.60–338.78)	5.01 (4.01–6.10)	12.04 (1.88–24.62)	133.23 (45.68–262.74)	14.71 (2.80–29.01)
Some-unwilling scenario									
VoD (1d)	27.57 (11.02–51.73)	22.73 (9.12–42.63)	3.40 (1.35–6.45)	92.00 (77.87–107.06)	78.41 (66.43–91.14)	1.41 (1.20–1.64)	1.99 (−.15 to 5.14)	26.57 (9.51–54.45)	2.52 (.06–6.21)
VoD (2d)	43.16 (20.25–71.74)	35.30 (16.65–58.55)	5.29 (2.52–9.08)	121.47 (102.67–143.24)	104.91 (88.85–123.57)	1.89 (1.60–2.22)	3.41 (.58–7.31)	41.34 (17.83–78.39)	4.23 (.97–8.78)
VaR (1d)	52.20 (16.06–111.06)	43.14 (13.35–91.89)	6.46 (2.01–14.12)	156.99 (126.77–187.53)	134.30 (108.26–160.76)	2.42 (1.95–2.89)	4.04 (−.11 to 11.37)	50.45 (14.41–117.27)	5.05 (.21–13.47)
VaR (2d)	85.16 (30.68–163.82)	69.77 (25.28–133.77)	10.46 (3.65–20.65)	178.30 (153.48–198.45)	156.05 (133.78–174.48)	2.81 (2.41–3.14)	7.65 (1.23–17.56)	81.68 (28.43–172.89)	9.28 (1.77–21.09)

Results are mean values and 95% credible intervals (CrIs) of simulations comparing each vaccination strategy against no vaccination, using 1000 sets of sampled epidemiological and health-economic parameters, with a QALY valued at £20 000. In the primary analysis scenario shown, 4CMenB provides protection against gonorrhea for 1.5 and 3 y after 2-dose primary vaccination and single-dose booster vaccination, respectively, with effectiveness of 40% (95% confidence interval [CI], 22%–53%). One-dose primary vaccination has 26% (95% CI, 12%–37%) effectiveness lasting 1.5 y, after which further single doses protect for 1.5 y each. Initial uptake of vaccination (ie, first-dose uptake at the start of the program) is the same in the 2 vaccine-sentiment scenarios: In the All-willing scenario, 40.8% (95% CrI, 40.6%–41.0%) of those offered vaccination accept the first dose (each time it is offered), whereas in the Some-unwilling scenario 40.8% (95% CrI, 40.6%–41.0%) are willing to be vaccinated, all of whom accept the first dose when offered. In both vaccine-sentiment scenarios, uptake of the second dose (where offered, ie, under the VoD [2d] and VaR [2d] strategies) by those who received the first dose is 61.7% (95% CrI, 61.2%–62.1%), and uptake of single-dose booster vaccination by those offered it is 100%. Vaccination costs £18/dose administered. All values are discounted at 3.5% per annum except where stated (cases averted and vaccine doses administered have both discounted and undiscounted numbers reported). Note how for each vaccine-sentiment scenario (All-willing and Some-unwilling), each vaccination strategy saves more money and gains more QALYs than the one above and therefore dominates the one above.

Abbreviations: M, millions; NMB, net monetary benefit; QALY, quality-adjusted life-year; VaR, vaccination according to risk; VoD, vaccination on diagnosis; 1d, 1-dose primary vaccination strategy; 2d, 2-dose primary vaccination strategy.

**Figure 3.**
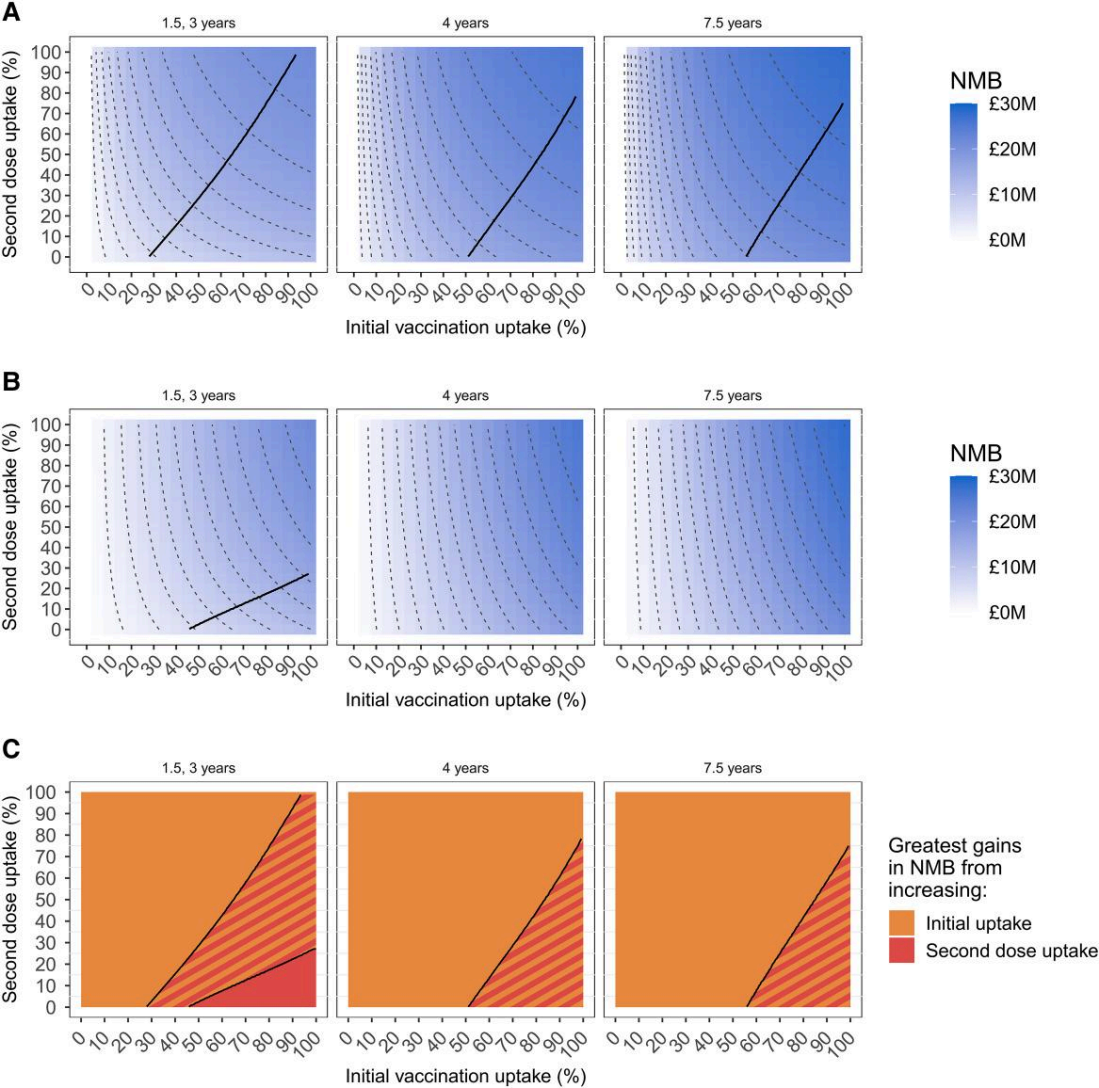
Health-economic value of vaccination against gonorrhea of men who have sex with men in England over 10 y under the “vaccination according to risk” targeting strategy, comparing different duration(s) of vaccine protection and population vaccine-sentiment scenarios. *A* and *B*, Heatmaps of the mean net monetary benefit (NMB) of vaccination, with vaccine costing £18/dose administered, under different vaccine-sentiment scenarios: All-willing (*A*) and Some-unwilling (*B*). Horizontal axes show the initial uptake of vaccination (ie, first-dose uptake at the start of the program) and vertical axes show uptake of the second primary dose; a single-dose primary vaccination strategy corresponds to the bottom edge. Contour lines show points of equal NMB, in multiples of £2M. Where present, the solid black diagonal line divides the map into regions where NMB is increased more by an increment in initial vaccine uptake (moving horizontally left to right on the map) (ie, the upper-left region, where contour lines are closer together moving horizontally) and where NMB is increased more by an increment in second-dose uptake (moving vertically upwards on the map) (ie, the lower-right region, where contour lines are closer together moving vertically); on maps without a solid black line, NMB always increased more by an increment in initial vaccine uptake. *C*, Diagram comparing the positions of the black line in (*A*) and (*B*), showing where NMB is increased more by an increment in initial vaccine uptake than an increment in second-dose uptake in both vaccine-sentiment scenarios (solid orange), where NMB is increased more by an increment in second-dose uptake than an increment in initial vaccine uptake in both vaccine-sentiment scenarios (solid red), and where the vaccine-sentiment scenarios differ regarding whether NMB is increased more by an increment in initial vaccine uptake or in second-dose uptake (stripes). In the striped region NMB is increased more by an increment in initial vaccine uptake in the Some-unwilling scenario, but is increased more by an increment in second-dose uptake in the All-willing scenario. Primary analysis (in which protection lasts 1.5 and 3 y after primary and booster vaccination, respectively) is shown in the left column of panels, with longer durations of protection in the middle column (4 y after primary and booster vaccination) and right column (7.5 y after primary and booster vaccination). Simulations compare vaccination against no vaccination, using 1000 sets of sampled epidemiological and health-economic parameters, with a quality-adjusted life-year valued at £20 000. Note that for given duration(s) of protection, the All-willing and Some-unwilling heatmaps are identical on their left and right edges, because the left edge corresponds to no one being vaccinated, while the right edge corresponds to all individuals who are offered vaccination accepting it.

**Figure 4.**
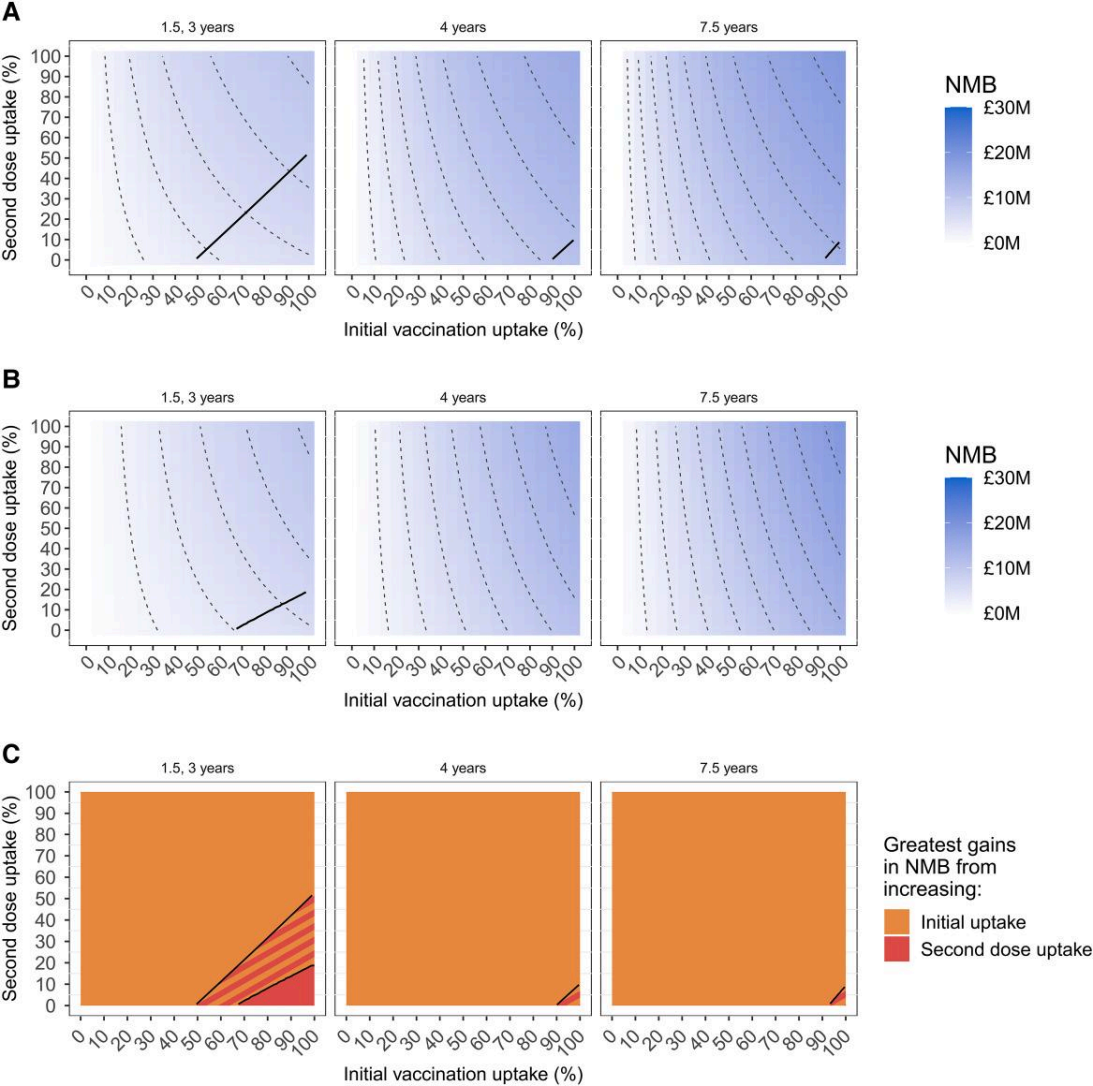
Health-economic value of vaccination against gonorrhea of men who have sex with men in England over 10 y under the vaccination on diagnosis (VoD) targeting strategy, comparing different duration(s) of vaccine protection and population vaccine-sentiment scenarios. Details are as described for Figure 3 but considering the VoD targeting strategy.

We compared the health-economic value (NMB) of 1- and 2-dose primary vaccination strategies targeting higher-risk individuals, and how this is affected by uptake of vaccination (ie, first-dose uptake), uptake of the second dose (where offered), duration of protection, vaccine-sentiment scenario, and targeting strategy (Figures 3 and 4), if vaccination costs £18/dose administered. Single-dose primary vaccination strategies are represented by the bottom edges of the plots, which correspond to no one receiving a second primary dose (although repeat vaccination after protection has waned is still offered). Under all circumstances examined, the mean NMB (and 95% CrI, Supplementary Table S6(a)–(l)) is positive, so vaccination is cost-effective (and in fact cost-saving) regardless of uptake. Greater uptake of vaccination always corresponds to greater NMB, as does greater uptake of the second primary dose (where offered), so offering 2-dose primary vaccination is always superior to offering 1-dose. Longer duration of protection increases NMB because more infections are averted, and fewer doses are used due to longer gaps between repeat vaccination. VaR targeting (Figure 3) always has a higher NMB than VoD (Figure 4), for any combination of first- and second-dose uptake, duration of protection, and vaccine-sentiment scenario.

Promotional campaigns are often used to increase vaccination coverage, so to inform setting objectives and budgets we quantify how much NMB is increased by an increment in first- or second-dose uptake. In the All-willing vaccine-sentiment scenario (Figures 3*A* and 4*A*) contour lines are closer together at lower values of initial uptake of vaccination, indicating that an increment in first-dose uptake adds much more value when first-dose uptake is low. For example, if vaccine protection lasts 1.5 and 3 years after primary and booster vaccination, respectively, then under VaR targeting if second-dose uptake were 20% then an increment in first-dose uptake from 20% to 30% would increase NMB by £2.1M, whereas an increment from 80% to 90% would increase NMB by only £0.6M (Figure 3*A*, left panel). In contrast, in the Some-unwilling scenario (Figures 3*B* and 4*B*) the relationship between first-dose uptake and NMB is approximately linear—contour lines are approximately evenly spaced—so an increment in first-dose uptake increases NMB by a similar amount for all starting levels of first-dose uptake. Using the same example as before, an increment in first-dose uptake from 20% to 30% would increase NMB by £1.7M, whereas an increment from 80% to 90% would increase NMB by £1.2M (Figure 3*B*, left panel). The relationship between second-dose uptake and NMB is approximately linear for both vaccine-sentiment scenarios (Figures 3*A* and 3*B* and 4*A* and 4*B*). For a given level of first- and second-dose uptake, NMB is higher in the All-willing scenario than the corresponding Some-unwilling scenario (Figures 3*A* and 3*B* and 4*A* and 4*B*), except when first-dose uptake is 0% or 100% (where numbers of people vaccinated are the same in the 2 vaccine-sentiment scenarios). Supplementary Figures S5–S8 present transects through the heatmaps.

Now we examine how the pattern of population vaccine sentiment affects whether NMB is increased more by incrementing first- or second-dose uptake. In the primary analysis (where vaccine protection lasts 1.5 and 3 years after primary and booster vaccination, respectively), if first-dose uptake is low and second-dose uptake is high (upper-left of Figures 3 and 4) then NMB is increased more by incrementing first-dose uptake than second-dose uptake, and the opposite applies if first-dose uptake is high and second-dose uptake is low (lower-right of Figures 3 and 4). However, the dividing line between these 2 regions shifts toward higher first-dose uptake and lower second-dose uptake (to the right and down) in the Some-unwilling vaccine-sentiment scenario compared with the All-willing scenario for both VaR (Figure 3*C*) and VoD (Figure 4*C*). This means there is a range of combinations of first- and second-dose uptake (striped regions in Figures 3*C* and 4*C*) where the pattern of variation in population vaccine sentiment determines whether NMB is increased more by incrementing first-dose uptake or second-dose uptake. For each vaccine-sentiment scenario, the dividing line is shifted to the right and down for the VoD-targeting strategy compared with the VaR-targeting strategy (compare Figure 3A with Figure 4A, and compare Figure 3B with Figure 4B).

Longer durations of protection favor prioritizing incrementing first-dose uptake, with the dividing line shifted progressively to the right and down for 4 and 7.5 years (Figures 3*C* and 4*C*). Under the All-willing vaccine-sentiment scenario, for VaR targeting the shift of the line is modest for VaR targeting (Figure 3*A*) but large for VoD targeting (Figure 4*A*). Under the Some-unwilling scenario, for both VaR and VoD targeting, NMB always increases more with increasing uptake of first dose rather than second dose (Figures 3*B* and 4*B*).

**Figure 5.**
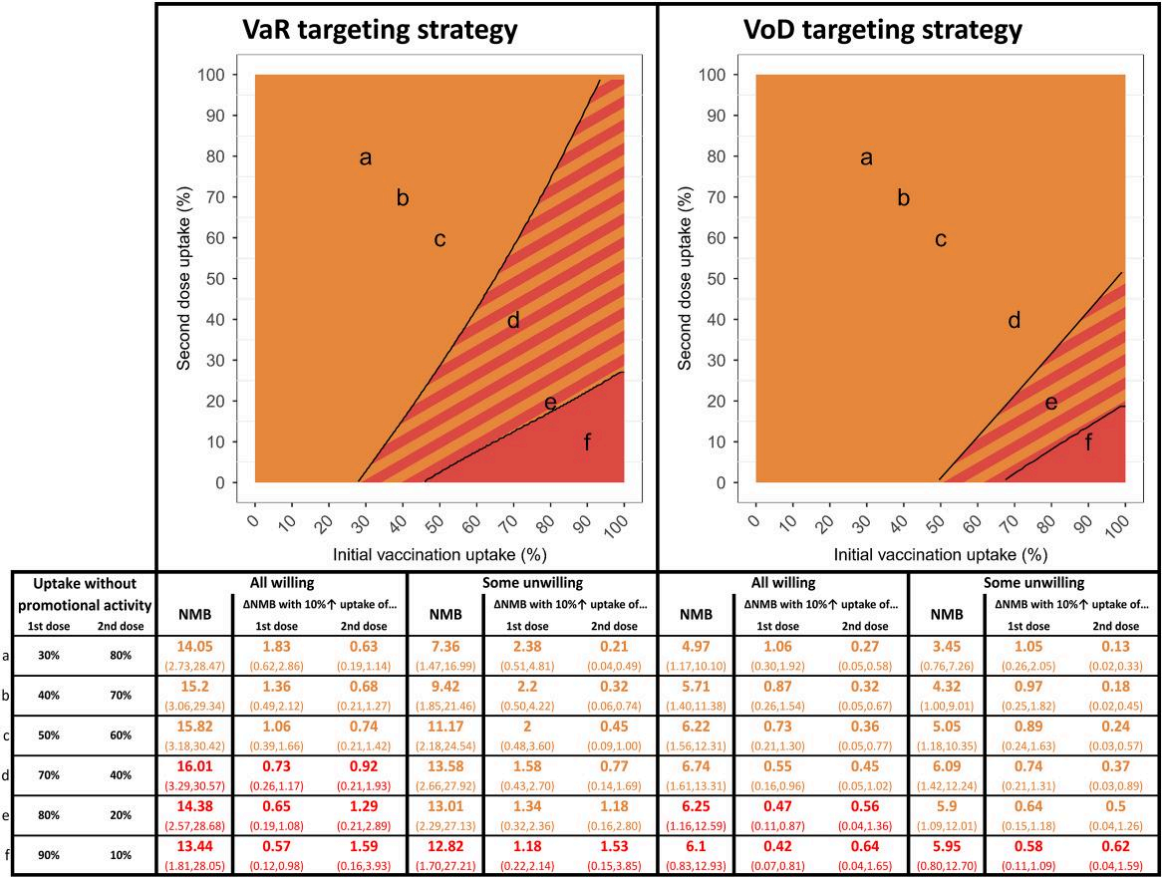
Increase in the value of vaccination of men who have sex with men in England over 10 y resulting from a 10% increase in uptake of first or second dose due to promotional activity, under different vaccine-targeting strategies targeting higher-risk individuals, different population vaccine-sentiment scenarios, and for different levels of uptake without that promotional activity. Selected examples are examined, corresponding to the points a–f on the plots. Solid colors show where net monetary benefit (NMB) is increased more by an increment in initial vaccine uptake (ie, first-dose uptake at the start of the program) than an increment in second-dose uptake in both vaccine-sentiment scenarios (orange), where NMB is increased more by an increment in second-dose uptake than an increment in initial vaccine uptake in both vaccine-sentiment scenarios (red), and where the vaccine-sentiment scenarios differ regarding whether NMB is increased more by an increment in initial vaccine uptake or in second-dose uptake (stripes). In the scenario analyzed, 4CMenB provides protection against gonorrhea for 1.5 and 3 y after 2-dose primary vaccination and single-dose booster vaccination, respectively, with effectiveness of 40% (95% CI, 22%–53%). One-dose primary vaccination has 26% (95% CI, 12%–37%) effectiveness lasting 1.5 y, after which further single doses protect for 1.5 y each. In both vaccine-sentiment scenarios, uptake of the second dose by those who received the first dose is 61.7% (95% credible interval [CrI], 61.2%–62.1%), and uptake of single-dose booster vaccination by those offered it is 100%. Vaccination costs £18/dose administered. A quality-adjusted life-year is valued at £20 000. All values are discounted at 3.5% per annum. Results are mean values and 95% CrIs of 1000 simulations. Abbreviation: VaR, vaccination according to risk; VoD, vaccination on diagnosis.

Vaccine sentiment not only affects whether an increment in first- or second-dose uptake has a larger effect on NMB, but also affects the size of the increase in NMB, which in turn determines the maximum size of an appropriate budget for promotional activity. Figure 5 shows examples in which a 10% increment in first-dose uptake increases NMB ranging from £0.42M (95% CrI, .08–.83) to £2.38M (95% CrI, .54–4.89), and a 10% increment in second-dose uptake increases NMB ranging from £0.13M (95% CrI, .05–.23) to £1.58M (95% CrI, .55–2.52), depending on the targeting strategy and population vaccine sentiment. For points a–c in Figure 5, NMB is always increased more by an increment in first-dose uptake than second-dose uptake, but for these examples the value added by a 10% increment in first-dose uptake ranges from £1.06M (95% CrI, .4–1.66) to £1.84M (95% CrI, .65–2.90), £2.01M (95% CrI, .49–3.63) to £2.38M (95% CrI, .54–4.89), £0.73M (95% CrI, .20–1.33) to £1.07M (95% CrI, .30–1.99), or £0.89M (95% CrI, .23–1.71) to £1.05M (95% CrI, .26–2.13), depending on the targeting strategy and population vaccine sentiment (Figure 5).

To enable calculation of the health-economic values of different-sized increases in first- and second-dose uptake from different starting values, Supplementary Table S6 reports NMB of vaccination at different levels of first- and second-dose uptake, for both targeting strategies and both vaccine-sentiment scenarios, with vaccination costing £18 and £85 per dose administered.

## DISCUSSION

Based on observational estimates of effectiveness, we find that vaccination of higher-risk MSM with 4CMenB to protect against gonorrhea is likely to be cost-effective (and in fact cost-saving) at the UK cost per dose, under both conservative (“Some-unwilling”) and optimistic (“All-willing”) population vaccine-sentiment scenarios, at any level of uptake of the first and (where offered) the second primary dose, with higher uptake of either dose being more beneficial. The implications are, first, a vaccination program does not need to achieve a minimum level of uptake to be cost-effective. Second, offering 2-dose primary vaccination is superior to offering 1-dose, even if uptake of the second dose is low, because for those who receive only 1 dose the protection is worth the cost to the health service and the additional protection of the second dose to those who receive it is worth the additional cost. Third, the health-economic value of a vaccination program would be increased by promotional campaigns that increase uptake, provided the promotional activity costs less than the increase in value it achieves. Fourth, the greater coverage of VaR targeting means it has greater impact and NMB than VoD. However, VaR might be operationally challenging to implement because it requires inquiring about sexual behavior, which can be a sensitive subject [17], hence our also considering VoD. Importantly, the longer the duration of vaccine protection the greater the NMB due to a lower frequency of repeat vaccination being required to maintain protection of the population, thus reducing costs and averting more infections.

This work quantifies in health-economic terms the value of behavioral science in health-system planning and health-promotion activities, which can inform allocation of research funding to the discipline. Uptake of MSM-specific vaccination is typically low, and “[improvement] to an adequate level may require additional support and investment for public health programmes” [21]. We quantified how population variation in vaccine sentiment affects the impact and health-economic value of vaccination—with NMB being approximately 30% higher under VoD targeting and approximately 60% higher under VaR in the All-willing scenario (Table 1)—and affects optimal program implementation strategies. While successful promotional activities will often increase uptake of both first and second primary vaccine doses, different approaches might be optimal for promoting each. Promoting first-dose uptake likely entails raising awareness of the availability, benefits, and safety of vaccination so that individuals have considered vaccination before being offered it in the sexual health clinic. Promoting second-dose uptake involves reminding and encouraging those who accepted a first dose during a sexual health clinic visit for diagnostic testing or screening to make a special trip to receive another vaccination. We identified (Figures 3*C* and 4*C*) under what circumstances (ie, targeting strategy, duration of vaccine protection, and uptake of first and second primary doses) promoting first- or second-dose uptake produces the larger increase in NMB. Striped regions of Figures 3*C* and 4*C* show circumstances where better understanding of the pattern of population vaccine sentiment would be most valuable, because here it determines whether promoting first- or second-dose uptake is most beneficial. We quantified how much value is added by increasing uptake of first or second doses under different circumstances (Figure 5, Supplementary Table S6) to inform decisions about appropriate budgets for promotional activity.

Previous modeling analyses of gonorrhea vaccination (see, eg, [17, 27]) implicitly assumed the “All-willing” pattern of population vaccine sentiment, which is the most optimistic scenario regarding vaccination impact and cost-effectiveness. In addition to examining a range of scenarios representing uncertainty in vaccine sentiment in combination with uncertainty in the duration of protection, other strengths of the work are that the model incorporates heterogeneity in sexual behavior and realistic vaccine-targeting strategies. Our conclusions are robust to uncertainty in epidemiological parameters, which we account for formally using the same Bayesian framework as our previous work [17], which used more data sources than previous studies [26, 27, 38].

As our model accounts for the protection offered by 1 primary dose [5], the estimated cost-effectiveness of vaccination is increased compared with our previous analysis which assumed that 2 doses are required for any protection [17], because in that analysis people who received only 1 dose incurred a cost to the NHS for no benefit. However, our estimation of the value of gonorrhea vaccination is still conservative. First, as in our previous work [17], we assume that imperfect vaccine protection is “leaky” (giving partial protection to all vaccinees rather than complete protection to a proportion and no protection to the remainder) [39]. Second, lack of suitable data means the burden of gonorrhea is not fully quantified, and potential future increases in that burden caused by antimicrobial resistance (AMR) are unknown and hence not included in the calculation, but are likely to be substantial [17, 19]. Vaccination can combat emergence of AMR by reducing (*i*) infection prevalence and (*ii*) selection pressure from treatment [27, 40]. These considerations mean we underestimate the full value of vaccination in benefiting health and reducing net healthcare costs.

Protecting higher-risk MSM against gonorrhea using 4CMenB is likely cost-saving at the UK cost per dose, regardless of the level of uptake, based on observational estimates of effectiveness. If the evidence from the multiple trials due to report in the coming years [12–14] differs from the multiple observational studies reported to date [5–12], then detailed examination of reasons for differences will be required. If trials find lower protection, then note that we have previously found vaccination could be cost-effective even if protection is only 20%, lasts only 2 years, and requires 2 primary doses [17]. As the duration of protection is an important determinant of vaccination program impact, value, and optimal promotional activity, we recommend that adolescent and adult 4CMenB trials and vaccination programs—whether for meningitis, gonorrhea, or both—incorporate follow-up using record linkage to quantify the duration of protection by monitoring rates of gonorrhea diagnosis over time (eg, [5, 7, 8]). Additionally, optimization of vaccination requires a feasibility study of VaR targeting, and quantification of variation in vaccine sentiment (eg, [25]) to inform promotional interventions [21]. JCVI's advice to provide 4CMenB to UK MSM [15] offers an opportunity to perform these studies and test our model.

## Supplementary Data

Supplementary materials are available at *The Journal of Infectious Diseases* online (http://jid.oxfordjournals.org/). Supplementary materials consist of material provided by the author that are published to benefit the reader. The posted materials are not copyedited. The contents of all supplementary materials are the sole responsibility of the authors. Questions or messages regarding errors should be addressed to the author.

## Supplementary Material

jiae123_Supplementary_Data

## Notes

***Disclaimer.*** The views expressed are those of the authors and not necessarily those of the UK Department of Health and Social Care; Foreign, Commonwealth and Development Office; European Union; UK Medical Research Council (MRC); National Institute for Health and Care Research (NIHR); UK Health Security Agency (UKHSA); or Wellcome Trust.

***Financial support*.** This work was supported by the UK MRC Centre for Global Infectious Disease Analysis (grant number MR/X020258/1); this award comes under the Global Health EDCTP3 Joint Undertaking. This work was also supported by the NIHR Health Protection Research Unit in Modelling and Health Economics, which is a partnership between the UKHSA, Imperial College London, and the London School of Hygiene & Tropical Medicine (grant code NIHR200908). D. N. is funded by an MRC PhD studentship. L. K. W. is funded by the Wellcome Trust (grant number 218669/Z/19/Z). For the purpose of open access, the authors are required by the funders to apply a Creative Commons Attribution (CC BY) license to any Author Accepted Manuscript version arising.
