# Supplementary material for: Cost-effectiveness of 4CMenB Vaccination Against Gonorrhea: Importance of Dosing Schedule, Vaccine Sentiment, Targeting Strategy, and Duration of Protection
Source: J Infect Dis. 2024 Apr 17;231(1):71–83. doi: 10.1093/infdis/jiae123 (PMC11793026; doi:10.1093/infdis/jiae123)
Supplement: jiae123_Supplementary_Data [file jiae123_supplementary_data.zip › Supplementary materials.pdf]

Supplementary materials for

Cost-effectiveness of 4CMenB vaccination against gonorrhea: importance of dosing schedule, vaccine sentiment, targeting strategy, and duration of protection

Contents

1 Model details 2

1.1 Model of gonorrhea transmission and vaccination . . . 2

1.1.1 Transmission . . . 2

1.1.2 Vaccination strata . . . 2

1.1.3 Vaccine targeting . . . 3

1.1.4 Compartmental model equations . . . 5

1.1.5 Compartmental model parameter values . . . 8

1.2 Scenarios analyzed . . . 9

1.3 Outputs of interest . . . 10

1.3.1 Cases diagnosed . . . 10

1.3.2 Vaccine doses administered . . . 10

1.3.3 Population-level vaccine protection . . . 10

1.3.4 Health-economic analysis . . . 10

2 Supplemental results 13

2.1 Proportions of the MSM population in England with different levels of vaccine protection over 10 years after the introduction of vaccination, under different vaccination strategies targeting higher-risk individuals, with different patterns of population vaccine sentiment (Figure S2) . . . 13

2.2 Health-economic analysis of vaccination of MSM in England over 10 years, under different strategies targeting higher-risk individuals and patterns of population vaccine sentiment, with different durations of vaccine protection, and costs per dose (Table S4; Figures S3,S4) . . . 14

2.2.1 Cost-effectiveness acceptability curves (Figure S4) . . . 19

2.3 Health-economic analysis of vaccination of MSM in England over 10 years, offering Vaccination-on-Attendance (VoA) under different patterns of population vaccine sentiment, with different durations of vaccine protection, and costs per dose (Table S5) . . . 21

2.4 Health-economic value of vaccination against gonorrhea of MSM in England over 10 years under different strategies targeting higher-risk individuals, comparing different durations of vaccine protection and population vaccine-sentiment scenarios (Figures S5-S8) . . . 23

2.5 Health-economic value of vaccination for pairwise combinations of 1st and 2nd dose uptake under different strategies targeting higher-risk individuals, population vaccine-sentiment scenarios, durations of vaccine protection, and costs per dose (Table S6) . . . 28

1 Model details

1.1 Model of gonorrhea transmission and vaccination

1.1.1 Transmission

We developed a deterministic transmission-dynamic compartmental model of gonorrhea in men who have sex with men (MSM) based on our previous work [1]. The model structure stratifies the population of MSM in England (total size N) by vaccination status, level of sexual activity (j), and gonorrhea infection status (Figure S1). In the model, MSM enter the population upon sexual debut (α per year), with a proportion (q_L) joining the low sexual activity group, and the remainder ($q_H = 1 - q_L$) joining the high-activity group. Individuals entering the model population are uninfected (U). Acquisition of infection in the absence of vaccination occurs at rate $\lambda_j(t)$ through sexual contact with a contagious individual ($C = I + A + S$). Newly-infected individuals pass through an incubating state (I) at rate σ, after which a proportion ψ develop symptoms (S) while the rest remain asymptomatic (A). Symptomatic individuals seek treatment at rate μ. Asymptomatic individuals may be diagnosed and treated through sexual health screening (i.e. testing in the absence of symptoms), at rate η_j, with individuals in the high sexual activity group being screened more frequently, or else recover naturally at rate ν. Individuals diagnosed with gonorrhea are treated and recover at rate ρ, after which they are again susceptible to infection (U). Infection does not confer natural immunity and recovered individuals are equally as susceptible as those never infected. The rate of screening (η_j) also applies to uninfected individuals but of course does not result in a change of state. (See subsection 1.1.4 Compartmental model equations, governing Equations (3-33)).

The force of infection, $\lambda_j(t)$, i.e. the rate of acquisition of infection (before accounting for any vaccine protection) for an uninfected individual in sexual activity group j, depends on the rate of partner change per year in each sexual activity group (c_j); the level of assortativity in sexual mixing between groups (ϵ, where $\epsilon = 0$ denotes proportionate mixing and $\epsilon = 1$ denotes fully assortative contact [2]); the prevalence of contagious individuals in each group ($C_j(t)/N_j(t)$); and the constant rate of transmission (β). The force of infection is calculated as follows:

$$\lambda_j(t) = c_j\beta\left(\epsilon\frac{C_j(t)}{N_j(t)} + (1-\epsilon)\left(\sum_{i\in\{L,H\}}\pi_i(t)\frac{C_i(t)}{N_i(t)}\right)\right) \quad (1)$$

Where $\pi_j(t) = \frac{c_j N_j(t)}{\sum_{i\in\{L,H\}} c_i N_i(t)}$ is the proportion of all partnerships in the population that involve a member of group j.

The rate of screening (i.e. testing in the absence of symptoms, which applies to asymptomatic and uninfected individuals), η_j, depends on the sexual activity group, with individuals in the high sexual activity group being screened more frequently.

1.1.2 Vaccination strata

The model incorporates vaccination with one or two doses of 4CMenB, which we assume offers 'leaky', or 'degree-type', protection [3], reducing the probability of infection upon sexual contact with an infectious partner, so it is still possible, but less likely, for vaccine-protected individuals to become infected. Vaccination status does not affect an infected individual's progression through stages of infection or their infectiousness.

There are six strata representing vaccine sentiment and vaccination status (i). We consider scenarios in which a proportion (h) of the population is unwilling to be vaccinated; these individuals reside in the H stratum.

The proportion of the population that is vaccine-willing ($1-h$), initially resides in the Unvaccinated stratum (X). The proportion of those individuals who accept the 1st dose of vaccine when offered is r_1, and the proportion who return to clinic to obtain a 2nd dose is r_2. Therefore, the proportion of individuals who receive a single vaccine dose is $r_1(1 - r_2)$. They become partially-vaccinated (P) and experience a proportionate reduction of e_p in their susceptibility to infection, until vaccine protection wanes after a mean duration D_P when the individual returns to the Unvaccinated stratum (X) and their susceptibility returns to its pre-vaccination level. They can then receive another single dose to be partially-vaccinated once more (P) or two doses to become fully-vaccinated (V).

Unvaccinated individuals (X) who receive two vaccine doses (a proportion r_1r_2 of those individuals offered vaccination) become fully-vaccinated (V) and experience a proportionate reduction of e_v in their susceptibility to infection, until vaccine protection wanes after a mean duration D_V when the individual enters the Waned stratum (W) and their susceptibility returns to its pre-vaccination level. Those in (W) retain immunological

memory from having been fully-vaccinated previously, which means that a single booster dose (accepted by proportion of r_b of those offered it) restores protection to the fully-vaccinated level (e_v) with the individuals entering the revaccinated stratum (R). This protection lasts for a mean duration D_R until the individuals return to the Waned stratum (W). Protection can be restored after waning by a single booster dose an indefinite number of times.

Transitions between strata are shown in Figure S1, where the presence of j subscript indicates that vaccine targeting applies to the high sexual activity group only. Individuals entering the model population are all uninfected and enter strata H and X in proportions h and $(1-h)$, respectively.

The infectious population in group j incorporates individuals across all six vaccination-status strata (H, X, P, V, W, and R), so that:

$$C_j(t) = \sum_{i \in \{H,X,P,V,W,R\}} \left(I_j^i(t) + A_j^i(t) + S_j^i(t) \right) \tag{2}$$

1.1.3 Vaccine targeting

As described above, individuals in the Unvaccinated (X) and Waned (W) strata may receive vaccination, and we consider two approaches to targeting higher-risk individuals. Under the "Vaccination-on-Diagnosis" (VoD) strategy, vaccination is offered to individuals in both low- and high-activity groups who are diagnosed with gonorrhea infection, either through care-seeking by symptomatic individuals (S) or screening of those with asymptomatic infection (A). Under the alternative "Vaccination-according-to-Risk" (VaR) strategy, vaccination is offered to individuals in both low- and high-activity groups who are diagnosed with gonorrhea infection (the same as under VoD), plus uninfected individuals (U) in the high-activity group who attend the sexual-health clinic for screening (denoted "Vaccination-on-Screening", VoS, in Figure S1). We also consider "Vaccination-on-Attendance" (VoA), with vaccination offered to all individuals tested for gonorrhea (regardless of whether they are infected or not).

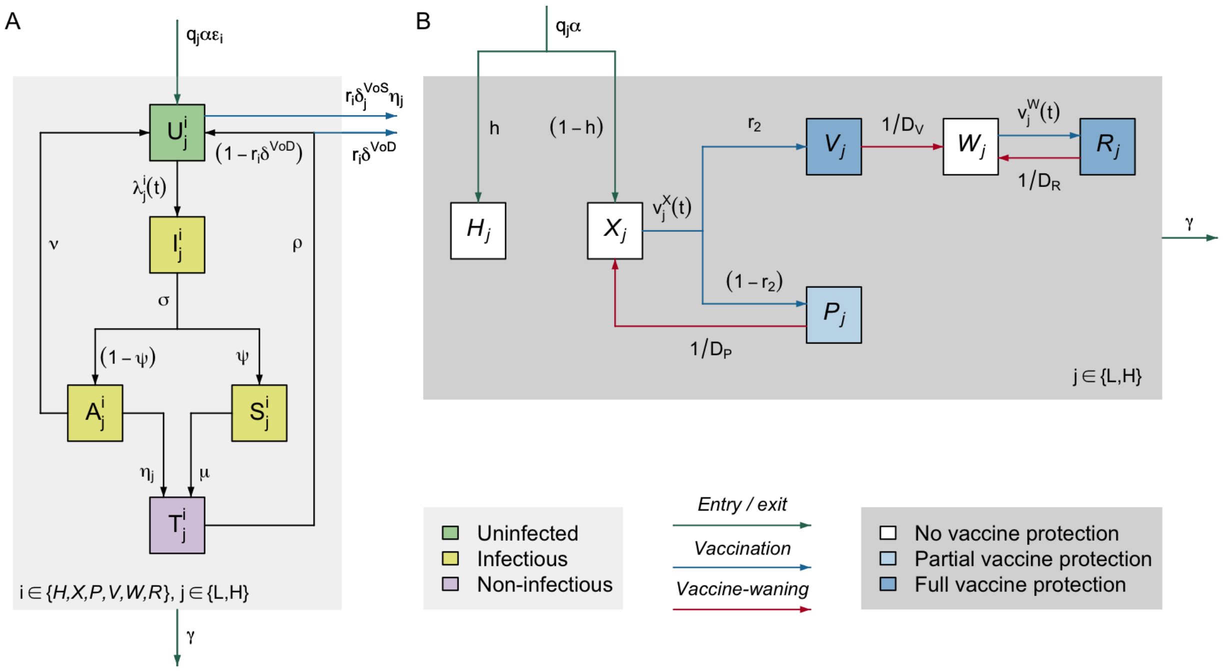

Figure S1: Model structure diagram. This is the same as in the main paper except labelled algebraically. In panel (A) the infection states are Uninfected (U), Incubating (I), Asymptomatic (A), Symptomatic (S), Treatment (T). In panel (B) the vaccination-status strata are Unwilling to be vaccinated (H), Unvaccinated but willing to be vaccinated (X), Partially-vaccinated (P), Fully-vaccinated (V), Waned (W), Revaccinated (R). Arrows are labelled with per-capita rates, and symbols are the same as in the equations of the model, except that $q_j\alpha\epsilon_i$, $q_j\alpha$, and $v_j^i(t)$ are volume flows (i.e. numbers of individuals per unit time, entering the U_j^i compartments of the relevant strata) and the symbols r_i, ε_i, and $v_j^i(t)$ are not in the model equations but are used just to label this figure. $r_i = r_1$ in stratum X, $r_i = r_b$ in stratum W, $r_i = 0$ in other strata. The term ε_i indicates the proportion of new entrants entering stratum H ($\varepsilon_H = h$), stratum X ($\varepsilon_X =$ (1-h)), and the other strata ($\varepsilon_i = 0$ for $i \in \{P, V, W, R\}$). Volume flows $v_j^X(t) = (U_j^X \eta_j \delta_j^{VoS} + T_j^X \rho \delta_j^{VoD})r_1$ and $v_j^W(t) = (U_j^W \eta_j \delta_j^{VoS} + T_j^W \rho \delta_j^{VoD})r_b$.

1.1.4 Compartmental model equations

We describe the model depicted in Figure S1 with differential equations. Note that in this type of model durations in states and strata are exponentially-distributed. Each compartment $k \in \{U, I, A, S, T\}$ (i.e. Uninfected, Incubating, Asymptomatic, Symptomatic, Treatment) is stratified by sexual activity group $j \in \{L, H\}$ (i.e. Low, High) and according to vaccination status i: unwilling (H), unvaccinated (X), partial vaccine protection (P), full vaccine protection (V), waned (W), revaccinated (R). Table S1 shows how values of δ_j^{VoS} and δ^{VoD} switch between different vaccine-targeting strategies in the compartmental model equations(3-33): no vaccination, "Vaccination-on-Diagnosis" (VoD), "Vaccination-according-to-Risk" (VaR: VoD plus "Vaccination-on-Screening" (VoS) in the high sexual activity group), and "Vaccination-on-Attendance" (VoA: VoD plus "Vaccination-on-Screening" (VoS) in both sexual activity groups). Definitions of model other parameters are in Table S2.

Table S1: Vaccine-targeting parameters

	Approach	δ_L^{VoS}	δ_H^{VoS}	δ^{VoD}
Baseline	No vaccination	0	0	0
VoD	Vaccination-on-Diagnosis	0	0	1
VaR	Vaccination-according-to-Risk	0	1	1
VoA	Vaccination-on-Attendance	1	1	1

$$\frac{dU_j^X(t)}{dt} = \frac{U_j^P(t)}{D_P} + q_j\alpha(1-h) - (\lambda_j(t) + \delta_j^{\text{VoS}} r_1\eta_j + \gamma)U_j^X(t) + \nu A_j^X(t) + (1-\delta^{\text{VoD}} r_1)\rho T_j^X(t) \tag{3}$$

$$\frac{dI_j^X(t)}{dt} = \frac{I_j^P(t)}{D_P} + \lambda_j(t)U_j^X(t) - (\sigma+\gamma)I_j^X(t) \tag{4}$$

$$\frac{dA_j^X(t)}{dt} = \frac{A_j^P(t)}{D_P} + (1-\psi)\sigma I_j^X(t) - (\nu+\eta_j+\gamma)A_j^X(t) \tag{5}$$

$$\frac{dS_j^X(t)}{dt} = \frac{S_j^P(t)}{D_P} + \psi\sigma I_j^X(t) - (\mu+\gamma)S_j^X(t) \tag{6}$$

$$\frac{dT_j^X(t)}{dt} = \frac{T_j^P(t)}{D_P} + \eta_j A_j^X(t) + \mu S_j^X(t) - (\rho+\gamma)T_j^X(t) \tag{7}$$

$$\frac{dU_j^P(t)}{dt} = \delta_j^{\text{VoS}} r_1(1-r_2)\eta_j U_j^X(t) + \delta^{\text{VoD}} r_1(1-r_2)\rho T_j^X(t) + \nu A_j^P(t) + \rho T_j^P(t) - \left((1-e_p)\lambda_j(t) + \frac{1}{D_P} + \gamma\right)U_j^P(t) \tag{8}$$

$$\frac{dI_j^P(t)}{dt} = (1-e_p)\lambda_j(t)U_j^P(t) - \left(\sigma + \frac{1}{D_P} + \gamma\right)I_j^P(t) \tag{9}$$

$$\frac{dA_j^P(t)}{dt} = (1-\psi)\sigma I_j^P(t) - \left(\nu + \eta_j + \frac{1}{D_P} + \gamma\right)A_j^P(t) \tag{10}$$

$$\frac{dS_j^P(t)}{dt} = \psi\sigma I_j^P(t) - \left(\mu + \frac{1}{D_P} + \gamma\right)S_j^P(t) \tag{11}$$

$$\frac{dT_j^P(t)}{dt} = \eta_j A_j^P(t) + \mu S_j^P(t) - \left(\rho + \frac{1}{D_P} + \gamma\right)T_j^P(t) \tag{12}$$

$$\frac{dU_j^V(t)}{dt} = \delta_j^{\text{VoS}} r_1 r_2\eta_j U_j^X(t) + \delta^{\text{VoD}} r_1 r_2\rho T_j^X(t) + \nu A_j^V(t) + \rho T_j^V(t) - \left((1-e_v)\lambda_j(t) + \frac{1}{D_V} + \gamma\right)U_j^V(t) \tag{13}$$

$$\frac{dI_j^V(t)}{dt} = (1-e_v)\lambda_j(t)U_j^V(t) - \left(\sigma + \frac{1}{D_V} + \gamma\right)I_j^V(t) \tag{14}$$

$$\frac{dA_j^V(t)}{dt} = (1-\psi)\sigma I_j^V(t) - \left(\nu + \eta_j + \frac{1}{D_V} + \gamma\right)A_j^V(t) \tag{15}$$

$$\frac{dS_j^V(t)}{dt} = \psi\sigma I_j^V(t) - \left(\mu + \frac{1}{D_V} + \gamma\right)S_j^V(t) \tag{16}$$

$$\frac{dT_j^V(t)}{dt} = \eta_j A_j^V(t) + \mu S_j^V(t) - \left(\rho + \frac{1}{D_V} + \gamma\right)T_j^V(t) \tag{17}$$

$$\frac{dU_j^W(t)}{dt} = \frac{U_j^V(t)}{D_V} + \frac{U_j^R(t)}{D_R} - (\lambda_j(t) + \delta_j^{\text{VoS}} r_b\eta_j + \gamma)U_j^W(t) + \nu A_j^W(t) + (1-\delta^{\text{VoD}} r_b)\rho T_j^W(t) \tag{18}$$

$$\frac{dI_j^W(t)}{dt} = \frac{I_j^V(t)}{D_V} + \frac{I_j^R(t)}{D_R} + \lambda_j(t)U_j^W(t) - (\sigma+\gamma)I_j^W(t) \tag{19}$$

$$\frac{dA_j^W(t)}{dt} = \frac{A_j^V(t)}{D_V} + \frac{A_j^R(t)}{D_R} + (1-\psi)\sigma I_j^W(t) - (\nu+\eta_j+\gamma)A_j^W(t) \tag{20}$$

$$\frac{dS_j^W(t)}{dt} = \frac{S_j^V(t)}{D_V} + \frac{S_j^R(t)}{D_R} + \psi\sigma I_j^W(t) - (\mu+\gamma)S_j^W(t) \tag{21}$$

$$\frac{dT_j^W(t)}{dt} = \frac{T_j^V(t)}{D_V} + \frac{T_j^R(t)}{D_R} + \eta_j A_j^W(t) + \mu S_j^W(t) - (\rho+\gamma)T_j^W(t) \tag{22}$$

(23)

$$\frac{dU_j^R(t)}{dt} = \delta_j^{\mathrm{VoS}} r_b \eta_j U_j^W(t) + \delta^{\mathrm{VoD}} r_b \rho T_j^W(t) + \nu A_j^R(t) + \rho T_j^R(t) - \left((1-e_v)\lambda_j(t) + \frac{1}{D_R} + \gamma\right) U_j^R(t) \tag{24}$$

$$\frac{dI_j^R(t)}{dt} = (1-e_v)\lambda_j(t) U_j^R(t) - \left(\sigma + \frac{1}{D_R} + \gamma\right) I_j^R(t) \tag{25}$$

$$\frac{dA_j^R(t)}{dt} = (1-\psi)\sigma I_j^R(t) - \left(\nu + \eta_j + \frac{1}{D_R} + \gamma\right) A_j^R(t) \tag{26}$$

$$\frac{dS_j^R(t)}{dt} = \psi\sigma I_j^R(t) - \left(\mu + \frac{1}{D_R} + \gamma\right) S_j^R(t) \tag{27}$$

$$\frac{dT_j^R(t)}{dt} = \eta_j A_j^R(t) + \mu S_j^R(t) - \left(\rho + \frac{1}{D_R} + \gamma\right) T_j^R(t) \tag{28}$$

$$\frac{dU_j^H(t)}{dt} = q_j \alpha h - (\lambda_j(t) + \gamma) U_j^H(t) + \nu A_j^H(t) + \rho T_j^H(t) \tag{29}$$

$$\frac{dI_j^H(t)}{dt} = \lambda_j(t) U_j^H(t) - (\sigma + \gamma) I_j^H(t) \tag{30}$$

$$\frac{dA_j^H(t)}{dt} = (1-\psi)\sigma I_j^H(t) - (\nu + \eta_j + \gamma) A_j^H(t) \tag{31}$$

$$\frac{dS_j^H(t)}{dt} = \psi\sigma I_j^H(t) - (\mu + \gamma) S_j^H(t) \tag{32}$$

$$\frac{dT_j^H(t)}{dt} = \eta_j A_j^H(t) + \mu S_j^H(t) - (\rho + \gamma) T_j^H(t) \tag{33}$$

1.1.5 Compartmental model parameter values

The model is parameterized in the same manner as our previous model [1], representing a constant-sized population of $N = 600,000$ sexually-active MSM. Demographic, behavioral, and natural history parameters (Table S2) are taken from Whittles et al. 2022,[1], which used a Bayesian evidence-synthesis framework applied to surveillance data from the Genitourinary Medicine Clinic Activity Dataset (GUMCAD) [4], and the Gonococcal Resistance to Antimicrobials Surveillance Programme (GRASP) [5]. GUMCAD reports annual gonorrhea tests and diagnoses from all STI clinics in England, and GRASP is a sentinel surveillance system which reports the proportion of diagnosed infections that are symptomatic. As in our previous work [1, 6], to account for uncertainty in the natural history parameters we use 1000 natural history parameter sets sampled from the posterior distribution. Each of these 1000 parameter sets is assigned a sampled value from a beta distribution for each vaccine-effectiveness parameter (e_p, e_v).

Uptake of the first vaccine dose when offered is the product of the proportion of individuals who are vaccine-willing, (1-h), and their probability of accepting vaccination when offered, r_1. For example if 40% of people accept vaccination when offered at the start of a program then this could be because (in the *"All-willing"* scenario) all people have a 40% probability of accepting vaccination on a given occasion, or because (in the *"Some-unwilling"* scenario) 40% of people are willing to be vaccinated and all of those accept when offered. The difference is that in the *"All-willing"* scenario if those who declined vaccination were offered it again subsequently then 40% would accept so uptake would remain at 40% through time, whereas in the *"Some-unwilling"* scenario the proportion of unvaccinated people who accept vaccination declines below 40% over time because the vaccine-willing group becomes depleted by being vaccinated whilst the vaccine-unwilling group does not. In the *"All-willing"* scenario, (1-h)=100% and r_1 is varied. In the *"Some-unwilling"* scenario, r_1=100% and (1-h) is varied. To compare the two vaccine-sentiment scenarios, parameter values are assigned so that the product (1-h)r_1 is the same, so that at the start of the program the initial first-dose uptake is the same.

• In analysis where initial uptake of vaccination (i.e. initial first-dose uptake at the start of the program) is the same as for HPV vaccination of MSM [i.e. 1st dose 40.8%(95%CrI:40.6,41.0), 2nd dose 61.7%(95%CrI:61.2,62.1)] [7] uncertainty is represented by assigning (i) a sampled value from a beta distribution for 1st-dose uptake to r_1 or (1-h), depending on the vaccine-sentiment scenario, and (ii) a sampled value from a beta distribution for 2nd-dose uptake to r_2, for each of the 1000 natural history parameter sets.

• In other analysis, 1st- and 2nd-dose uptake values are varied deterministically 0-100% for each of (i) r_1 or (1-h), and (ii) r_2, respectively, with the same point values assigned to all 1000 natural history parameter sets.

Uptake of the second primary dose by those who received the first dose is r_2, which we vary. In all analyses we assume all who are offered booster vaccination accept it (r_b = 100%), since eligible individuals are those who were previously fully-vaccinated (so they made an additional clinic visit to obtain the 2nd primary dose), and booster vaccination is offered whilst attending clinic for screening or diagnostic testing so is convenient.

The duration of 4CMenB's protection against gonorrhea is uncertain. The UK Joint Committee on Vaccination and Immunisation (JCVI) estimates that 4CMenB protects infants against serogroup B meningococcal disease for 18 months after two-dose primary vaccination and 36 months after single-dose booster vaccination [8]. However, protection lasting 4 years and even 7.5 years has been suggested for adolescents and young adults [9]. Therefore, as before [1], we consider three scenarios for duration of protection against gonorrhea: (i) 1.5 years after primary vaccination and 3 years after booster vaccination, (ii) 4 years after both primary vaccination and booster vaccination, (iii) 7.5 years after both primary vaccination and booster vaccination.

Table S2: Transmission-dynamic model parameters. Transition rate parameters (σ, μ, ν, ρ) are presented on an annual basis, giving a mean time to transition of $365/\theta$ days. Vaccine effectiveness parameters (e_p, e_v) are sampled from beta distributions; vaccine-uptake parameters (h, r_1, r_2) are varied deterministically or sampled from beta distributions, depending on the analysis.

	Definition	Value(s)	Source
$N(t_0)$	Population size of England MSM	600,000	[1, 6]
α	Annual population entrants (at age 15)	12,000	[10]
$\frac{1}{\gamma}$	Years spent in the sexually-active population	50	Ages 15-65
q_L	Proportion of the population in group L	85%	[11]
q_H	Proportion of the population in group H	15%	$1 - q_L$
c_L	Annual rate of partner change in group L	0.6	[1, 6]
c_H	Annual rate of partner change in group H	15.6	[1, 6]
β	Probability of transmission per-partnership	0.410 (95%CrI: $0.266, 0.650$)	[1]
ϵ	Level of assortativity in sexual mixing	0.570 (95%CrI: $0.040, 0.986$)	[1]
ψ	Probability that incident infection is symptomatic	0.150 (95%CrI: $0.0737, 0.238$)	[1]
σ	Rate of leaving incubating state ($I \to S$ or A)	99.9 (95%CrI: $56.1, 176.0$)	[1]
μ	Rate of seeking treatment due to symptoms ($S \to T$)	218 (95%CrI: $92.9, 521$)	[1]
ν	Rate of natural recovery ($A \to U$)	3.08 (95%CrI: $1.60, 6.03$)	[1]
η_L	Rate of asymptomatic screening in group L	0.319 (95%CrI: $0.238, 0.384$)	[1]
η_H	Rate of asymptomatic screening in group H	0.717 (95%CrI: $0.398, 1.165$)	[1]
ρ	Rate of recovery after treatment ($T \to U$)	54.0 (95%CrI: $43.6, 66.4$)	[1]
h	Proportion of population that is willing to be vaccinated	(1-h) = 0-100% or 40.8% (95%CrI: $40.6, 41.0$)	-
r_1	Proportion of vaccine-willing individuals accepting (i.e. receiving) 1st dose when offered	0-100% or 40.8% (95%CrI: $40.6, 41.0$)	[7]
r_2	Proportion accepting (i.e. receiving) 2nd dose when offered	0-100% or 61.7% (95%CrI: $61.2, 62.1$)	[7]
r_b	Proportion accepting (i.e. receiving) booster dose when offered	100%	-
e_p	Effectiveness of partial vaccination	0.26 (95%CI: $0.12, 0.37$)	[12]
e_v	Effectiveness of full (and booster) vaccination	0.40 (95%CI: $0.23, 0.53$)	[12]
D_P	Duration of protection: partial primary vaccination	$1.5, 4, 7.5$ years	[8, 9]
D_V	Duration of protection: full primary vaccination	$1.5, 4, 7.5$ years	[8, 9]
D_R	Duration of protection: booster vaccination	$3, 4, 7.5$ years	[8, 9]

1.2 Scenarios analyzed

We perform deterministic forward simulations of gonorrhea transmission in MSM using our model (described in Equations (3-33)). The model is run to equilibrium before each vaccination scenario is implemented. We compare the number of gonorrhea cases averted, number of vaccine doses administered, and net monetary benefit, of vaccination vs no vaccination, for:

- primary vaccination with 1 or 2 doses, under two vaccine-targeting strategies (VoD, VaR); with
- either (i) varying levels of uptake (0-100%) of the 1st primary vaccination dose and of the 2nd primary vaccination dose (where offered), or (ii) the levels of uptake observed for HPV vaccination of MSM (i.e. 1st dose 40.8%(95%CrI:40.6,41.0), 2nd dose 61.7%(61.2,62.1)) [7]; under
- different assumed durations of protection: (i) 1.5 years after primary vaccination and 3 years after booster vaccination, (ii) 4 years after both primary and booster vaccination, or (iii) 7.5 years after both primary and booster vaccination; and
- two alternative patterns of population vaccine sentiment: (i) *"All-willing":* everyone has the same probability of accepting vaccination when offered; or (ii) *"Some-unwilling":* there are two groups – those who are willing or unwilling to be vaccinated – with vaccination acceptance probabilities of 100% and 0%, respectively.

All computation is performed in R version 4.0.3. The differential equation model is implemented using the R package, odin version 1.3.3 [13].

1.3 Outputs of interest

1.3.1 Cases diagnosed

For each vaccination-status stratum $i \in \{H, X, P, V, W, R\}$ and calendar year t, the total number of diagnosed cases, $Y_D^i(t)$; the number of symptomatic and asymptomatic diagnoses, $Y_S^i(t), Y_A^i(t)$; and the number of uninfected patients screened for gonorrhea, $Y_U^i(t)$, are given by:

$$Y_D^i(t) = \sum_{j \in \{L,H\}} \int_t^{t+1} \rho T_j^i(\tau) d\tau \tag{34}$$

$$Y_S^i(t) = \sum_{j \in \{L,H\}} \int_t^{t+1} \mu S_j^i(\tau) d\tau \tag{35}$$

$$Y_A^i(t) = \sum_{j \in \{L,H\}} \int_t^{t+1} \eta_j(\tau) A_j^i(\tau) d\tau \tag{36}$$

$$Y_U^i(t) = \sum_{j \in \{L,H\}} \int_t^{t+1} \eta_j(\tau) U_j^i(\tau) d\tau \tag{37}$$

1.3.2 Vaccine doses administered

Vaccination is administered in sexual health clinics to unvaccinated MSM who are vaccine-willing and to those whose protection has waned, i.e. those in strata $i \in \{X, W\}$. Full protection requires primary vaccination with two doses. A proportion r_1 of those in stratum X who are offered vaccination receive the 1st dose and proportion r_2 of those receive a 2nd dose, so the proportions receiving 1- and 2-dose primary vaccination are $r_1(1 - r_2)$ and $r_1 r_2$, respectively. A single booster dose is administered to proportion r_b of those in stratum W who are offered it. We denote the total number of vaccine doses administered in each stratum in year t as $V_i(t)$:

$$V^X(t) = r_1(1 + r_2)\big(\delta^{\text{VoD}} Y_D^X(t) + \delta_j^{\text{VoS}} Y_U^X(t)\big) \tag{38}$$

$$V^W(t) = r_b\big(\delta^{\text{VoD}} Y_D^W(t) + \delta_j^{\text{VoS}} Y_U^W(t)\big) \tag{39}$$

Therefore, the total number of doses administered in year t across the whole population is:

$$V(t) = V^X(t) + V^W(t) \tag{40}$$

1.3.3 Population-level vaccine protection

For illustrative purposes (main paper Figure 2) we calculate the average protection against gonorrhea of a person in the population, $L(t)$, accounting for the proportions of the population with no protection, partial vaccine protection, and full vaccine protection. This is the sum of the proportion of the population in each of the vaccine-protected strata ($N^i(t)/N(t)$) multiplied by the vaccine protection (e_p, e_v) experienced by individuals in the relevant stratum:

$$L(t) = \frac{e_p N^P(t) + e_v(N^V(t) + N^R(t))}{N(t)} \tag{41}$$

1.3.4 Health-economic analysis

We assess the impact and health-economic value of vaccination compared to a baseline of no vaccination. We calculate the number of diagnosed cases in year t across all vaccination-status strata
$i \in \{H, X, P, V, W, R\}, Y_D(t) = \sum_i Y_D^i(t)$. This is compared to the baseline ($\hat{Y}_D(t)$) to give the total cases averted over M years, where $M - 1$ describes the Mth year of the series:

$$\sum_{t=0}^{M-1} \hat{Y}_D(t_0 + t) - Y_D(t_0 + t) \tag{42}$$

Similarly, using the definition set out in Equation 40, the number of vaccine doses administered over M years, relative to the baseline of no vaccination is:

$$\sum_{t=0}^{M-1} V(t_0 + t) \tag{43}$$

Costs and QALYs are calculated as in Whittles et al. 2022 [1]; a brief description of the calculations is reproduced below for the convenience of the reader. All prices are adjusted to 2020-21 GB £ values using the Hospital and Community Health Services (HCHS) index and NHS Cost Inflation Index (NHSCII) [14].

Table S3: Health-economic parameters.

	Description	Mean (95%CI)	Distribution	Source
w^U	Cost of initial test (£)	88.35 $(57.57, 123.16)$	Gamma$(88.35, 0.2)$*	[15]
w^S	Cost of investigating symptoms (£)	21.72 $(14.59, 31.90)$	Gamma$(21.72, 0.2)$*	[15]
w^T	Cost of treatment (£)	79.82 $(52.55, 117.50)$	Gamma$(79.82, 0.2)$*	[15]
w^Q	Value of a QALY (£)	20,000	-	[16]
w^{ToC}	Cost of Test-of-Cure (ToC) (£)	44.28$(28.83, 63.99)$	Gamma$(44.28, 0.2)$*	[15]
p^{ToC}	Proportion returning for ToC	0.57$(0.54, 0.60)$	Beta$(552, 419)$	[5, 6]
d^S	QoL disutility of symptoms	0.160$(0.136, 0.182)$	Pert$(0.128, 0.16, 0.192)$	[17, 18]
z^T	Reduction in cost of treatment at initial visit	14%	-	[15]

**Gamma distributions parameterized in terms of mean and standard deviation*

For each year, t, we sum across all vaccination-status strata, $i \in \{H, X, P, V, W, R\}$, to calculate the total number of symptomatic diagnoses, $Y_S(t) = \sum_i Y_S^i(t)$; the total number of asymptomatic diagnoses, $Y_A(t) = \sum_i Y_A^i(t)$; and the total number of uninfected patients screened for gonorrhea $Y_U(t) = \sum_i Y_U^i(t)$.

QALY loss is the product of the QoL disutility of symptoms and the average duration of symptoms. This average duration is assumed to be the time until obtaining care $(1/\mu)$ plus half the duration of treatment $(1/2\rho)$, with uncertainty in those parameters being represented by the posterior distribution. We assess health-economic value with a QALY valued at £20,000 or £30,000, as is standard UK practice [16].

The total healthcare costs for uninfected $(W_U(t))$, asymptomatic $(W_A(t))$, and symptomatic individuals $(W_S(t))$ in year t are:

$$W_U(t) = w^U Y_U(t) \tag{44}$$

$$W_A(t) = \left(w^U + w^T + p^{\text{ToC}} w^{\text{ToC}}\right) Y_A(t) \tag{45}$$

$$W_S(t) = \left(w^U + w^S + (1 - z^T) w^T + p^{\text{ToC}} w^{\text{ToC}}\right) Y_S(t) \tag{46}$$

The total costs for all categories of individual combined in year t is:

$$W(t) = W_U(t) + W_A(t) + W_S(t) \tag{47}$$

The total value of QALY losses for symptomatic individuals $(W_S(t))$ in year t discounted to its present value at rate $d = 3.5\%$ [16] are:

$$Q(t) = d^S \left(\frac{1}{\mu} + \frac{1}{2\rho}\right) w^Q Y_S(t) \left(1 + d^{-(t+0.5)}\right) \tag{48}$$

We derive the incremental net costs including the cost of vaccination (NC) over M years, from $W(t)$ for the given vaccination scenario, $\hat{W}(t)$ for the no-vaccination baseline, and cost of vaccination (i.e. the product of the number of doses administered (V) and the cost per dose (ξ)), with future years discounted to the present value at rate $d = 3.5\%$ [16].

$$NC = \sum_{t=0}^{M-1} \left(W(t_0 + t) - \hat{W}(t_0 + t) + \xi V(t_0 + t)\right) \left(1 + d^{-(t+0.5)}\right) \tag{49}$$

We calculate the net monetary benefit (NMB) of vaccination over M years, which is the monetary value of QALYs gained (with a QALY valued at £20,000) minus the net costs of vaccination. Note that if vaccination is cost-saving then NC is negative, which increases NMB.

$$\text{NMB} = \sum_{t=0}^{M-1} \Big(Q(t_0+t) - \hat{Q}(t_0+t)\Big) - NC \tag{50}$$

We also calculate the probability that the net monetary benefit with a QALY valued at £30,000 is positive because JCVI's Code of Practice recommends that the probability of the cost per QALY gained exceeding £30,000 should be no more than 10% for an intervention to be recommended [19]. For clarity, we use "NMB" to refer to net monetary benefit with a QALY valued at £20,000 and "$\text{NMB}_{£30\text{k}}$" to refer to net monetary benefit with a QALY valued at £30,000.

4CMenB is currently used by the NHS in the UK to protect infants against serogroup B *Neisseria meningitidis* but the price paid is confidential. Therefore, as in Whittles et al. 2022 [1], we use two alternative costs per dose administered: £18 and £85. The higher cost corresponds to the UK list price of £75 per dose [20] plus a £10 administration cost [21]. The lower cost is based on the observation that 4CMenB was estimated to be cost-effective for use in infants at £8 per dose (inflation-adjusted) [22], excluding administration cost.

The health-economic analysis is summarized in the box below.

Target population, setting and location: men who have sex with men (MSM) in England.
Perspective: sexual health clinics in the National Health Service (NHS).
Comparisons: vaccination (with 1- and 2-dose primary vaccination schedules) in sexual health clinics under two alternative targeting strategies vs no vaccination, under two scenarios representing patterns of population vaccine sentiment: (i) *"All-willing":* everyone has the same probability of accepting vaccination when offered (which we vary, 0-100%); or (ii) *"Some-unwilling":* there are two groups – those who are willing or unwilling to be vaccinated – with vaccination acceptance probabilities of 100% and 0%, respectively, and we vary the population proportions in each group, 0-100%. The proportion of vaccinees receiving a 2nd primary dose is varied, 0-100%.
Time horizon: 10 years.
Discount rate: 3.5%p.a.
Health outcomes: QALY losses due to symptoms.
Measurement of effectiveness: Vaccine effectiveness 26%(95%CI:12,37) after 1 primary dose, 40%(23,53) after 2 primary doses or single-dose booster vaccination; duration of protection varied in the range 1.5 – 7.5 years.
Currency: 2020-21 GB £.
Willingness-to-pay: £20,000/QALY, with £30,000/QALY used in sensitivity analysis.
Net Monetary Benefit: sum of the averted costs of gonorrhea testing and treatment, and the monetary value of averted QALY losses, minus the cost of vaccination.

2 Supplemental results

2.1 Proportions of the MSM population in England with different levels of vaccine protection over 10 years after the introduction of vaccination, under different vaccination strategies targeting higher-risk individuals, with different patterns of population vaccine sentiment (Figure S2)

In the main paper Figure 2C shows the average protection against gonorrhea of a person in the population, accounting for the proportions of the population with no protection, partial vaccine protection of 26%(95%CI:12,37) from one-dose primary vaccination and full vaccine protection of 40%(22,53) from two-dose primary vaccination and booster vaccination. Here, Figure S2 shows the proportions of the population with partial and full vaccine protection. For the 1-dose vaccination strategies, the only level of protection available is partial, arising from single-dose primary vaccination. For the 2-dose strategies, some individuals have partial protection because they did not receive a 2nd dose whilst others have full protection from two-dose primary vaccination or single-dose revaccination following two-dose primary vaccination.

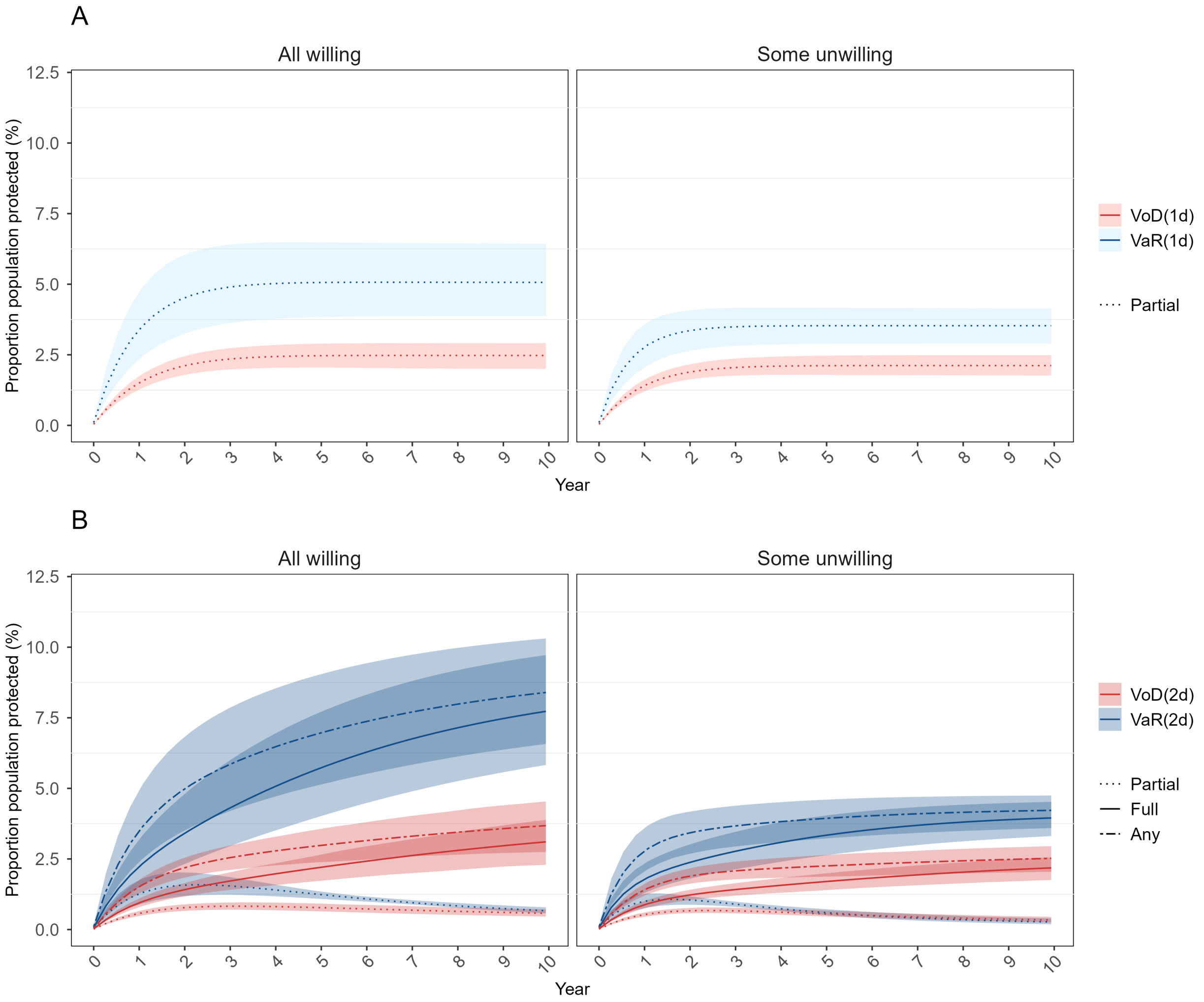

Figure S2: Proportions of the MSM population in England with different levels of vaccine protection over 10 years after the introduction of vaccination, under different vaccination strategies targeting higher-risk individuals, with different patterns of population vaccine sentiment. The scenarios shown are the same as in Figure 2 in the main paper. (A) Proportions of the population with partial vaccine protection of 26%(95%CI:12,37) from one-dose primary vaccination under the VoD(1-dose) and VaR(1-dose) strategies. (B) Proportions of the population with partial vaccine protection of 26%(12,37) from one-dose primary vaccination, full vaccine protection of 40%(22,53) from two-dose primary vaccination and booster vaccination, and any level of protection (i.e. the sum of the proportions with partial or full protection) under the VoD(2-dose) and VaR(2-dose) strategies.

2.2 Health-economic analysis of vaccination of MSM in England over 10 years, under different strategies targeting higher-risk individuals and patterns of population vaccine sentiment, with different durations of vaccine protection, and costs per dose (Table S4; Figures S3,S4)

Table 1 in the main paper presents health economic analysis of different 4CMenB vaccination strategies, where initial uptake of vaccination (i.e. initial first-dose uptake at the start of the program) is the same as for HPV vaccination of MSM (i.e. 1st dose 40.8%(95%CrI:40.6,41.0), 2nd dose 61.7%(61.2,62.1)) [7], assuming that protection lasts for the duration that JCVI estimates 4CMenB protects infants against serogroup B meningococcal disease (i.e. 18 months after primary vaccination and 36 months after booster vaccination) [8], with vaccination costing £18/dose. Here we present additional results with vaccination costing £85/dose, and for durations of protection of 4 years and 7.5 years [9] after primary vaccination and booster vaccination (Table S4). We also plot the probability that vaccination is cost-effective (i.e. net monetary benefit is positive) at different costs per dose administered, under VoD and VaR targeting strategies and for different durations of protection, under each vaccine-sentiment scenario (Figure S3). As in the main paper, we calculate the net monetary benefit with a QALY valued at £20,000 (i.e. "NMB"); we also calculate the probability that net monetary benefit with a QALY valued at £30,000 (i.e. "$NMB_{£30k}$") is positive. Initial vaccination uptake (i.e. 1st-dose uptake at the start of the program) corresponds to $(1\text{-}h)r_1$: in the *All-willing* vaccine-sentiment scenario (where $(1\text{-}h)$=100%), r_1 is 40.8%(95%CrI:40.6,41.0) and uptake remains constant over time; and in the *Some-unwilling* scenario (where r_1=100%), $(1\text{-}h)$ is 40.8%(95%CrI:40.6,41.0) and uptake declines as vaccine-willing individuals are depleted.

In all cases, the longer the duration of protection the greater the NMB of vaccination, because more infections are averted and fewer doses of vaccine are used due to longer gaps between repeat vaccination.

At £18 per dose:
• Vaccination gains QALYs and is on average cost-saving in all scenarios examined (Table S4(a-c)), and in all scenarios $P(NMB_{£30k} > £0) \geq 98.6\%$, rising to 100% if the duration of protection is $\geq$ 4 years.
• If protection lasts for 1.5 years after primary vaccination and 3 years after booster vaccination (Table S4(a)) then, under 1-dose primary-vaccination strategies there is a very low probability of a net cost, although most likely vaccination is cost-saving. Even if there *is* a net cost to vaccination under these scenarios then the QALY gains mean that under all scenarios examined the 95%CrI of the NMB is always positive.
• If protection lasts for 4 years or more after primary and booster vaccination (Table S4(b,c)) then the 95%CrI of net savings is always positive and the 95%CrI of the NMB is always positive.
• In each table, for each vaccine-sentiment scenario each vaccination strategy saves more money and gains more QALYs than the one above and therefore dominates the one above. Therefore, if VaR targeting is feasible then the favored strategy is 2-dose primary vaccination with VaR targeting ("VaR(2d)") and if it is not feasible then the favored strategy is 2-dose primary vaccination with VoD targeting ("VoD(2d)").
• The pattern of population vaccine sentiment is an important determinant of the value of vaccination, with the NMB of VaR(2d) being ~60% greater in the *All-willing* vaccine-sentiment scenario than in the *Some-unwilling* scenario, and the NMB of VoD(2d) being ~30% greater, for all durations of vaccine protection.

At £85 per dose:
• If protection lasts for 1.5 years after primary vaccination and 3 years after booster vaccination (Table S4(a)) then vaccination has a substantial net cost, and has a negative mean net monetary benefit with a QALY valued at £20,000 (i.e. "NMB"), and the probability that net monetary benefit with a QALY valued at £30,000 is positive is no greater than 41.9% in any of the scenarios examined (i.e. $P(NMB_{£30k} > £0) \leq 41.9\%$).
• If protection lasts for 4 years after primary and booster vaccination (Table S4(b)) then VoD-targeting scenarios have a modest average net cost and VaR-targeting scenarios have a modest net saving, with QALY gains making the average NMB positive in all cases. However, in all cases a substantial proportion of the 95%CrI of the NMB is negative due to there being a substantial probability of vaccination having a considerable net cost. Furthermore, $P(NMB_{£30k} > £0) \leq 74.5\%$.
• If protection lasts for 7.5 years after primary and booster vaccination (Table S4(c)) then in all scenarios vaccination is on-average cost-saving but with a substantial probability of having a net cost, resulting in NMBs which are positive on average, but with a substantial proportion of the 95%CrI of these NMBs being negative. $P(NMB_{£30k} > £0) \leq 90\%$, i.e. failing to meet JCVI's "second criterion" for cost-effectiveness, except for VaR(2d) in the *All-willing* vaccine-sentiment scenario, where $P(NMB_{£30k} > £0) = 90.1\%$.

Table S4(a): Protection 1.5 years after primary vaccination, 3 years after booster vaccination

Vaccination strategy	Gonorrhoea cases averted, thousands		Testing & treatment costs saved, £M	QALYs gained	Vaccine doses administered, thousands		£18/dose				£85/dose			
	Undiscounted	Discounted			Undiscounted	Discounted	Vaccination costs incurred, £M	Net costs saved, £M	NMB, £M	Probability $NMB_{£30k}$ >£0	Vaccination costs incurred, £M	Net costs saved, £M	NMB, £M	Probability $NMB_{£30k}$ >£0
All willing														
VoD(1d)	32.17 (13.06,59.58)	26.46 (10.81,48.88)	3.96 (1.60,7.33)	30.94 (11.34,63.05)	106.19 (87.53,124.56)	90.31 (74.53,105.88)	1.63 (1.34,1.91)	2.34 (-0.13,5.88)	2.96 (0.08,7.10)	98.9%	7.68 (6.34,9.00)	-3.71 (-6.60,0.37)	-3.09 (-6.32,1.42)	10.1%
VoD(2d)	57.19 (28.06,91.14)	46.44 (22.95,74.16)	6.97 (3.50,11.56)	54.48 (24.46,100.47)	166.09 (134.26,196.56)	141.68 (115.02,167.11)	2.55 (2.07,3.01)	4.42 (0.75,9.29)	5.51 (1.35,11.01)	100.0%	12.04 (9.78,14.20)	-5.07 (-9.72,0.65)	-3.99 (-9.09,2.57)	14.2%
VaR(1d)	74.88 (21.04,165.15)	61.58 (17.42,135.73)	9.22 (2.66,20.33)	72.05 (19.00,168.30)	220.25 (165.45,284.95)	187.72 (140.64,243.41)	3.38 (2.53,4.38)	5.84 (-0.05,16.16)	7.28 (0.39,19.50)	99.1%	15.96 (11.95,20.69)	-6.74 (-11.14,1.22)	-5.30 (-10.39,4.40)	12.8%
VaR(2d)	139.98 (51.77,238.91)	113.68 (42.06,194.70)	17.05 (5.98,30.51)	133.23 (45.68,262.74)	323.60 (260.53,390.85)	278.32 (222.60,338.78)	5.01 (4.01,6.10)	12.04 (1.88,24.62)	14.71 (2.80,29.01)	100.0%	23.66 (18.92,28.80)	-6.61 (-14.15,3.31)	-3.94 (-12.78,7.96)	32.3%
Some unwilling														
VoD(1d)	27.57 (11.02,51.73)	22.73 (9.12,42.63)	3.40 (1.35,6.45)	26.57 (9.51,54.45)	92.00 (77.87,107.06)	78.41 (66.43,91.14)	1.41 (1.20,1.64)	1.99 (-0.15,5.14)	2.52 (0.06,6.21)	98.6%	6.66 (5.65,7.75)	-3.26 (-5.78,0.28)	-2.73 (-5.54,1.18)	9.7%
VoD(2d)	43.16 (20.25,71.74)	35.30 (16.65,58.55)	5.29 (2.52,9.08)	41.34 (17.83,78.39)	121.47 (102.67,143.24)	104.91 (88.85,123.57)	1.89 (1.60,2.22)	3.41 (0.58,7.31)	4.23 (0.97,8.78)	100.0%	8.92 (7.55,10.50)	-3.62 (-7.06,0.72)	-2.80 (-6.60,2.33)	16.1%
VaR(1d)	52.20 (16.06,111.06)	43.14 (13.35,91.89)	6.46 (2.01,14.12)	50.45 (14.41,117.27)	156.99 (126.77,187.53)	134.30 (108.26,160.76)	2.42 (1.95,2.89)	4.04 (-0.11,11.37)	5.05 (0.21,13.47)	99.0%	11.42 (9.20,13.66)	-4.96 (-8.31,1.10)	-3.95 (-7.84,3.23)	12.2%
VaR(2d)	85.16 (30.68,163.82)	69.77 (25.28,133.77)	10.46 (3.65,20.65)	81.68 (28.43,172.89)	178.30 (153.48,198.45)	156.05 (133.78,174.48)	2.81 (2.41,3.14)	7.65 (1.23,17.56)	9.28 (1.77,21.09)	100.0%	13.26 (11.37,14.83)	-2.80 (-8.42,6.39)	-1.17 (-7.72,9.71)	41.9%

Table S4(b): Protection 4 years after primary vaccination and booster vaccination

Vaccination strategy	Gonorrhoea cases averted, thousands		Testing & treatment costs saved, £M	QALYs gained	Vaccine doses administered, thousands		£18/dose				£85/dose			
	Undiscounted	Discounted			Undiscounted	Discounted	Vaccination costs incurred, £M	Net costs saved, £M	NMB, £M	Probability $NMB_{£30k}$ >£0	Vaccination costs incurred, £M	Net costs saved, £M	NMB, £M	Probability $NMB_{£30k}$ >£0
All willing														
VoD(1d)	53.52 (21.91,97.02)	43.57 (17.92,78.69)	6.53 (2.67,11.74)	51.04 (19.10,101.95)	90.38 (73.39,107.22)	77.55 (63.30,91.82)	1.40 (1.14,1.65)	5.13 (1.14,10.52)	6.16 (1.58,12.50)	100.0%	6.59 (5.38,7.80)	-0.06 (-4.53,5.99)	0.96 (-4.13,7.87)	64.5%
VoD(2d)	77.65 (39.09,121.76)	62.99 (31.79,99.00)	9.45 (4.78,15.31)	73.85 (33.64,132.79)	141.15 (111.63,170.36)	121.38 (96.52,145.73)	2.18 (1.74,2.62)	7.27 (2.36,13.37)	8.74 (3.11,15.94)	100.0%	10.32 (8.20,12.39)	-0.87 (-6.69,6.40)	0.61 (-5.89,8.76)	60.5%
VaR(1d)	110.74 (33.69,214.27)	90.40 (27.61,175.23)	13.54 (4.19,26.80)	105.79 (30.17,224.83)	167.00 (135.36,201.09)	144.29 (116.26,174.71)	2.60 (2.09,3.14)	10.95 (2.04,23.87)	13.06 (2.65,27.96)	100.0%	12.26 (9.88,14.85)	1.28 (-6.36,12.97)	3.39 (-5.60,16.70)	72.2%
VaR(2d)	160.94 (64.89,252.51)	130.93 (52.76,206.95)	19.63 (7.46,32.21)	153.28 (58.02,283.00)	260.40 (216.23,304.14)	225.84 (185.95,265.93)	4.07 (3.35,4.79)	15.57 (3.98,27.53)	18.63 (5.31,32.76)	100.0%	19.20 (15.81,22.60)	0.44 (-8.81,11.17)	3.50 (-7.57,16.49)	74.5%
Some unwilling														
VoD(1d)	42.11 (16.85,78.14)	34.46 (13.80,64.25)	5.16 (2.01,9.78)	40.32 (14.55,81.82)	71.60 (60.10,85.23)	61.78 (52.03,73.33)	1.11 (0.94,1.32)	4.05 (0.81,8.69)	4.86 (1.12,10.30)	100.0%	5.25 (4.42,6.23)	-0.09 (-3.63,4.93)	0.72 (-3.23,6.45)	63.1%
VoD(2d)	57.82 (27.43,95.24)	47.25 (22.46,77.83)	7.08 (3.32,12.14)	55.29 (24.31,103.75)	102.68 (84.99,123.83)	89.35 (74.32,107.26)	1.61 (1.34,1.93)	5.48 (1.66,10.58)	6.58 (2.19,12.49)	100.0%	7.59 (6.32,9.12)	-0.51 (-4.69,5.12)	0.60 (-4.20,7.08)	61.2%
VaR(1d)	68.23 (22.27,138.64)	56.17 (18.45,114.20)	8.41 (2.76,17.28)	65.69 (20.00,143.91)	103.40 (89.02,115.57)	90.06 (77.21,100.95)	1.62 (1.39,1.82)	6.79 (1.28,15.56)	8.10 (1.70,18.31)	100.0%	7.66 (6.56,8.58)	0.76 (-4.39,9.29)	2.07 (-4.02,12.04)	69.9%
VaR(2d)	96.62 (37.42,176.21)	79.33 (30.90,144.76)	11.89 (4.41,22.39)	92.77 (34.63,190.26)	141.56 (124.37,157.49)	124.74 (108.95,138.85)	2.25 (1.96,2.50)	9.64 (2.38,20.08)	11.50 (3.09,23.36)	100.0%	10.60 (9.26,11.80)	1.29 (-5.33,11.26)	3.14 (-4.60,14.79)	74.0%

Table S4(c): Protection 7.5 years after primary vaccination and booster vaccination

Vaccination strategy	Gonorrhoea cases averted, thousands		Testing & treatment costs saved, £M	QALYs gained	Vaccine doses administered, thousands		£18/dose				£85/dose			
	Undiscounted	Discounted			Undiscounted	Discounted	Vaccination costs incurred, £M	Net costs saved, £M	NMB, £M	Probability $NMB_{£30k}$ >£0	Vaccination costs incurred, £M	Net costs saved, £M	NMB, £M	Probability $NMB_{£30k}$ >£0
All willing														
VoD(1d)	65.49<br>(27.04,116.63)	53.03<br>(22.02,94.08)	7.95<br>(3.25,14.03)	62.21<br>(23.60,123.19)	82.00<br>(65.15,98.68)	70.87<br>(56.85,85.02)	1.28<br>(1.02,1.53)	6.68<br>(1.85,12.94)	7.92<br>(2.35,15.21)	100.0%	6.02<br>(4.83,7.23)	1.93<br>(-3.35,8.99)	3.17<br>(-2.94,11.37)	83.8%
VoD(2d)	90.73<br>(46.23,140.46)	73.36<br>(37.40,113.64)	11.01<br>(5.63,17.58)	86.07<br>(39.79,153.00)	126.22<br>(98.06,154.63)	109.44<br>(85.81,133.21)	1.97<br>(1.54,2.40)	9.04<br>(3.41,15.88)	10.76<br>(4.30,18.70)	100.0%	9.30<br>(7.29,11.32)	1.71<br>(-4.81,9.73)	3.43<br>(-3.92,12.30)	81.5%
VaR(1d)	127.02<br>(40.99,230.37)	103.38<br>(33.41,188.82)	15.50<br>(5.01,28.96)	121.03<br>(36.41,244.47)	141.07<br>(119.13,162.87)	123.34<br>(103.25,143.72)	2.22<br>(1.86,2.59)	13.28<br>(3.07,26.41)	15.70<br>(3.84,30.61)	100.0%	10.48<br>(8.78,12.22)	5.01<br>(-4.30,17.53)	7.43<br>(-3.46,21.87)	88.6%
VaR(2d)	172.75<br>(73.86,257.47)	140.47<br>(59.91,211.10)	21.06<br>(8.51,33.02)	164.45<br>(66.07,295.17)	219.17<br>(188.90,247.50)	192.43<br>(164.24,219.67)	3.46<br>(2.96,3.95)	17.60<br>(5.51,29.32)	20.89<br>(7.03,34.42)	100.0%	16.36<br>(13.96,18.67)	4.71<br>(-5.78,15.45)	8.00<br>(-4.27,20.95)	90.1%
Some unwilling														
VoD(1d)	49.57<br>(19.77,92.01)	40.41<br>(16.13,75.17)	6.06<br>(2.37,11.42)	47.33<br>(17.02,95.63)	61.68<br>(51.52,74.60)	53.78<br>(45.13,64.68)	0.97<br>(0.81,1.16)	5.09<br>(1.32,10.46)	6.03<br>(1.71,12.37)	100.0%	4.57<br>(3.84,5.50)	1.49<br>(-2.60,7.19)	2.43<br>(-2.19,8.99)	83.2%
VoD(2d)	67.37<br>(32.02,110.59)	54.88<br>(26.25,90.17)	8.23<br>(3.84,14.06)	64.26<br>(28.28,119.71)	90.97<br>(74.66,111.65)	79.87<br>(66.14,97.38)	1.44<br>(1.19,1.75)	6.79<br>(2.35,12.65)	8.08<br>(2.97,14.67)	100.0%	6.79<br>(5.62,8.28)	1.44<br>(-3.31,7.91)	2.73<br>(-2.73,9.91)	82.0%
VaR(1d)	74.81<br>(24.90,148.35)	61.49<br>(20.58,121.89)	9.21<br>(3.10,18.48)	71.92<br>(22.26,155.62)	81.42<br>(71.42,91.38)	72.01<br>(63.03,80.53)	1.30<br>(1.13,1.45)	7.91<br>(1.89,17.17)	9.35<br>(2.34,20.28)	100.0%	6.12<br>(5.36,6.85)	3.09<br>(-2.91,12.28)	4.53<br>(-2.40,15.33)	87.8%
VaR(2d)	104.11<br>(42.02,183.84)	85.41<br>(34.62,150.64)	12.80<br>(4.89,23.53)	99.87<br>(38.55,199.28)	116.73<br>(103.70,132.11)	104.27<br>(92.48,117.31)	1.88<br>(1.66,2.11)	10.93<br>(3.13,21.69)	12.92<br>(3.96,24.90)	100.0%	8.86<br>(7.86,9.97)	3.94<br>(-3.41,14.66)	5.94<br>(-2.60,17.79)	89.6%

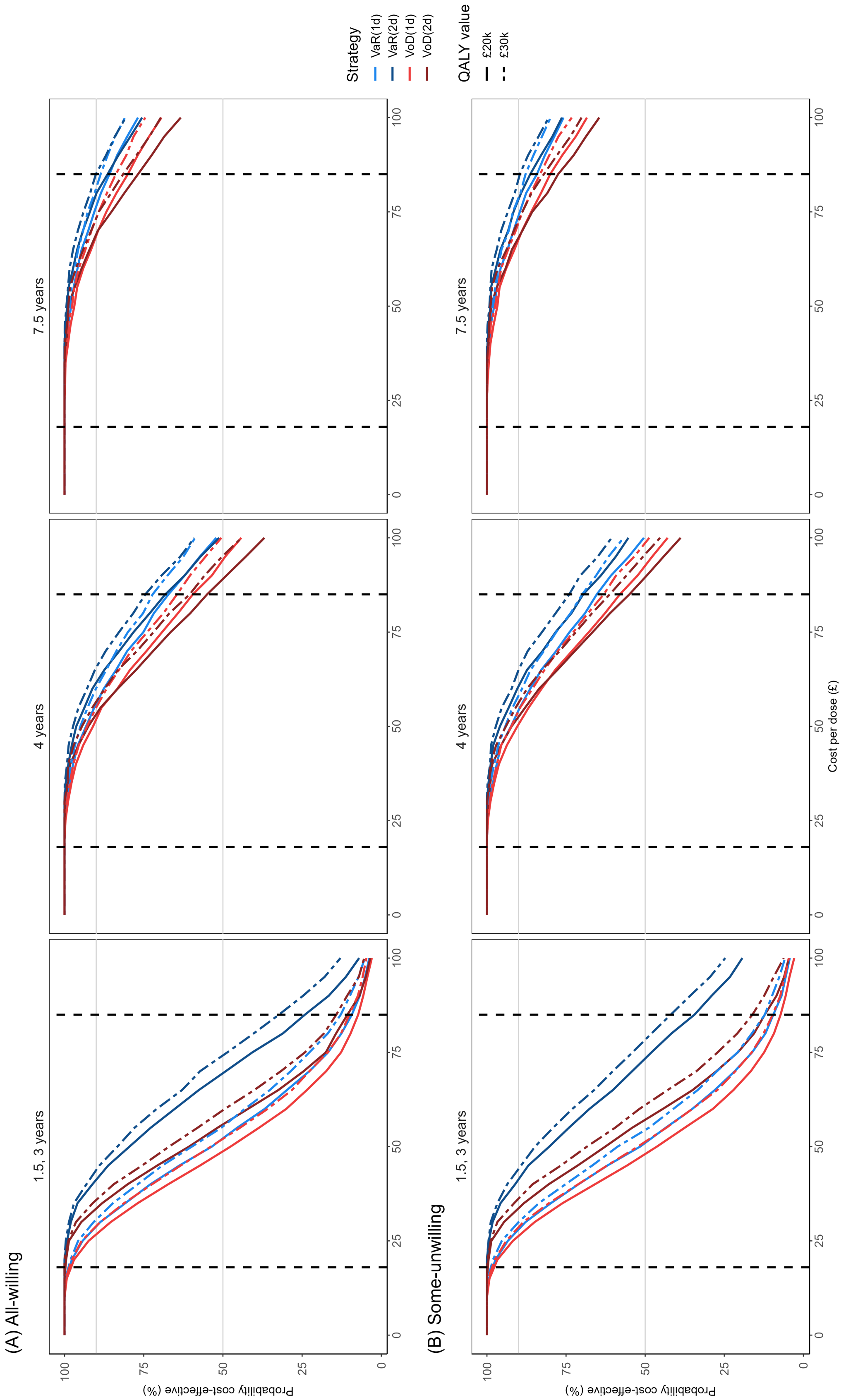

Figure S3: Probability that vaccination is cost-effective (i.e. net monetary benefit is positive) at different costs per dose administered, under different vaccination strategies targeting higher-risk individuals, and for different durations of protection, under each vaccine-sentiment scenario, (A) *All-willing* and (B) *Some-unwilling*. Solid and dashed lines show calculations with a QALY valued at £20,000 and £30,000, respectively; to be considered cost-effective a strategy's solid line should be $\geq 50\%$ and its dashed line should be $\geq 90\%$. Vertical dashed lines show costs per dose administered of £18 and £85.

2.2.1 Cost-effectiveness acceptability curves (Figure S4)

Here we present cost-effectiveness acceptability curves, showing the probability that each strategy is cost-effective at different willingness-to-pay thresholds (Figure S4).

At £18 per dose, all vaccination strategies have a very high probability of being cost-effective at almost all levels of willingness-to-pay, even in the shortest duration of protection scenario.

At £85 per dose:

• If the duration of protection is 18 months after primary vaccination and 3 years after booster vaccination then for vaccination to be likely to be considered cost-effective requires a high willingness-to-pay, with VaR(2-dose) having a substantially higher probability than other strategies.

• If the duration of protection after primary and booster vaccination is 4 years or 7.5 years then all vaccination strategies have >50% probability of being considered cost-effective at £20,000/QALY but to have a >90% probability at £30,000/QALY requires VaR targeting and 7.5 years' protection.

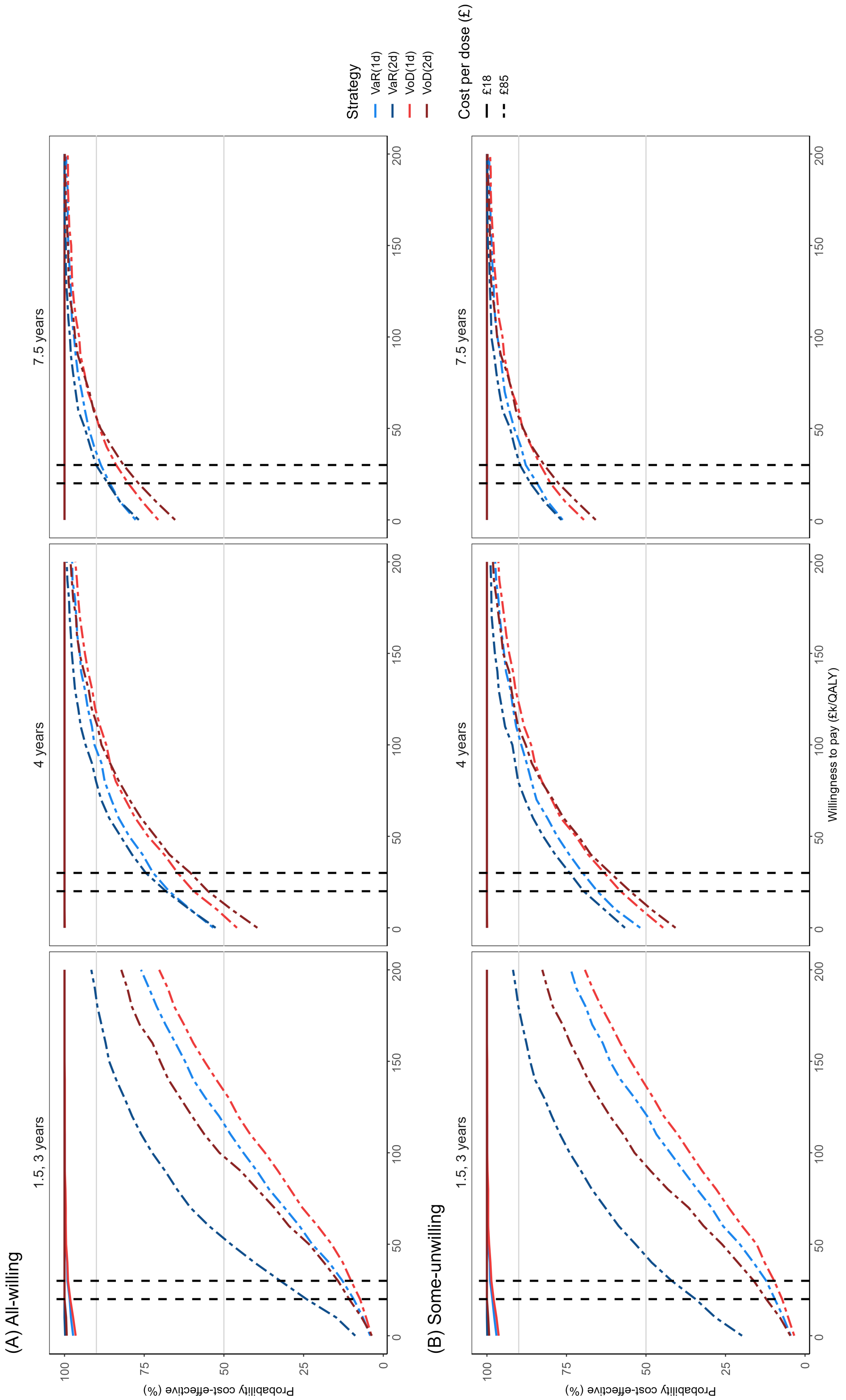

Figure S4: Cost-effectiveness acceptability curves, showing the probability that vaccination is cost-effective at different willingness-to-pay thresholds (i.e. different £ values assigned to a QALY), under different vaccination strategies targeting higher-risk individuals, and for different durations of protection, under each vaccine-sentiment scenario, (A) *All-willing* and (B) *Some-unwilling*. Solid and dashed lines show calculations with the cost per dose administered being £18 and £85, respectively. Vertical dashed lines show a QALY valued at £20,000 and £30,000.

2.3 Health-economic analysis of vaccination of MSM in England over 10 years, offering Vaccination-on-Attendance (VoA) under different patterns of population vaccine sentiment, with different durations of vaccine protection, and costs per dose (Table S5)

Here we present results for the Vaccination-on-Attendance targeting strategy, where initial uptake of vaccination (i.e. initial first-dose uptake at the start of the program) is the same as for HPV vaccination of MSM (i.e. 1st dose 40.8%(95%CrI:40.6,41.0), 2nd dose 61.7%(61.2,62.1)) [7], with vaccination costing £18/dose and £85/dose, for different durations of protection (Table S5).

At £18 per dose:

• If protection lasts for 1.5 years after primary vaccination and 3 years after booster vaccination then the costs of vaccination exceed the cost savings from the reduction in gonorrhea testing and treatment, and therefore the vaccination program has a net cost. Under 1-dose strategies NMB is negative so vaccination is not cost-effective. Under 2-dose strategies NMB has a small positive value but with a large proportion of the 95%CrI being negative, and the probability that net monetary benefit with a QALY valued at £30,000 is positive is no greater than 56.5% in any of the scenarios examined (i.e. $P(NMB_{£30k} > £0) \leq 56.5\%$), and therefore vaccination is not cost-effective.

• If protection lasts for 4 years after primary and booster vaccination then on average vaccination is cost-saving and NMB is positive, but with a substantial proportion of the 95%CrI of net savings and NMB being negative; as $P(NMB_{£30k} > £0) \leq 80.3\%$) vaccination is not cost-effective.

• If protection lasts for 7.5 years after primary and booster vaccination then on average vaccination is cost-saving and NMB is positive, although the 95%CrI of net savings and NMB both span zero. $P(NMB_{£30k} > £0) \leq 90\%$, so vaccination is not cost-effective, except for VoA(1d) in the *All-willing* vaccine-sentiment scenario, where $P(NMB_{£30k} > £0) = 90.7\%$. However, if protection lasts for 7.5 years and the applicable vaccine-sentiment scenario is *All-willing* then VoA(1d) is inferior to VoD(2d), VaR(1d), and VaR(2d) (Table S4(c)), which all have greater NMB and greater $P(NMB_{£30k} > £0)$ (being 100% in all cases).

At £85 per dose, vaccination with VoA targeting is not cost-effective: NMB is always negative and $P(NMB_{£30k} > £0) \leq 4.1\%$).

In summary, VoA targeting is not cost-effective even at £18/dose administered.

Table S5: Health-economic analysis of offering Vaccination-on-Attendance (VoA) with protection lasting 1.5 years after primary vaccination and 3 years after booster vaccination, or 4 years after primary vaccination and booster vaccination, or 7.5 years after primary vaccination and booster vaccination

Durations of protection 1.5, 3 years

Vaccination strategy	Gonorrhoea cases averted, thousands		Testing & treatment costs saved, £M	QALYs gained	Vaccine doses administered, thousands		£18/dose				£85/dose			
	Undiscounted	Discounted			Undiscounted	Discounted	Vaccination costs incurred, £M	Net costs saved, £M	NMB, £M	Probability $NMB_{£30k}$ >£0	Vaccination costs incurred, £M	Net costs saved, £M	NMB, £M	Probability $NMB_{£30k}$ >£0
All willing														
VoA(1d)	75.71 (21.82,165.50)	62.27 (18.12,136.51)	9.33 (2.74,20.37)	73.02 (20.05,168.60)	785.12 (699.32,865.87)	667.08 (593.95,735.75)	12.01 (10.69,13.24)	-2.68 (-9.49,9.05)	-1.22 (-9.01,12.26)	39.7%	56.70 (50.49,62.54)	-47.37 (-56.68,-32.47)	-45.91 (-56.26,-29.45)	0.0%
VoA(2d)	141.06 (53.33,239.38)	114.57 (43.32,195.34)	17.19 (6.10,30.55)	134.48 (47.73,263.62)	1261.99 (1112.11,1393.28)	1074.33 (948.65,1186.71)	19.34 (17.08,21.36)	-2.14 (-13.58,12.21)	0.55 (-12.43,16.89)	56.5%	91.32 (80.64,100.87)	-74.12 (-89.46,-52.90)	-71.43 (-88.32,-47.86)	0.0%
Some unwilling														
VoA(1d)	52.95 (16.44,111.70)	43.76 (13.69,92.24)	6.56 (2.06,14.25)	51.33 (15.00,117.66)	623.21 (557.36,679.17)	531.24 (475.29,579.11)	9.56 (8.56,10.42)	-3.00 (-7.76,5.32)	-1.98 (-7.42,7.53)	29.7%	45.16 (40.40,49.22)	-38.60 (-45.49,-27.34)	-37.57 (-45.11,-25.30)	0.0%
VoA(2d)	84.08 (30.53,161.42)	68.89 (25.08,132.78)	10.33 (3.61,20.46)	80.83 (27.54,171.05)	759.04 (119.60,849.01)	657.38 (103.33,736.57)	11.83 (1.86,13.26)	-1.50 (-8.78,8.69)	0.12 (-8.14,12.09)	51.9%	55.88 (8.78,62.61)	-45.54 (-56.50,-3.66)	-43.93 (-55.86,-2.88)	0.8%

Duration of protection 4 years

Vaccination strategy	Gonorrhoea cases averted, thousands		Testing & treatment costs saved, £M	QALYs gained	Vaccine doses administered, thousands		£18/dose				£85/dose			
	Undiscounted	Discounted			Undiscounted	Discounted	Vaccination costs incurred, £M	Net costs saved, £M	NMB, £M	Probability $NMB_{£30k}$ >£0	Vaccination costs incurred, £M	Net costs saved, £M	NMB, £M	Probability $NMB_{£30k}$ >£0
All willing														
VoA(1d)	111.91 (34.91,214.88)	91.37 (28.66,175.64)	13.70 (4.31,26.93)	107.15 (32.01,225.28)	661.36 (588.78,723.56)	566.71 (505.03,620.43)	10.20 (9.09,11.17)	3.50 (-5.97,17.13)	5.64 (-5.36,21.49)	80.3%	48.17 (42.93,52.74)	-34.47 (-45.84,-17.97)	-32.33 (-45.05,-13.54)	0.1%
VoA(2d)	162.20 (66.70,252.69)	131.98 (54.22,207.46)	19.80 (7.78,32.28)	154.74 (60.35,284.39)	1081.90 (954.98,1184.35)	926.83 (819.52,1015.73)	16.68 (14.75,18.28)	3.12 (-9.42,16.65)	6.21 (-8.12,21.85)	80.3%	78.78 (69.66,86.34)	-58.98 (-73.79,-39.52)	-55.88 (-72.51,-35.23)	0.0%
Some unwilling														
VoA(1d)	69.31 (23.13,139.26)	57.06 (19.11,114.67)	8.56 (2.86,17.44)	66.96 (20.57,146.61)	460.06 (414.45,491.91)	397.77 (357.92,425.86)	7.16 (6.44,7.67)	1.40 (-4.39,10.66)	2.74 (-3.97,13.42)	74.1%	33.81 (30.42,36.20)	-25.25 (-32.23,-13.88)	-23.91 (-31.74,-11.22)	0.0%
VoA(2d)	95.98 (37.74,176.00)	78.80 (31.14,144.12)	11.82 (4.47,22.36)	92.39 (33.34,189.15)	629.69 (102.03,697.18)	549.49 (88.56,609.58)	9.89 (1.59,10.97)	1.93 (-5.96,12.37)	3.78 (-5.07,15.71)	76.9%	46.71 (7.53,51.81)	-34.89 (-45.34,-0.78)	-33.04 (-44.65,0.32)	3.1%

Duration of protection 7.5 years

Vaccination strategy	Gonorrhoea cases averted, thousands		Testing & treatment costs saved, £M	QALYs gained	Vaccine doses administered, thousands		£18/dose				£85/dose			
	Undiscounted	Discounted			Undiscounted	Discounted	Vaccination costs incurred, £M	Net costs saved, £M	NMB, £M	Probability $NMB_{£30k}$ >£0	Vaccination costs incurred, £M	Net costs saved, £M	NMB, £M	Probability $NMB_{£30k}$ >£0
All willing														
VoA(1d)	128.31 (42.07,230.65)	104.45 (34.31,189.05)	15.67 (5.18,29.03)	122.52 (38.52,245.71)	594.67 (527.62,646.38)	513.29 (455.94,558.60)	9.24 (8.21,10.05)	6.43 (-4.09,20.40)	8.88 (-3.28,24.90)	90.7%	43.63 (38.75,47.48)	-27.96 (-40.19,-11.87)	-25.51 (-39.40,-7.19)	0.7%
VoA(2d)	174.07 (76.26,258.09)	141.57 (61.99,211.66)	21.24 (8.83,33.15)	165.96 (68.74,296.77)	963.74 (851.35,1047.75)	832.07 (735.84,904.77)	14.98 (13.25,16.29)	6.26 (-6.66,19.12)	9.58 (-5.04,24.81)	89.9%	70.73 (62.55,76.91)	-49.49 (-64.39,-31.54)	-46.17 (-62.74,-26.76)	0.0%
Some unwilling														
VoA(1d)	76.04 (26.25,149.06)	62.51 (21.64,122.90)	9.37 (3.22,18.59)	73.36 (22.96,157.32)	382.43 (348.98,404.23)	334.86 (304.85,354.71)	6.03 (5.49,6.38)	3.35 (-2.85,12.82)	4.81 (-2.38,15.97)	87.5%	28.46 (25.91,30.15)	-19.09 (-26.14,-8.23)	-17.62 (-25.62,-5.12)	0.8%
VoA(2d)	103.73 (42.22,184.55)	85.09 (34.77,151.05)	12.76 (5.01,23.57)	99.76 (37.67,198.21)	543.63 (90.33,596.19)	479.55 (79.29,527.30)	8.63 (1.43,9.49)	4.13 (-4.10,14.82)	6.13 (-3.16,18.17)	88.5%	40.76 (6.74,44.82)	-28.00 (-38.27,1.01)	-26.00 (-37.43,2.37)	4.1%

2.4 Health-economic value of vaccination against gonorrhea of MSM in England over 10 years under different strategies targeting higher-risk individuals, comparing different durations of vaccine protection and population vaccine-sentiment scenarios (Figures S5-S8)

In the main paper, Figures 3 & 4 present heatmaps showing the mean NMB of the VaR and VoD targeting strategies in the two vaccine-sentiment scenarios for different levels of uptake of the 1st and 2nd primary vaccination doses, with vaccination costing £18/dose administered. Below, we supplement those heatmaps with line graphs, which are transects through the heatmaps with the NMB value represented on the vertical axis rather than by color. Initial vaccination uptake (i.e. 1st-dose uptake at the start of the program) corresponds to (1-h)r_1: in the *All-willing* vaccine-sentiment scenario (where (1-h)=100%), r_1 is varied; and in the *Some-unwilling* scenario (where r_1=100%), (1-h) is varied.

In the figures below, Panel (A) reproduces the heatmap from main paper Figure 3A, 3B, 4A, or 4B. Panel (B) shows the relationship between mean NMB and initial vaccination uptake for different values of 2nd-dose uptake (with zero representing a one-dose primary vaccination schedule). Panel (C) shows the relationship between mean NMB and 2nd-dose uptake for different values of initial vaccination uptake. Simulations compare each vaccination strategy against no vaccination, using 1000 sets of sampled epidemiological and health-economic parameters.

Note how in the *All-willing* vaccine-sentiment scenario (Figures S5B and S7B), as initial vaccination uptake increases from zero, NMB initially increases steeply before latterly showing diminishing returns, particularly under VaR targeting (Figure S5B). In contrast, in the *Some-unwilling* scenario the relationship between NMB and initial vaccination uptake (Figures S6B and S8B) is much more linear. The relationship between 2nd-dose uptake and NMB is approximately linear in all cases (Figures S5C, S6C, S7C, and S8C).

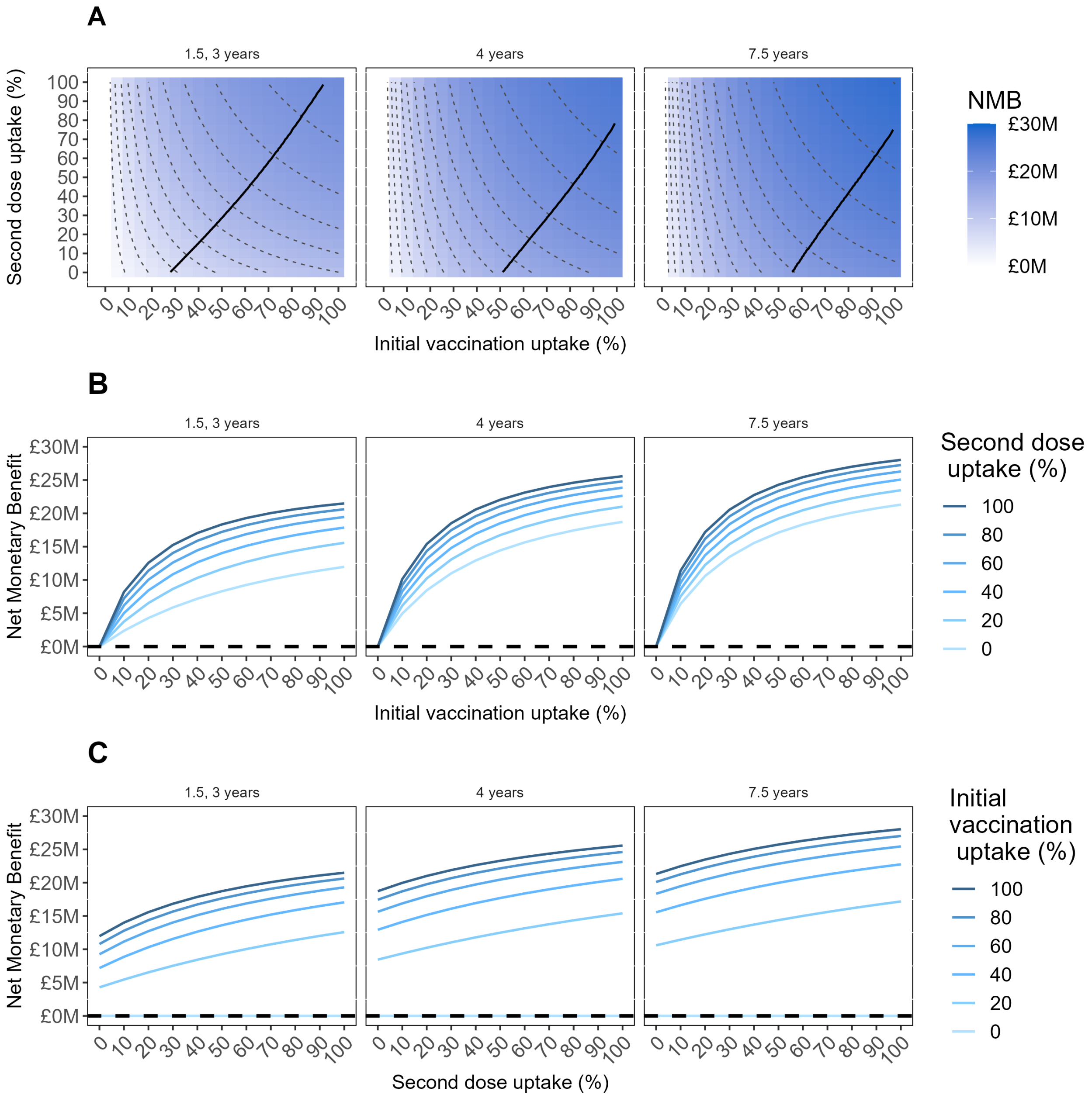

Figure S5: VaR; *All-willing*. (A) Reproduction of Figure 3A in the main paper. (B) Relationship between mean NMB and initial vaccination uptake for different values of 2nd-dose uptake (with zero representing a one-dose primary vaccination schedule). (C) Relationship between mean NMB and 2nd-dose uptake for different values of initial vaccination uptake. Simulations compare each vaccination strategy against no vaccination, using 1000 sets of sampled epidemiological and health-economic parameters.

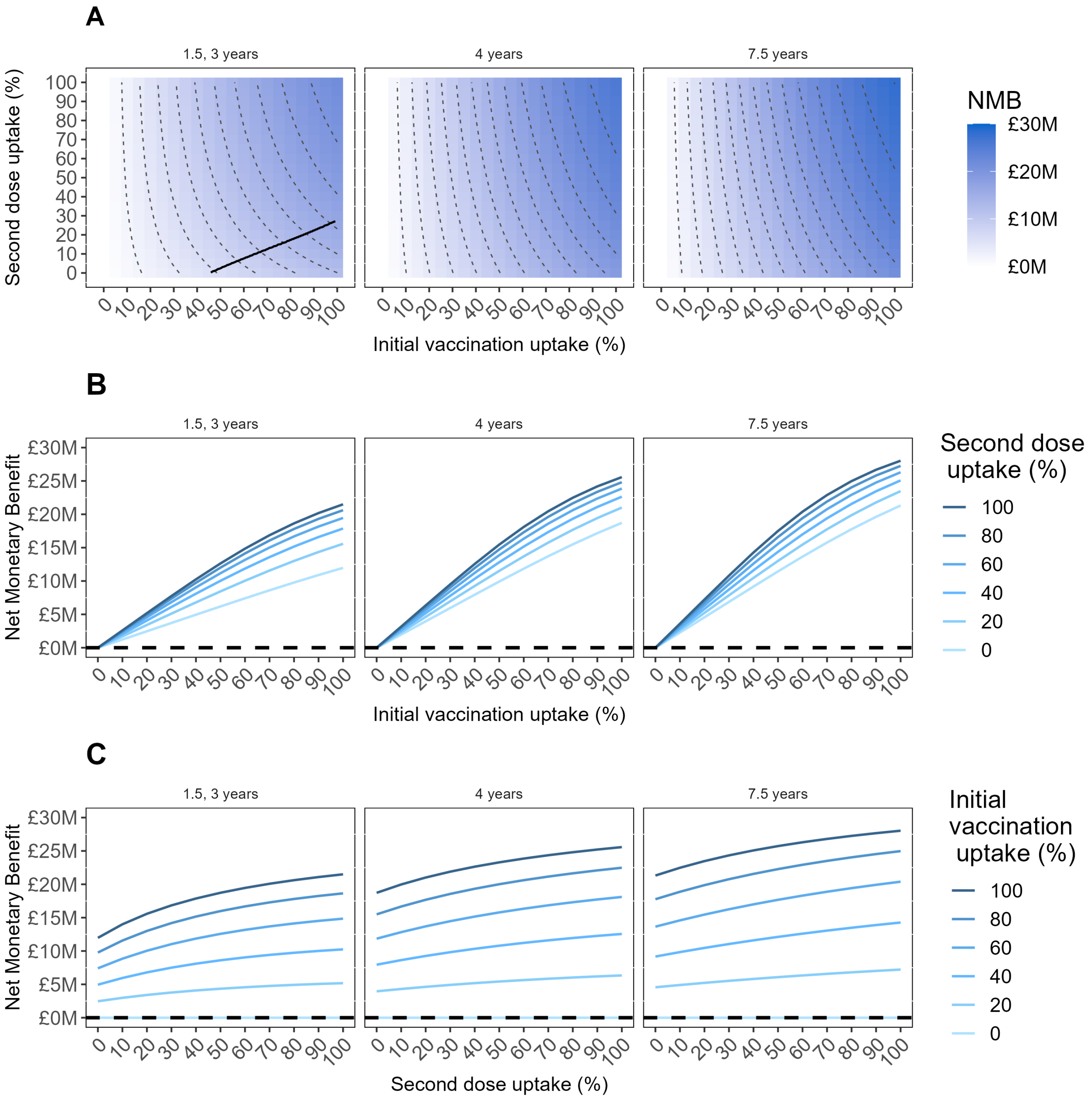

Figure S6: VaR; *Some-unwilling*. (A) Reproduction of Figure 3B in the main paper. (B) Relationship between mean NMB and initial vaccination uptake for different values of 2nd-dose uptake (with zero representing a one-dose primary vaccination schedule). (C) Relationship between mean NMB and 2nd-dose uptake for different values of initial vaccination uptake. Simulations compare each vaccination strategy against no vaccination, using 1000 sets of sampled epidemiological and health-economic parameters.

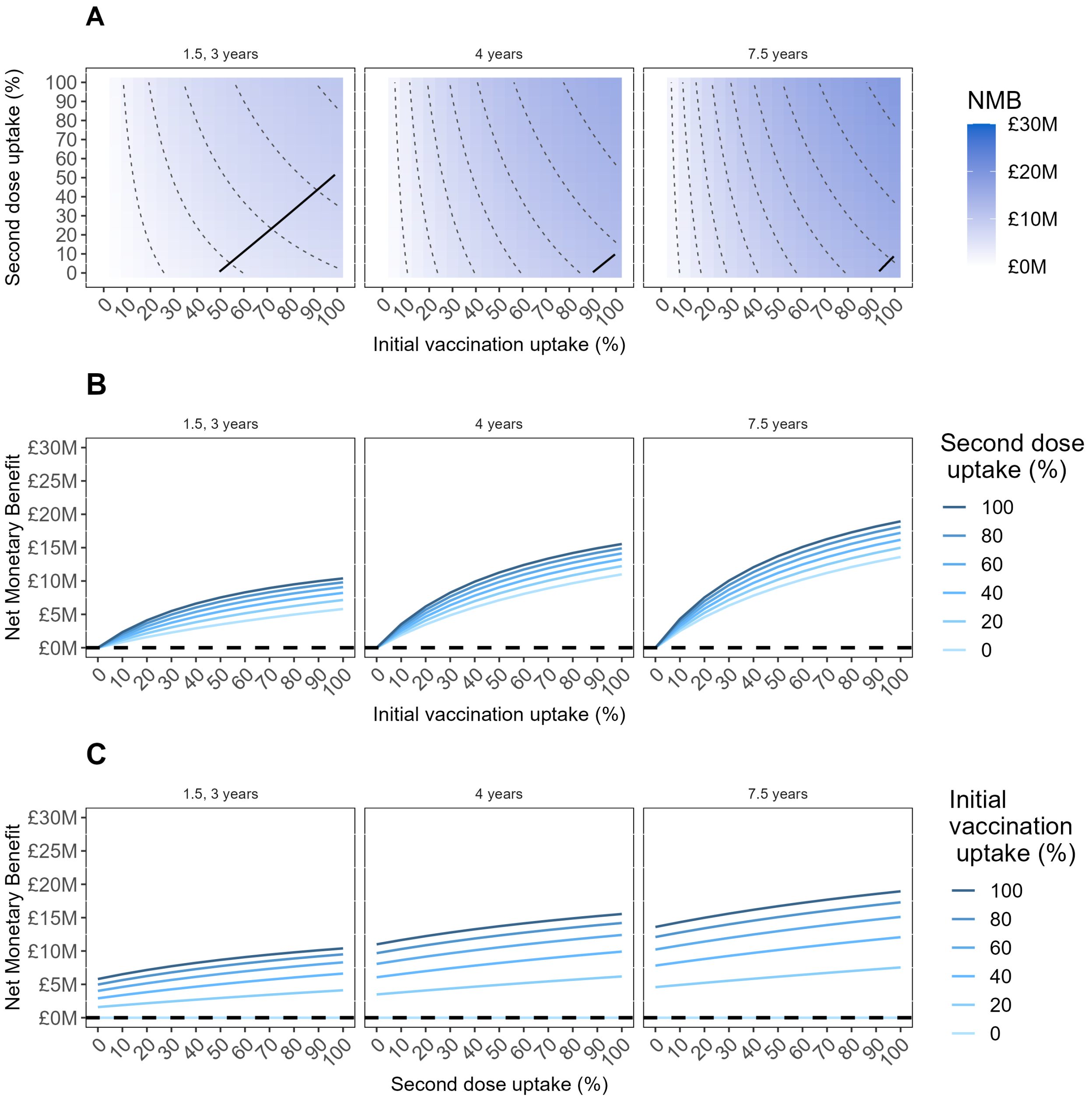

Figure S7: VoD; *All-willing*. (A) Reproduction of Figure 4A in the main paper. (B) Relationship between mean NMB and initial vaccination uptake for different values of 2nd-dose uptake (with zero representing a one-dose primary vaccination schedule). (C) Relationship between mean NMB and 2nd-dose uptake for different values of initial vaccination uptake. Simulations compare each vaccination strategy against no vaccination, using 1000 sets of sampled epidemiological and health-economic parameters.

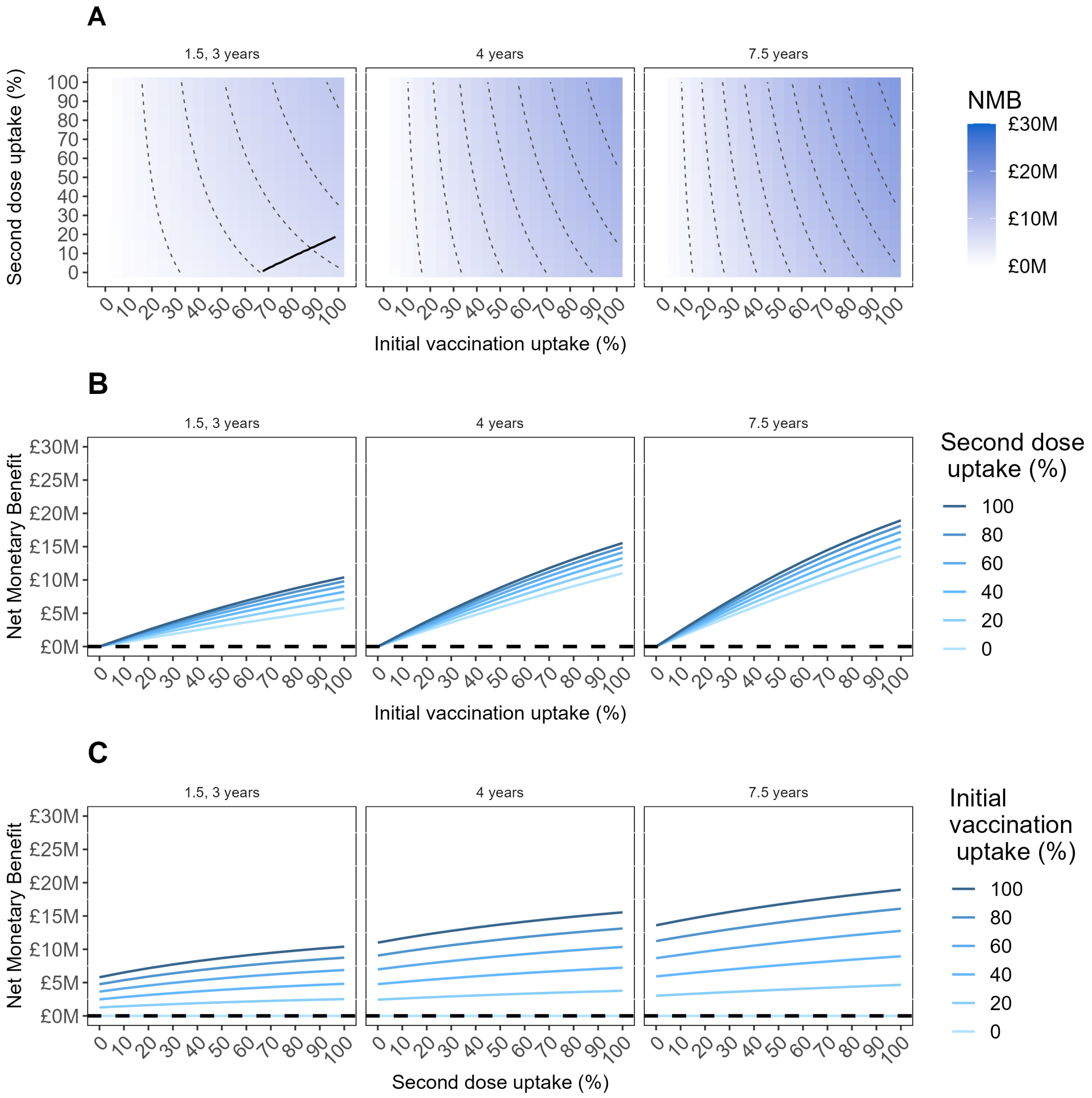

Figure S8: VoD; *Some-unwilling*. (A) Reproduction of Figure 4B in the main paper. (B) Relationship between mean NMB and initial vaccination uptake for different values of 2nd-dose uptake (with zero representing a one-dose primary vaccination schedule). (C) Relationship between mean NMB and 2nd-dose uptake for different values of initial vaccination uptake. Simulations compare each vaccination strategy against no vaccination, using 1000 sets of sampled epidemiological and health-economic parameters.

2.5 Health-economic value of vaccination for pairwise combinations of 1st and 2nd dose uptake under different strategies targeting higher-risk individuals, population vaccine-sentiment scenarios, durations of vaccine protection, and costs per dose (Table S6)

The value of promotional activity to increase 1st- or 2nd-dose uptake is measured by the increase in NMB that it achieves. This depends on the size of the increase in 1st- or 2nd-dose uptake resulting from the promotional activity, and on the values of 1st- and 2nd-dose uptake without that promotional activity. To enable readers to calculate the increase in NMB, Table S6 on the following pages reports the mean NMB (and 95%CrI) in £M for different levels of uptake of 1st and 2nd doses under different scenarios, (a)-(x), as described in the table on this page. Initial vaccination uptake (i.e. 1st-dose uptake at the start of the program) corresponds to $(1\text{-}h)r_1$: in the *All-willing* vaccine-sentiment scenario (where $(1\text{-}h)$=100%), r_1 is varied; and in the *Some-unwilling* scenario (where r_1=100%), $(1\text{-}h)$ is varied.

At £18 per dose:
- In all cases the mean and 95%CrI of the NMB is positive.
- In all cases $P(\text{NMB}_{\pounds 30\text{k}} > \pounds 0) > 90\%$.

At £85 per dose:
- The mean NMB is negative if protection lasts 1.5 years after primary vaccination and 3 years after booster vaccination; almost always positive if protection lasts 4 years; and always positive if protection lasts 7.5 years.
- In all cases the 95%CrI of the NMB spans zero.
- In all cases $P(\text{NMB}_{\pounds 30\text{k}} > \pounds 0) < 90\%$, except scenario (o), where protection lasts 7.5 years, the vaccine-sentiment scenario is *All-willing*, VaR targeting is used, and

(i) 1st-dose uptake ≥ 80% (with any level of 2nd-dose uptake) or
(ii) 1st-dose uptake ≥ 50% and 2nd-dose uptake ≥ 10% or
(iii) 1st-dose uptake ≥ 40% and 2nd-dose uptake is 50%-60%.

Cost per dose	Targeting strategy	Vaccine sentiment	Duration(s) of protection (years)	Scenario
£18	VaR	All-willing	1.5, 3	a
			4	b
			7.5	c
		Some-unwilling	1.5, 3	d
			4	e
			7.5	f
	VoD	All-willing	1.5, 3	g
			4	h
			7.5	i
		Some-unwilling	1.5, 3	j
			4	k
			7.5	l
£85	VaR	All-willing	1.5, 3	m
			4	n
			7.5	o
		Some-unwilling	1.5, 3	p
			4	q
			7.5	r
	VoD	All-willing	1.5, 3	s
			4	t
			7.5	u
		Some-unwilling	1.5, 3	v
			4	w
			7.5	x

Table S6: NMB for pairwise combinations of initial vaccination uptake (i.e. 1st-dose uptake at the start of the program) and 2nd dose uptake for different costs per dose, targeting strategy, and duration(s) of protection. Red numbers indicate where $P(NMB_{£30k} > £0) < 90\%$, i.e. failing to meet JCVI's "second criterion" for cost-effectiveness.

(a) £18/dose; VaR; All-willing; 1.5, 3 years

Uptake of 2nd dose (%) \ Initial vaccination uptake (%)	10	20	30	40	50	60	70	80	90	100
100	8.19 (1.22,20.37)	12.58 (2.24,26.60)	15.25 (3.09,29.54)	17.05 (3.78,31.32)	18.34 (4.34,32.80)	19.30 (4.80,33.86)	20.04 (5.15,34.72)	20.63 (5.46,35.36)	21.11 (5.72,35.89)	21.50 (5.94,36.49)
90	7.72 (1.13,19.49)	12.01 (2.10,25.92)	14.68 (2.89,29.06)	16.49 (3.52,30.73)	17.81 (4.06,32.17)	18.80 (4.52,33.19)	19.57 (4.89,34.06)	20.18 (5.17,34.73)	20.68 (5.43,35.28)	21.09 (5.65,35.91)
80	7.23 (1.05,18.52)	11.40 (1.96,25.24)	14.05 (2.73,28.47)	15.88 (3.35,30.23)	17.22 (3.86,31.53)	18.23 (4.28,32.76)	19.03 (4.62,33.73)	19.66 (4.90,34.43)	20.19 (5.13,34.76)	20.62 (5.33,35.35)
70	6.71 (0.93,17.37)	10.74 (1.75,24.43)	13.37 (2.45,27.65)	15.20 (3.06,29.34)	16.56 (3.57,30.96)	17.60 (3.99,32.14)	18.42 (4.32,33.12)	19.08 (4.61,33.79)	19.63 (4.84,34.41)	20.08 (5.04,34.85)
60	6.17 (0.83,16.02)	10.03 (1.55,23.47)	12.61 (2.18,26.60)	14.45 (2.72,28.75)	15.82 (3.18,30.42)	16.88 (3.58,31.38)	17.73 (3.93,32.26)	18.41 (4.23,33.18)	18.98 (4.50,33.78)	19.46 (4.72,34.19)
50	5.61 (0.77,14.49)	9.26 (1.44,22.12)	11.78 (2.01,25.74)	13.60 (2.51,27.66)	14.99 (2.93,29.36)	16.07 (3.29,30.96)	16.93 (3.60,31.62)	17.64 (3.87,32.13)	18.23 (4.10,32.70)	18.73 (4.30,33.30)
40	5.02 (0.68,13.08)	8.43 (1.28,20.24)	10.85 (1.81,24.54)	12.65 (2.26,26.61)	14.03 (2.66,28.44)	15.12 (3.00,29.51)	16.01 (3.29,30.57)	16.74 (3.55,31.37)	17.35 (3.78,32.08)	17.87 (3.98,32.48)
30	4.40 (0.55,11.70)	7.52 (1.05,18.58)	9.82 (1.49,22.72)	11.56 (1.87,25.33)	12.93 (2.22,27.20)	14.02 (2.53,28.28)	14.92 (2.81,29.38)	15.67 (3.07,30.36)	16.30 (3.30,30.76)	16.84 (3.50,31.20)
20	3.75 (0.45,10.19)	6.53 (0.85,16.32)	8.65 (1.21,20.60)	10.31 (1.53,23.39)	11.64 (1.83,25.28)	12.72 (2.10,26.89)	13.62 (2.35,27.87)	14.38 (2.57,28.68)	15.02 (2.77,29.27)	15.58 (2.96,29.82)
10	3.07 (0.29,8.68)	5.46 (0.56,14.41)	7.34 (0.80,18.67)	8.87 (1.03,21.80)	10.12 (1.22,23.88)	11.16 (1.40,25.24)	12.04 (1.54,26.18)	12.79 (1.68,27.32)	13.44 (1.81,28.05)	14.00 (1.92,28.87)
0	2.37 (0.11,7.14)	4.29 (0.21,12.44)	5.87 (0.30,16.33)	7.19 (0.38,19.21)	8.30 (0.45,21.74)	9.25 (0.51,23.35)	10.08 (0.57,24.97)	10.79 (0.61,26.14)	11.42 (0.65,26.91)	11.97 (0.68,27.46)

(b) £18/dose; VaR; All-willing; 4 years

Uptake of 2nd dose (%) \ Initial vaccination uptake (%)	10	20	30	40	50	60	70	80	90	100
100	10.13 (2.09,23.00)	15.40 (3.77,29.57)	18.52 (5.08,32.46)	20.59 (6.15,34.70)	22.04 (7.01,36.27)	23.12 (7.71,37.53)	23.96 (8.22,38.24)	24.61 (8.63,39.02)	25.14 (8.96,39.50)	25.58 (9.24,39.91)
90	9.69 (2.05,22.26)	14.89 (3.71,29.09)	18.02 (4.98,31.93)	20.10 (5.97,33.99)	21.58 (6.74,35.62)	22.69 (7.35,36.89)	23.54 (7.89,37.82)	24.21 (8.33,38.33)	24.76 (8.72,39.12)	25.21 (9.01,39.59)
80	9.24 (1.91,21.46)	14.34 (3.47,28.42)	17.48 (4.74,31.56)	19.58 (5.76,33.66)	21.08 (6.58,35.26)	22.21 (7.21,36.37)	23.08 (7.72,37.26)	23.78 (8.13,37.92)	24.34 (8.48,38.41)	24.81 (8.77,39.03)
70	8.77 (1.80,20.55)	13.77 (3.24,27.65)	16.89 (4.42,31.15)	19.01 (5.38,33.09)	20.54 (6.17,34.63)	21.69 (6.82,35.83)	22.58 (7.36,36.70)	23.29 (7.80,37.72)	23.88 (8.19,38.05)	24.36 (8.51,38.41)
60	8.28 (1.78,19.41)	13.15 (3.20,26.96)	16.25 (4.34,30.31)	18.38 (5.26,32.65)	19.93 (6.00,33.89)	21.11 (6.60,35.18)	22.02 (7.10,36.25)	22.76 (7.52,37.05)	23.36 (7.90,37.68)	23.86 (8.22,38.07)
50	7.78 (1.62,18.07)	12.49 (2.98,26.20)	15.56 (4.11,29.67)	17.70 (5.04,31.91)	19.26 (5.77,33.47)	20.46 (6.37,34.38)	21.40 (6.87,35.39)	22.15 (7.29,36.17)	22.77 (7.64,36.79)	23.29 (7.94,37.45)
40	7.25 (1.49,17.16)	11.79 (2.73,24.83)	14.81 (3.73,28.95)	16.94 (4.59,31.30)	18.52 (5.32,32.61)	19.73 (5.94,33.85)	20.69 (6.47,34.87)	21.47 (6.90,35.89)	22.11 (7.25,36.43)	22.64 (7.57,37.09)
30	6.71 (1.41,15.86)	11.03 (2.58,23.73)	13.98 (3.54,27.73)	16.10 (4.34,29.87)	17.68 (5.01,31.66)	18.91 (5.54,32.87)	19.89 (5.98,33.90)	20.68 (6.38,34.88)	21.34 (6.73,35.28)	21.89 (7.04,35.85)
20	6.14 (1.28,14.66)	10.23 (2.33,22.59)	13.08 (3.20,26.72)	15.16 (3.92,29.19)	16.73 (4.52,31.07)	17.97 (5.03,32.20)	18.96 (5.44,33.04)	19.77 (5.76,33.72)	20.44 (6.03,34.32)	21.01 (6.28,35.27)
10	5.56 (1.02,13.85)	9.36 (1.88,21.64)	12.08 (2.60,25.65)	14.11 (3.21,28.36)	15.66 (3.75,30.32)	16.89 (4.20,31.79)	17.88 (4.59,32.82)	18.70 (4.91,33.52)	19.39 (5.18,34.25)	19.97 (5.43,34.80)
0	4.96 (0.83,12.95)	8.44 (1.54,20.43)	11.00 (2.13,24.89)	12.93 (2.61,27.80)	14.44 (3.03,29.72)	15.64 (3.38,31.10)	16.63 (3.69,32.15)	17.44 (3.99,33.19)	18.13 (4.24,34.19)	18.72 (4.45,34.88)

(c) £18/dose; VaR; All-willing; 7.5 years

Uptake of 2nd dose (%) \ Initial vaccination uptake (%)	10	20	30	40	50	60	70	80	90	100
100	11.39 (2.70,24.83)	17.18 (4.88,30.97)	20.56 (6.52,34.31)	22.75 (7.85,36.45)	24.30 (8.96,38.04)	25.44 (9.86,39.16)	26.32 (10.58,39.90)	27.01 (11.12,40.48)	27.57 (11.59,40.94)	28.03 (12.00,41.40)
90	10.96 (2.66,23.86)	16.69 (4.81,30.53)	20.07 (6.46,33.56)	22.29 (7.72,35.98)	23.86 (8.71,37.44)	25.02 (9.49,38.66)	25.91 (10.20,39.61)	26.62 (10.78,40.21)	27.19 (11.23,40.63)	27.66 (11.59,41.06)
80	10.52 (2.50,23.12)	16.16 (4.51,30.14)	19.55 (6.13,33.35)	21.79 (7.43,35.46)	23.37 (8.48,37.06)	24.55 (9.28,38.02)	25.46 (9.93,39.02)	26.19 (10.46,39.90)	26.77 (10.92,40.40)	27.26 (11.32,40.81)
70	10.06 (2.42,22.35)	15.60 (4.32,29.27)	18.98 (5.81,32.82)	21.24 (7.01,34.85)	22.84 (8.02,36.75)	24.04 (8.84,37.97)	24.97 (9.52,38.75)	25.71 (10.09,39.31)	26.31 (10.55,39.80)	26.80 (10.91,40.47)
60	9.59 (2.36,21.27)	15.01 (4.26,28.75)	18.37 (5.74,32.55)	20.64 (6.92,34.26)	22.26 (7.86,35.85)	23.48 (8.62,37.14)	24.42 (9.25,38.26)	25.18 (9.76,38.81)	25.79 (10.20,39.39)	26.30 (10.60,40.07)
50	9.10 (2.18,20.02)	14.38 (3.96,28.11)	17.71 (5.33,31.89)	19.98 (6.47,33.91)	21.62 (7.45,35.17)	22.85 (8.27,36.51)	23.81 (8.90,37.55)	24.58 (9.40,38.63)	25.21 (9.83,39.00)	25.73 (10.22,39.50)
40	8.59 (2.03,19.27)	13.71 (3.66,27.22)	17.00 (4.98,30.91)	19.26 (6.05,33.00)	20.91 (6.94,34.77)	22.15 (7.67,36.05)	23.13 (8.30,37.16)	23.91 (8.84,37.85)	24.55 (9.30,38.66)	25.08 (9.70,39.18)
30	8.07 (1.92,18.20)	13.00 (3.45,26.14)	16.22 (4.69,29.84)	18.46 (5.70,32.30)	20.11 (6.53,33.67)	21.37 (7.22,35.04)	22.35 (7.79,36.05)	23.14 (8.26,36.83)	23.79 (8.67,37.45)	24.33 (9.02,38.04)
20	7.53 (1.74,17.24)	12.24 (3.13,25.46)	15.38 (4.25,29.18)	17.59 (5.15,31.63)	19.22 (5.90,33.21)	20.48 (6.52,34.19)	21.47 (6.96,35.29)	22.27 (7.35,36.25)	22.92 (7.72,36.95)	23.48 (8.06,37.41)
10	6.98 (1.47,16.71)	11.44 (2.67,24.45)	14.46 (3.64,28.52)	16.62 (4.43,31.12)	18.23 (5.10,32.90)	19.47 (5.62,33.88)	20.46 (6.01,35.07)	21.26 (6.37,35.77)	21.92 (6.68,36.33)	22.48 (6.97,36.88)
0	6.41 (1.26,15.87)	10.60 (2.27,23.59)	13.47 (3.11,27.97)	15.55 (3.79,30.47)	17.12 (4.36,32.19)	18.34 (4.80,33.80)	19.31 (5.19,34.88)	20.10 (5.53,35.72)	20.76 (5.84,36.13)	21.31 (6.09,36.83)

(d) £18/dose; VaR; Some-unwilling; 1.5, 3 years

Uptake of 2nd dose (%) \ Initial vaccination uptake (%)	10	20	30	40	50	60	70	80	90	100
100	2.59 (0.51,6.17)	5.18 (1.04,12.27)	7.75 (1.58,17.82)	10.25 (2.15,22.82)	12.63 (2.73,27.26)	14.85 (3.33,30.03)	16.86 (3.96,32.36)	18.64 (4.60,33.81)	20.19 (5.27,35.19)	21.50 (5.94,36.49)
90	2.53 (0.50,6.01)	5.06 (1.02,11.95)	7.57 (1.55,17.44)	10.01 (2.10,22.45)	12.34 (2.66,26.78)	14.52 (3.23,29.57)	16.49 (3.82,31.74)	18.25 (4.42,33.26)	19.78 (5.03,34.79)	21.09 (5.65,35.91)
80	2.46 (0.48,5.84)	4.92 (0.97,11.57)	7.36 (1.47,16.99)	9.74 (1.99,22.04)	12.01 (2.51,26.18)	14.13 (3.05,29.04)	16.07 (3.60,31.31)	17.80 (4.17,32.83)	19.32 (4.74,34.52)	20.62 (5.33,35.35)
70	2.38 (0.45,5.65)	4.76 (0.90,11.18)	7.12 (1.37,16.46)	9.42 (1.85,21.46)	11.62 (2.34,25.46)	13.69 (2.85,28.40)	15.58 (3.37,30.56)	17.28 (3.91,32.30)	18.78 (4.47,33.90)	20.08 (5.04,34.85)
60	2.29 (0.41,5.48)	4.57 (0.83,10.76)	6.83 (1.26,15.85)	9.04 (1.71,20.62)	11.17 (2.18,24.54)	13.17 (2.65,27.65)	15.02 (3.15,29.77)	16.69 (3.66,31.83)	18.17 (4.18,33.04)	19.46 (4.72,34.19)
50	2.17 (0.38,5.24)	4.35 (0.76,10.39)	6.50 (1.16,15.11)	8.61 (1.57,19.66)	10.64 (1.99,23.43)	12.57 (2.42,26.73)	14.36 (2.87,29.02)	15.99 (3.34,30.79)	17.44 (3.81,32.32)	18.73 (4.30,33.30)
40	2.04 (0.35,4.96)	4.09 (0.70,9.75)	6.11 (1.07,14.23)	8.10 (1.45,18.29)	10.03 (1.84,22.08)	11.86 (2.24,25.27)	13.58 (2.66,27.92)	15.16 (3.09,29.86)	16.59 (3.53,31.29)	17.87 (3.98,32.48)
30	1.89 (0.30,4.58)	3.78 (0.62,9.01)	5.65 (0.94,13.15)	7.50 (1.27,16.95)	9.30 (1.62,20.47)	11.03 (1.97,23.83)	12.66 (2.34,26.46)	14.19 (2.72,28.58)	15.58 (3.11,30.20)	16.84 (3.50,31.20)
20	1.70 (0.26,4.20)	3.41 (0.53,8.34)	5.11 (0.81,12.22)	6.79 (1.09,15.93)	8.44 (1.38,19.15)	10.04 (1.68,22.39)	11.57 (1.98,24.86)	13.01 (2.29,27.13)	14.35 (2.61,28.58)	15.58 (2.96,29.82)
10	1.49 (0.16,3.81)	2.98 (0.34,7.57)	4.47 (0.51,11.23)	5.95 (0.70,14.74)	7.42 (0.89,17.82)	8.85 (1.08,20.94)	10.24 (1.28,23.60)	11.57 (1.49,25.42)	12.82 (1.70,27.21)	14.00 (1.92,28.87)
0	1.23 (0.04,3.43)	2.47 (0.09,6.81)	3.71 (0.14,10.09)	4.95 (0.20,13.19)	6.19 (0.27,16.12)	7.41 (0.34,18.99)	8.60 (0.41,21.69)	9.77 (0.50,24.19)	10.90 (0.59,26.18)	11.97 (0.68,27.46)

(e) £18/dose; VaR; Some-unwilling; 4 years

Uptake of 2nd dose (%) \ Initial vaccination uptake (%)	10	20	30	40	50	60	70	80	90	100
100	3.16 (0.81,7.08)	6.34 (1.65,13.98)	9.49 (2.51,20.38)	12.56 (3.40,26.13)	15.45 (4.32,30.11)	18.11 (5.25,33.53)	20.46 (6.21,35.61)	22.48 (7.18,37.22)	24.18 (8.19,38.90)	25.58 (9.24,39.91)
90	3.09 (0.79,6.83)	6.19 (1.61,13.53)	9.27 (2.44,19.85)	12.27 (3.29,25.49)	15.11 (4.17,29.75)	17.73 (5.08,32.63)	20.06 (6.02,35.09)	22.08 (6.99,37.17)	23.79 (7.98,38.57)	25.21 (9.01,39.59)
80	3.01 (0.77,6.61)	6.02 (1.56,13.09)	9.02 (2.37,19.21)	11.95 (3.20,24.76)	14.73 (4.07,29.13)	17.31 (4.95,32.26)	19.62 (5.87,34.52)	21.64 (6.80,36.65)	23.37 (7.77,38.06)	24.81 (8.77,39.03)
70	2.92 (0.74,6.49)	5.84 (1.50,12.65)	8.75 (2.29,18.55)	11.60 (3.10,24.03)	14.32 (3.93,28.29)	16.85 (4.79,31.63)	19.14 (5.68,33.93)	21.16 (6.59,36.18)	22.89 (7.53,37.50)	24.36 (8.51,38.41)
60	2.82 (0.72,6.20)	5.64 (1.46,12.18)	8.46 (2.22,17.85)	11.22 (3.01,22.90)	13.86 (3.81,27.58)	16.34 (4.64,30.79)	18.60 (5.50,33.30)	20.62 (6.38,35.34)	22.37 (7.29,36.88)	23.86 (8.22,38.07)
50	2.71 (0.70,5.91)	5.43 (1.42,11.63)	8.13 (2.16,17.04)	10.80 (2.93,21.88)	13.36 (3.71,26.54)	15.78 (4.51,29.99)	18.01 (5.33,32.36)	20.01 (6.18,34.46)	21.77 (7.05,36.15)	23.29 (7.94,37.45)
40	2.59 (0.67,5.64)	5.18 (1.36,11.13)	7.78 (2.07,16.18)	10.33 (2.80,20.89)	12.81 (3.55,25.35)	15.16 (4.32,28.98)	17.35 (5.10,31.46)	19.33 (5.90,33.66)	21.10 (6.72,35.36)	22.64 (7.57,37.09)
30	2.45 (0.64,5.37)	4.92 (1.29,10.66)	7.38 (1.95,15.76)	9.82 (2.63,20.08)	12.20 (3.34,24.37)	14.47 (4.07,27.88)	16.60 (4.80,30.56)	18.56 (5.52,32.74)	20.33 (6.27,34.42)	21.89 (7.04,35.85)
20	2.31 (0.57,5.13)	4.63 (1.15,10.18)	6.95 (1.74,15.08)	9.26 (2.34,19.55)	11.52 (2.95,23.63)	13.69 (3.58,26.85)	15.76 (4.26,29.67)	17.68 (4.95,32.13)	19.43 (5.61,33.62)	21.01 (6.28,35.27)
10	2.15 (0.48,4.96)	4.31 (0.99,9.86)	6.48 (1.51,14.45)	8.63 (2.05,18.82)	10.76 (2.59,22.79)	12.83 (3.14,26.25)	14.80 (3.69,29.07)	16.67 (4.27,31.61)	18.39 (4.84,33.31)	19.97 (5.43,34.80)
0	1.97 (0.40,4.70)	3.96 (0.82,9.29)	5.95 (1.24,13.69)	7.95 (1.67,18.03)	9.92 (2.11,22.06)	11.85 (2.56,25.60)	13.72 (3.02,28.53)	15.50 (3.49,30.84)	17.17 (3.96,32.78)	18.72 (4.45,34.88)

(f) £18/dose; VaR; Some-unwilling; 7.5 years

Uptake of 2nd dose (%) \ Initial vaccination uptake (%)	10	20	30	40	50	60	70	80	90	100
100	3.60 (1.04,7.76)	7.22 (2.11,15.26)	10.81 (3.21,22.08)	14.27 (4.33,27.95)	17.50 (5.50,32.23)	20.39 (6.71,35.10)	22.89 (7.96,37.50)	24.98 (9.26,39.69)	26.68 (10.61,40.79)	28.03 (12.00,41.40)
90	3.50 (1.01,7.44)	7.02 (2.05,14.80)	10.51 (3.11,21.50)	13.89 (4.22,27.11)	17.06 (5.36,31.50)	19.92 (6.53,34.72)	22.42 (7.74,37.44)	24.53 (8.98,39.20)	26.27 (10.27,40.43)	27.66 (11.59,41.06)
80	3.39 (0.98,7.19)	6.80 (1.99,14.18)	10.19 (3.03,20.74)	13.49 (4.11,26.27)	16.59 (5.21,30.84)	19.42 (6.35,34.14)	21.91 (7.54,36.84)	24.04 (8.76,38.60)	25.81 (10.02,39.75)	27.26 (11.32,40.81)
70	3.28 (0.95,6.96)	6.58 (1.93,13.70)	9.86 (2.94,20.07)	13.06 (3.98,25.45)	16.09 (5.06,30.11)	18.88 (6.16,33.49)	21.37 (7.30,36.11)	23.51 (8.47,38.01)	25.32 (9.67,39.41)	26.80 (10.91,40.47)
60	3.16 (0.93,6.62)	6.34 (1.88,13.05)	9.50 (2.86,19.04)	12.60 (3.87,24.40)	15.56 (4.91,29.29)	18.30 (5.97,32.82)	20.77 (7.08,35.17)	22.92 (8.22,37.31)	24.76 (9.39,38.89)	26.30 (10.60,40.07)
50	3.03 (0.90,6.34)	6.08 (1.82,12.46)	9.13 (2.77,18.25)	12.12 (3.75,23.59)	14.99 (4.75,28.23)	17.67 (5.78,31.74)	20.12 (6.84,34.48)	22.28 (7.93,36.41)	24.15 (9.06,38.04)	25.73 (10.22,39.50)
40	2.89 (0.85,6.05)	5.81 (1.73,12.02)	8.73 (2.63,17.61)	11.60 (3.57,22.55)	14.38 (4.54,27.15)	16.99 (5.55,30.64)	19.40 (6.58,33.22)	21.56 (7.60,35.75)	23.45 (8.62,37.58)	25.08 (9.70,39.18)
30	2.75 (0.82,5.81)	5.53 (1.65,11.54)	8.30 (2.50,17.01)	11.05 (3.38,21.85)	13.72 (4.26,26.11)	16.26 (5.17,29.80)	18.62 (6.11,32.45)	20.76 (7.07,34.64)	22.67 (8.03,36.56)	24.33 (9.02,38.04)
20	2.60 (0.73,5.57)	5.22 (1.49,11.11)	7.85 (2.27,16.46)	10.46 (3.07,21.41)	13.01 (3.87,25.68)	15.46 (4.68,29.02)	17.76 (5.51,31.96)	19.87 (6.34,34.11)	21.78 (7.19,35.73)	23.48 (8.06,37.41)
10	2.44 (0.64,5.44)	4.90 (1.30,10.71)	7.38 (1.96,15.90)	9.84 (2.64,20.49)	12.25 (3.32,24.88)	14.59 (4.02,28.52)	16.81 (4.74,31.55)	18.88 (5.46,33.66)	20.77 (6.20,35.75)	22.48 (6.97,36.88)
0	2.27 (0.56,5.17)	4.56 (1.12,10.27)	6.87 (1.70,15.11)	9.17 (2.29,19.90)	11.44 (2.89,24.10)	13.65 (3.51,27.68)	15.77 (4.14,30.61)	17.77 (4.78,33.13)	19.62 (5.43,35.38)	21.31 (6.09,36.83)

(g) £18/dose; VoD; All-willing; 1.5, 3 years

Uptake of 2nd dose (%) \ Initial vaccination uptake (%)	10	20	30	40	50	60	70	80	90	100
100	2.34 (0.50,5.05)	4.11 (0.93,8.50)	5.50 (1.31,11.05)	6.62 (1.63,13.01)	7.53 (1.90,14.58)	8.30 (2.13,15.86)	8.95 (2.32,16.94)	9.50 (2.49,17.85)	9.98 (2.64,18.64)	10.39 (2.77,19.33)
90	2.20 (0.47,4.76)	3.90 (0.88,8.10)	5.24 (1.24,10.59)	6.33 (1.55,12.54)	7.24 (1.82,14.07)	8.00 (2.04,15.36)	8.64 (2.24,16.45)	9.20 (2.41,17.37)	9.68 (2.56,18.17)	10.11 (2.71,18.86)
80	2.06 (0.44,4.43)	3.67 (0.83,7.62)	4.97 (1.17,10.10)	6.03 (1.47,12.03)	6.92 (1.72,13.59)	7.67 (1.96,14.88)	8.32 (2.16,15.97)	8.88 (2.34,16.91)	9.37 (2.50,17.72)	9.80 (2.65,18.44)
70	1.92 (0.42,4.11)	3.44 (0.78,7.13)	4.68 (1.11,9.46)	5.71 (1.40,11.38)	6.58 (1.66,12.96)	7.33 (1.90,14.29)	7.97 (2.09,15.42)	8.53 (2.26,16.37)	9.02 (2.40,17.22)	9.46 (2.52,17.95)
60	1.77 (0.38,3.78)	3.20 (0.73,6.67)	4.39 (1.03,8.97)	5.38 (1.32,10.78)	6.22 (1.56,12.31)	6.95 (1.78,13.61)	7.59 (1.96,14.73)	8.15 (2.13,15.70)	8.64 (2.27,16.56)	9.09 (2.40,17.29)
50	1.62 (0.33,3.49)	2.96 (0.63,6.23)	4.07 (0.91,8.42)	5.02 (1.17,10.24)	5.84 (1.40,11.74)	6.56 (1.62,13.05)	7.18 (1.81,14.13)	7.74 (1.97,15.06)	8.23 (2.12,15.88)	8.68 (2.25,16.60)
40	1.47 (0.29,3.22)	2.70 (0.56,5.75)	3.75 (0.81,7.79)	4.65 (1.04,9.50)	5.44 (1.24,10.95)	6.13 (1.43,12.19)	6.74 (1.61,13.31)	7.29 (1.77,14.28)	7.78 (1.92,15.13)	8.22 (2.05,15.90)
30	1.32 (0.24,2.88)	2.44 (0.46,5.24)	3.41 (0.67,7.18)	4.25 (0.86,8.79)	5.00 (1.03,10.17)	5.66 (1.19,11.40)	6.26 (1.34,12.47)	6.79 (1.47,13.43)	7.28 (1.59,14.28)	7.72 (1.72,15.06)
20	1.16 (0.18,2.56)	2.17 (0.35,4.68)	3.05 (0.52,6.47)	3.83 (0.67,8.01)	4.53 (0.81,9.35)	5.16 (0.92,10.55)	5.73 (1.04,11.62)	6.25 (1.16,12.59)	6.72 (1.27,13.45)	7.15 (1.37,14.25)
10	1.00 (0.11,2.34)	1.89 (0.22,4.34)	2.68 (0.32,6.04)	3.39 (0.42,7.51)	4.03 (0.51,8.80)	4.61 (0.60,10.02)	5.15 (0.68,11.10)	5.64 (0.75,12.08)	6.10 (0.83,12.93)	6.52 (0.89,13.70)
0	0.84 (0.02,2.10)	1.59 (0.04,3.92)	2.28 (0.06,5.54)	2.91 (0.08,6.99)	3.48 (0.10,8.30)	4.02 (0.12,9.46)	4.51 (0.14,10.49)	4.97 (0.16,11.44)	5.40 (0.18,12.34)	5.80 (0.20,13.18)

(h) £18/dose; VoD; All-willing; 4 years

Uptake of 2nd dose (%) \ Initial vaccination uptake (%)	10	20	30	40	50	60	70	80	90	100
100	3.53 (1.10,7.04)	6.18 (2.04,11.75)	8.25 (2.84,15.20)	9.91 (3.52,17.83)	11.27 (4.11,19.88)	12.40 (4.61,21.52)	13.37 (5.04,22.92)	14.20 (5.42,24.17)	14.92 (5.74,25.25)	15.55 (6.01,26.19)
90	3.38 (1.08,6.69)	5.94 (1.98,11.35)	7.96 (2.76,14.74)	9.59 (3.43,17.33)	10.94 (4.01,19.38)	12.07 (4.52,21.05)	13.04 (4.96,22.42)	13.87 (5.33,23.60)	14.59 (5.65,24.62)	15.23 (5.93,25.58)
80	3.22 (1.03,6.35)	5.70 (1.93,10.77)	7.66 (2.70,14.18)	9.27 (3.37,16.79)	10.60 (3.97,18.83)	11.72 (4.46,20.51)	12.69 (4.88,21.93)	13.52 (5.26,23.16)	14.25 (5.59,24.24)	14.89 (5.87,25.16)
70	3.06 (0.98,6.02)	5.45 (1.82,10.38)	7.36 (2.56,13.69)	8.93 (3.19,16.24)	10.24 (3.75,18.27)	11.35 (4.24,19.99)	12.31 (4.69,21.49)	13.15 (5.09,22.69)	13.88 (5.44,23.74)	14.53 (5.75,24.67)
60	2.90 (0.92,5.71)	5.19 (1.72,9.86)	7.04 (2.42,13.09)	8.57 (3.04,15.66)	9.86 (3.57,17.68)	10.96 (4.05,19.35)	11.92 (4.46,20.76)	12.75 (4.84,21.96)	13.49 (5.17,23.00)	14.14 (5.46,23.91)
50	2.74 (0.88,5.42)	4.92 (1.65,9.41)	6.71 (2.33,12.48)	8.20 (2.92,14.92)	9.46 (3.43,16.90)	10.55 (3.90,18.55)	11.50 (4.31,19.95)	12.33 (4.67,21.16)	13.06 (5.00,22.21)	13.72 (5.30,23.21)
40	2.57 (0.84,5.10)	4.65 (1.58,8.88)	6.37 (2.23,11.82)	7.81 (2.80,14.24)	9.05 (3.31,16.32)	10.11 (3.76,18.04)	11.05 (4.16,19.46)	11.87 (4.52,20.67)	12.61 (4.85,21.71)	13.26 (5.14,22.63)
30	2.40 (0.74,4.71)	4.37 (1.39,8.35)	6.01 (1.97,11.32)	7.41 (2.48,13.70)	8.61 (2.94,15.70)	9.65 (3.36,17.31)	10.57 (3.73,18.82)	11.38 (4.07,20.13)	12.11 (4.37,21.22)	12.77 (4.65,22.23)
20	2.23 (0.66,4.50)	4.08 (1.25,8.00)	5.64 (1.76,10.87)	6.98 (2.22,13.26)	8.14 (2.64,15.26)	9.16 (3.01,16.96)	10.06 (3.35,18.37)	10.86 (3.64,19.69)	11.58 (3.90,20.88)	12.23 (4.14,21.92)
10	2.06 (0.54,4.25)	3.79 (1.03,7.67)	5.26 (1.46,10.42)	6.53 (1.85,12.74)	7.65 (2.20,14.67)	8.63 (2.52,16.33)	9.51 (2.81,17.76)	10.29 (3.07,19.01)	11.00 (3.32,20.12)	11.64 (3.54,21.12)
0	1.89 (0.45,4.10)	3.49 (0.86,7.34)	4.86 (1.23,10.04)	6.07 (1.56,12.33)	7.13 (1.86,14.19)	8.07 (2.13,15.81)	8.91 (2.37,17.29)	9.67 (2.58,18.59)	10.37 (2.78,19.76)	11.00 (2.96,20.77)

(i) £18/dose; VoD; All-willing; 7.5 years

Uptake of 2nd dose (%)	10	20	30	40	50	60	70	80	90	100
100	4.30 (1.49,8.33)	7.54 (2.77,13.79)	10.05 (3.86,17.75)	12.07 (4.81,20.65)	13.73 (5.62,23.06)	15.11 (6.32,25.07)	16.29 (6.91,26.75)	17.30 (7.42,28.12)	18.18 (7.87,29.37)	18.95 (8.27,30.35)
90	4.14 (1.47,7.95)	7.27 (2.73,13.39)	9.73 (3.79,17.24)	11.71 (4.69,20.26)	13.35 (5.46,22.57)	14.72 (6.15,24.42)	15.89 (6.76,26.06)	16.90 (7.30,27.41)	17.78 (7.77,28.67)	18.55 (8.19,29.72)
80	3.96 (1.43,7.58)	7.00 (2.65,12.84)	9.40 (3.69,16.73)	11.34 (4.58,19.70)	12.96 (5.35,21.98)	14.31 (6.02,23.98)	15.48 (6.63,25.61)	16.48 (7.19,27.01)	17.36 (7.65,28.12)	18.14 (8.06,29.21)
70	3.79 (1.35,7.20)	6.72 (2.51,12.39)	9.05 (3.51,16.15)	10.96 (4.37,19.01)	12.55 (5.12,21.36)	13.89 (5.78,23.34)	15.04 (6.36,24.93)	16.04 (6.87,26.37)	16.92 (7.32,27.67)	17.69 (7.73,28.80)
60	3.61 (1.29,6.86)	6.43 (2.40,11.85)	8.70 (3.37,15.49)	10.56 (4.20,18.35)	12.12 (4.92,20.63)	13.44 (5.54,22.47)	14.58 (6.08,24.04)	15.57 (6.55,25.49)	16.45 (6.97,26.87)	17.22 (7.35,27.84)
50	3.44 (1.23,6.57)	6.14 (2.30,11.27)	8.33 (3.24,14.91)	10.15 (4.05,17.66)	11.67 (4.76,19.86)	12.97 (5.36,21.65)	14.10 (5.91,23.32)	15.08 (6.40,24.67)	15.95 (6.85,25.92)	16.72 (7.23,27.03)
40	3.26 (1.17,6.19)	5.85 (2.19,10.85)	7.96 (3.08,14.28)	9.72 (3.86,17.07)	11.20 (4.55,19.28)	12.48 (5.16,21.16)	13.59 (5.69,22.82)	14.56 (6.16,24.25)	15.41 (6.56,25.56)	16.18 (6.93,26.69)
30	3.07 (1.04,5.86)	5.54 (1.95,10.32)	7.57 (2.76,13.79)	9.27 (3.47,16.50)	10.71 (4.10,18.71)	11.96 (4.65,20.63)	13.04 (5.15,22.33)	14.00 (5.59,23.80)	14.85 (5.98,24.97)	15.60 (6.34,26.14)
20	2.89 (0.97,5.67)	5.23 (1.81,9.90)	7.17 (2.55,13.26)	8.80 (3.18,16.08)	10.20 (3.71,18.39)	11.41 (4.20,20.33)	12.47 (4.64,21.99)	13.41 (5.04,23.34)	14.24 (5.37,24.52)	14.99 (5.65,25.56)
10	2.70 (0.82,5.43)	4.91 (1.54,9.52)	6.75 (2.17,12.84)	8.32 (2.72,15.49)	9.66 (3.21,17.77)	10.83 (3.64,19.59)	11.86 (4.02,21.30)	12.77 (4.37,22.71)	13.59 (4.68,23.85)	14.32 (4.96,25.01)
0	2.51 (0.68,5.22)	4.58 (1.29,9.24)	6.33 (1.83,12.48)	7.81 (2.31,15.05)	9.10 (2.75,17.17)	10.22 (3.15,19.10)	11.22 (3.51,20.66)	12.10 (3.83,21.96)	12.89 (4.13,23.19)	13.60 (4.38,24.35)

Initial vaccination uptake (%)

(j) £18/dose; VoD; Some-unwilling; 1.5, 3 years

Uptake of 2nd dose (%)	10	20	30	40	50	60	70	80	90	100
100	1.28 (0.28,2.80)	2.51 (0.56,5.41)	3.69 (0.85,7.81)	4.81 (1.13,10.03)	5.87 (1.42,12.05)	6.88 (1.69,13.98)	7.84 (1.97,15.82)	8.74 (2.24,17.31)	9.60 (2.53,18.78)	10.40 (2.82,19.97)
90	1.24 (0.26,2.72)	2.43 (0.53,5.25)	3.57 (0.80,7.60)	4.66 (1.08,9.76)	5.69 (1.34,11.74)	6.67 (1.59,13.59)	7.61 (1.85,15.46)	8.49 (2.11,16.94)	9.32 (2.37,18.33)	10.11 (2.65,19.59)
80	1.19 (0.25,2.63)	2.34 (0.50,5.09)	3.44 (0.74,7.36)	4.49 (1.00,9.46)	5.49 (1.26,11.40)	6.44 (1.50,13.18)	7.35 (1.73,14.98)	8.21 (1.97,16.55)	9.02 (2.21,17.86)	9.79 (2.46,19.15)
70	1.14 (0.23,2.53)	2.24 (0.46,4.90)	3.30 (0.69,7.10)	4.31 (0.91,9.14)	5.27 (1.15,11.05)	6.19 (1.40,12.77)	7.07 (1.62,14.44)	7.90 (1.83,16.11)	8.69 (2.04,17.43)	9.45 (2.26,18.61)
60	1.09 (0.22,2.43)	2.14 (0.43,4.70)	3.14 (0.64,6.82)	4.11 (0.84,8.79)	5.04 (1.05,10.67)	5.92 (1.26,12.31)	6.76 (1.48,13.88)	7.57 (1.70,15.46)	8.33 (1.89,16.91)	9.06 (2.08,18.13)
50	1.03 (0.19,2.31)	2.02 (0.39,4.48)	2.98 (0.58,6.51)	3.89 (0.77,8.41)	4.78 (0.95,10.19)	5.62 (1.14,11.84)	6.43 (1.32,13.34)	7.20 (1.50,14.76)	7.94 (1.71,16.21)	8.65 (1.90,17.56)
40	0.96 (0.17,2.18)	1.89 (0.34,4.24)	2.79 (0.51,6.17)	3.66 (0.69,7.99)	4.49 (0.85,9.68)	5.29 (1.02,11.32)	6.06 (1.18,12.74)	6.80 (1.34,14.12)	7.51 (1.50,15.42)	8.19 (1.65,16.73)
30	0.89 (0.14,2.04)	1.75 (0.28,3.97)	2.59 (0.42,5.80)	3.40 (0.57,7.52)	4.18 (0.72,9.14)	4.93 (0.87,10.66)	5.66 (1.01,12.15)	6.36 (1.15,13.41)	7.03 (1.29,14.67)	7.68 (1.42,15.84)
20	0.81 (0.11,1.90)	1.60 (0.22,3.69)	2.37 (0.33,5.39)	3.12 (0.45,7.00)	3.84 (0.57,8.53)	4.54 (0.69,9.98)	5.22 (0.81,11.34)	5.87 (0.93,12.68)	6.50 (1.05,13.88)	7.11 (1.18,14.98)
10	0.73 (0.08,1.74)	1.44 (0.17,3.41)	2.13 (0.26,5.01)	2.81 (0.35,6.52)	3.46 (0.44,7.90)	4.10 (0.53,9.22)	4.73 (0.62,10.52)	5.33 (0.72,11.74)	5.92 (0.81,12.91)	6.49 (0.90,14.07)
0	0.63 (0.04,1.56)	1.26 (0.09,3.06)	1.87 (0.13,4.48)	2.46 (0.18,5.84)	3.05 (0.23,7.15)	3.62 (0.29,8.41)	4.18 (0.34,9.64)	4.73 (0.40,10.81)	5.26 (0.46,11.93)	5.78 (0.53,12.90)

Initial vaccination uptake (%)

(k) £18/dose; VoD; Some-unwilling; 4 years

Uptake of 2nd dose (%)	10	20	30	40	50	60	70	80	90	100
100	1.92 (0.58,3.90)	3.76 (1.16,7.59)	5.54 (1.75,10.99)	7.23 (2.35,14.08)	8.84 (2.96,16.81)	10.36 (3.57,19.39)	11.79 (4.14,21.65)	13.13 (4.70,23.67)	14.39 (5.26,25.31)	15.57 (5.83,26.90)
90	1.86 (0.56,3.82)	3.66 (1.12,7.41)	5.39 (1.69,10.72)	7.04 (2.27,13.75)	8.61 (2.86,16.43)	10.10 (3.45,18.99)	11.51 (4.02,21.21)	12.83 (4.55,23.27)	14.07 (5.06,24.93)	15.24 (5.62,26.43)
80	1.81 (0.54,3.72)	3.55 (1.08,7.21)	5.23 (1.63,10.44)	6.84 (2.19,13.41)	8.37 (2.76,16.03)	9.83 (3.33,18.56)	11.21 (3.88,20.77)	12.51 (4.39,22.89)	13.73 (4.89,24.52)	14.88 (5.41,25.98)
70	1.75 (0.52,3.61)	3.44 (1.04,7.00)	5.07 (1.57,10.14)	6.63 (2.11,13.05)	8.12 (2.65,15.70)	9.54 (3.20,18.11)	10.89 (3.72,20.29)	12.16 (4.22,22.26)	13.37 (4.73,24.09)	14.50 (5.23,25.55)
60	1.68 (0.49,3.49)	3.32 (0.99,6.77)	4.89 (1.50,9.83)	6.41 (2.02,12.66)	7.86 (2.54,15.26)	9.24 (3.06,17.63)	10.55 (3.57,19.78)	11.80 (4.04,21.74)	12.98 (4.54,23.64)	14.10 (5.03,25.08)
50	1.62 (0.47,3.36)	3.19 (0.95,6.53)	4.71 (1.43,9.49)	6.17 (1.92,12.25)	7.57 (2.42,14.79)	8.91 (2.92,17.10)	10.19 (3.40,19.24)	11.41 (3.87,21.18)	12.57 (4.32,22.94)	13.66 (4.76,24.55)
40	1.55 (0.45,3.23)	3.05 (0.90,6.27)	4.51 (1.36,9.14)	5.92 (1.82,11.81)	7.27 (2.29,14.29)	8.57 (2.75,16.56)	9.81 (3.21,18.66)	11.00 (3.67,20.57)	12.13 (4.09,22.33)	13.20 (4.51,23.91)
30	1.47 (0.42,3.09)	2.91 (0.85,6.01)	4.30 (1.28,8.76)	5.65 (1.72,11.34)	6.95 (2.14,13.75)	8.20 (2.56,15.98)	9.41 (2.99,18.03)	10.56 (3.43,19.92)	11.66 (3.86,21.66)	12.71 (4.27,23.25)
20	1.39 (0.39,2.93)	2.75 (0.79,5.72)	4.08 (1.18,8.36)	5.37 (1.58,10.84)	6.61 (1.98,13.17)	7.82 (2.37,15.34)	8.97 (2.77,17.36)	10.09 (3.17,19.22)	11.15 (3.57,20.80)	12.17 (3.98,22.53)
10	1.31 (0.36,2.80)	2.59 (0.72,5.44)	3.85 (1.08,7.98)	5.07 (1.45,10.31)	6.25 (1.82,12.55)	7.40 (2.20,14.66)	8.51 (2.58,16.63)	9.58 (2.94,18.48)	10.61 (3.30,20.16)	11.60 (3.66,21.62)
0	1.22 (0.32,2.65)	2.42 (0.65,5.15)	3.60 (0.97,7.53)	4.75 (1.31,9.83)	5.87 (1.64,11.94)	6.96 (1.98,13.92)	8.01 (2.33,15.81)	9.04 (2.68,17.63)	10.03 (3.03,19.35)	10.98 (3.38,20.89)

Initial vaccination uptake (%)

(l) £18/dose; VoD; Some-unwilling; 7.5 years

Uptake of 2nd dose (%)	10	20	30	40	50	60	70	80	90	100
100	2.36 (0.80,4.70)	4.65 (1.61,9.08)	6.85 (2.41,13.18)	8.94 (3.23,16.84)	10.91 (4.05,20.09)	12.77 (4.89,22.94)	14.51 (5.68,25.45)	16.12 (6.49,27.51)	17.61 (7.30,29.50)	18.99 (8.09,31.26)
90	2.29 (0.77,4.56)	4.51 (1.55,8.86)	6.65 (2.33,12.83)	8.68 (3.12,16.42)	10.61 (3.92,19.57)	12.43 (4.72,22.46)	14.13 (5.50,24.90)	15.72 (6.27,26.93)	17.20 (7.07,29.02)	18.57 (7.82,30.90)
80	2.22 (0.74,4.43)	4.37 (1.50,8.62)	6.44 (2.25,12.47)	8.42 (3.02,15.98)	10.30 (3.79,19.10)	12.08 (4.55,21.95)	13.75 (5.32,24.31)	15.31 (6.05,26.41)	16.77 (6.81,28.45)	18.12 (7.50,30.21)
70	2.14 (0.71,4.30)	4.22 (1.44,8.34)	6.22 (2.16,12.10)	8.14 (2.89,15.53)	9.98 (3.63,18.59)	11.71 (4.36,21.40)	13.35 (5.11,23.81)	14.88 (5.84,25.95)	16.32 (6.49,27.95)	17.66 (7.17,29.66)
60	2.06 (0.69,4.15)	4.06 (1.37,8.06)	6.00 (2.06,11.70)	7.86 (2.75,15.05)	9.64 (3.46,18.10)	11.33 (4.18,20.78)	12.93 (4.87,23.28)	14.43 (5.55,25.35)	15.84 (6.20,27.26)	17.17 (6.87,29.10)
50	1.98 (0.66,3.99)	3.90 (1.31,7.77)	5.77 (1.96,11.30)	7.56 (2.62,14.56)	9.29 (3.30,17.54)	10.93 (3.99,20.10)	12.49 (4.61,22.66)	13.96 (5.25,24.70)	15.35 (5.86,26.59)	16.65 (6.52,28.53)
40	1.89 (0.62,3.82)	3.74 (1.25,7.46)	5.53 (1.87,10.86)	7.26 (2.49,14.04)	8.92 (3.14,16.95)	10.51 (3.75,19.61)	12.03 (4.35,21.96)	13.46 (4.97,24.13)	14.82 (5.55,26.00)	16.11 (6.16,27.86)
30	1.80 (0.58,3.66)	3.56 (1.17,7.14)	5.28 (1.77,10.43)	6.94 (2.36,13.47)	8.54 (2.94,16.33)	10.08 (3.52,18.94)	11.54 (4.09,21.16)	12.94 (4.68,23.45)	14.27 (5.27,25.36)	15.53 (5.83,27.03)
20	1.71 (0.54,3.50)	3.38 (1.10,6.81)	5.02 (1.65,9.97)	6.61 (2.22,12.92)	8.14 (2.78,15.63)	9.62 (3.33,18.21)	11.04 (3.87,20.56)	12.40 (4.41,22.58)	13.69 (4.94,24.62)	14.92 (5.48,26.29)
10	1.61 (0.50,3.35)	3.20 (1.01,6.48)	4.75 (1.53,9.53)	6.26 (2.05,12.34)	7.73 (2.58,14.97)	9.14 (3.11,17.38)	10.51 (3.64,19.75)	11.82 (4.15,21.87)	13.08 (4.66,23.80)	14.28 (5.16,25.54)
0	1.52 (0.46,3.20)	3.01 (0.93,6.20)	4.47 (1.40,9.03)	5.90 (1.88,11.81)	7.29 (2.36,14.31)	8.64 (2.85,16.66)	9.95 (3.35,18.90)	11.21 (3.85,21.06)	12.43 (4.35,22.84)	13.59 (4.82,24.66)

Initial vaccination uptake (%)

(m) £85/dose; VaR; All-willing; 1.5, 3 years

Uptake of 2nd dose (%) \ Initial vaccination uptake (%)	10	20	30	40	50	60	70	80	90	100
100	-1.74 (-6.40,7.26)	-2.58 (-9.99,8.20)	-2.96 (-12.27,8.95)	-3.12 (-13.71,9.57)	-3.17 (-14.71,9.66)	-3.18 (-15.50,9.86)	-3.17 (-16.18,10.28)	-3.17 (-16.61,10.50)	-3.18 (-17.14,10.67)	-3.20 (-17.53,10.81)
90	-1.74 (-6.07,7.00)	-2.63 (-9.67,8.06)	-3.10 (-11.92,8.64)	-3.33 (-13.50,9.30)	-3.44 (-14.57,9.33)	-3.50 (-15.43,9.65)	-3.53 (-16.12,9.69)	-3.55 (-16.65,10.06)	-3.58 (-17.17,10.16)	-3.61 (-17.57,10.36)
80	-1.74 (-5.76,6.65)	-2.68 (-9.32,7.49)	-3.22 (-11.63,8.26)	-3.54 (-13.24,8.68)	-3.72 (-14.41,9.00)	-3.83 (-15.34,9.28)	-3.91 (-16.08,9.49)	-3.96 (-16.67,9.38)	-4.01 (-17.22,9.65)	-4.06 (-17.56,9.69)
70	-1.74 (-5.45,6.14)	-2.73 (-8.92,7.33)	-3.35 (-11.30,8.07)	-3.74 (-12.91,8.23)	-4.00 (-14.20,8.60)	-4.18 (-15.32,8.86)	-4.30 (-16.04,9.07)	-4.40 (-16.67,9.22)	-4.48 (-17.22,9.12)	-4.56 (-17.68,9.09)
60	-1.73 (-5.11,5.55)	-2.77 (-8.51,6.84)	-3.47 (-10.94,7.51)	-3.95 (-12.60,7.86)	-4.28 (-13.91,8.09)	-4.53 (-15.00,8.24)	-4.72 (-15.90,8.24)	-4.87 (-16.60,8.47)	-5.00 (-17.24,8.63)	-5.10 (-17.79,8.57)
50	-1.73 (-4.79,4.78)	-2.82 (-8.12,6.39)	-3.58 (-10.54,7.16)	-4.14 (-12.30,7.10)	-4.57 (-13.64,7.35)	-4.89 (-14.78,7.82)	-5.15 (-15.67,7.64)	-5.36 (-16.44,7.39)	-5.54 (-17.17,7.33)	-5.70 (-17.78,7.44)
40	-1.72 (-4.46,3.98)	-2.86 (-7.63,5.93)	-3.70 (-10.01,6.63)	-4.34 (-11.83,6.63)	-4.85 (-13.23,6.67)	-5.26 (-14.45,6.72)	-5.60 (-15.52,6.56)	-5.88 (-16.39,6.44)	-6.13 (-17.19,6.58)	-6.34 (-17.87,6.41)
30	-1.72 (-4.08,3.24)	-2.91 (-7.11,5.01)	-3.82 (-9.44,5.84)	-4.54 (-11.32,6.42)	-5.14 (-12.79,5.69)	-5.64 (-14.06,5.77)	-6.06 (-15.24,5.69)	-6.42 (-16.27,5.46)	-6.74 (-17.05,5.36)	-7.03 (-17.79,5.31)
20	-1.71 (-3.72,2.82)	-2.96 (-6.57,4.06)	-3.94 (-8.86,4.98)	-4.75 (-10.68,5.24)	-5.44 (-12.33,5.40)	-6.03 (-13.76,5.39)	-6.54 (-14.99,4.92)	-7.00 (-16.06,5.06)	-7.40 (-17.00,4.90)	-7.76 (-17.76,4.73)
10	-1.69 (-3.43,2.31)	-3.00 (-6.19,3.69)	-4.07 (-8.46,4.37)	-4.98 (-10.37,4.64)	-5.76 (-12.04,4.65)	-6.45 (-13.43,4.77)	-7.06 (-14.82,4.77)	-7.61 (-16.03,4.66)	-8.11 (-17.13,4.32)	-8.57 (-18.14,4.28)
0	-1.68 (-3.27,1.70)	-3.05 (-5.96,2.91)	-4.21 (-8.24,3.99)	-5.22 (-10.24,4.40)	-6.11 (-11.96,4.45)	-6.91 (-13.52,4.62)	-7.64 (-14.98,4.50)	-8.30 (-16.27,4.43)	-8.91 (-17.49,4.32)	-9.47 (-18.60,4.09)

(n) £85/dose; VaR; All-willing; 4 years

Uptake of 2nd dose (%) \ Initial vaccination uptake (%)	10	20	30	40	50	60	70	80	90	100
100	1.63 (-4.40,11.90)	2.50 (-6.71,14.15)	3.06 (-7.99,15.77)	3.50 (-8.79,16.44)	3.85 (-9.34,17.05)	4.12 (-9.71,17.61)	4.34 (-9.95,17.85)	4.51 (-10.15,18.12)	4.65 (-10.37,18.25)	4.74 (-10.60,18.38)
90	1.61 (-4.13,11.85)	2.50 (-6.47,14.16)	3.06 (-7.65,15.96)	3.48 (-8.46,16.20)	3.81 (-9.04,17.14)	4.07 (-9.52,17.56)	4.28 (-9.80,17.87)	4.44 (-9.98,18.11)	4.56 (-10.23,18.29)	4.65 (-10.49,18.40)
80	1.59 (-3.81,11.53)	2.50 (-6.00,14.23)	3.06 (-7.27,15.79)	3.47 (-8.10,16.62)	3.79 (-8.70,16.99)	4.03 (-9.21,17.47)	4.22 (-9.47,17.72)	4.37 (-9.78,18.21)	4.49 (-9.98,18.35)	4.58 (-10.24,18.41)
70	1.56 (-3.51,11.12)	2.49 (-5.62,14.15)	3.07 (-6.98,15.60)	3.47 (-7.69,16.61)	3.77 (-8.39,16.97)	4.01 (-8.84,17.46)	4.19 (-9.22,17.66)	4.33 (-9.52,17.88)	4.43 (-9.73,18.27)	4.52 (-10.03,18.57)
60	1.53 (-3.25,10.50)	2.48 (-5.28,14.12)	3.07 (-6.53,15.23)	3.48 (-7.47,16.38)	3.77 (-8.05,16.87)	4.00 (-8.47,17.21)	4.17 (-8.75,17.44)	4.30 (-9.14,17.76)	4.40 (-9.48,17.94)	4.47 (-9.81,18.07)
50	1.50 (-3.01,9.92)	2.46 (-4.88,13.71)	3.07 (-6.11,15.05)	3.48 (-7.03,15.98)	3.78 (-7.60,16.77)	4.00 (-8.12,17.14)	4.16 (-8.50,17.41)	4.29 (-8.83,17.55)	4.38 (-9.16,17.66)	4.45 (-9.35,17.99)
40	1.46 (-2.74,9.19)	2.43 (-4.53,13.39)	3.06 (-5.71,14.79)	3.48 (-6.64,15.58)	3.79 (-7.31,16.34)	4.01 (-7.83,16.70)	4.17 (-8.27,17.14)	4.29 (-8.60,17.54)	4.38 (-8.89,17.90)	4.44 (-9.18,18.14)
30	1.41 (-2.51,8.91)	2.39 (-4.14,12.89)	3.04 (-5.32,14.67)	3.48 (-6.28,15.75)	3.79 (-6.90,16.27)	4.02 (-7.44,16.75)	4.18 (-7.88,17.05)	4.30 (-8.26,17.30)	4.39 (-8.58,17.67)	4.46 (-8.88,17.76)
20	1.37 (-2.27,8.55)	2.34 (-3.85,12.85)	3.00 (-5.00,14.81)	3.46 (-5.87,16.00)	3.79 (-6.57,16.70)	4.02 (-7.19,17.07)	4.19 (-7.72,17.37)	4.32 (-8.13,17.77)	4.41 (-8.45,17.93)	4.47 (-8.74,17.91)
10	1.32 (-2.16,8.39)	2.28 (-3.69,12.87)	2.95 (-4.85,14.99)	3.42 (-5.76,16.54)	3.76 (-6.48,17.12)	4.01 (-7.19,17.72)	4.19 (-7.67,18.04)	4.32 (-8.19,18.27)	4.42 (-8.58,18.29)	4.49 (-8.93,18.51)
0	1.27 (-2.02,8.03)	2.21 (-3.49,12.73)	2.88 (-4.56,15.40)	3.36 (-5.53,16.66)	3.71 (-6.36,17.70)	3.97 (-7.08,18.32)	4.16 (-7.71,18.77)	4.30 (-8.26,19.34)	4.41 (-8.81,19.46)	4.48 (-9.32,19.58)

(o) £85/dose; VaR; All-willing; 7.5 years

Uptake of 2nd dose (%) \ Initial vaccination uptake (%)	10	20	30	40	50	60	70	80	90	100
100	3.88 (-3.03,14.93)	5.87 (-4.48,18.15)	7.06 (-4.98,19.68)	7.90 (-5.26,20.99)	8.53 (-5.35,21.80)	9.01 (-5.30,22.42)	9.39 (-5.37,22.71)	9.69 (-5.45,22.78)	9.93 (-5.53,22.89)	10.12 (-5.51,23.17)
90	3.82 (-2.82,14.75)	5.84 (-4.15,18.13)	7.07 (-4.70,20.10)	7.92 (-5.03,20.97)	8.55 (-5.03,21.66)	9.03 (-5.08,22.55)	9.41 (-5.09,22.84)	9.71 (-5.18,23.11)	9.95 (-5.33,23.18)	10.14 (-5.40,23.31)
80	3.74 (-2.60,14.50)	5.81 (-3.89,18.11)	7.07 (-4.44,20.00)	7.93 (-4.63,20.97)	8.57 (-4.70,21.89)	9.06 (-4.82,22.36)	9.43 (-4.91,22.84)	9.73 (-4.99,23.13)	9.97 (-4.97,23.35)	10.16 (-4.95,23.60)
70	3.67 (-2.39,14.10)	5.76 (-3.61,17.99)	7.05 (-4.14,19.80)	7.94 (-4.41,20.90)	8.59 (-4.56,21.60)	9.08 (-4.62,22.26)	9.46 (-4.62,22.80)	9.76 (-4.67,23.27)	10.00 (-4.85,23.46)	10.19 (-4.98,23.60)
60	3.58 (-2.21,13.62)	5.70 (-3.29,17.85)	7.02 (-3.88,19.59)	7.94 (-4.23,20.85)	8.60 (-4.44,21.61)	9.10 (-4.58,22.12)	9.48 (-4.64,22.81)	9.79 (-4.65,23.20)	10.03 (-4.66,23.42)	10.22 (-4.67,23.95)
50	3.49 (-2.00,13.00)	5.62 (-2.99,17.48)	6.98 (-3.58,19.48)	7.91 (-3.96,20.62)	8.59 (-4.15,21.68)	9.10 (-4.27,22.28)	9.50 (-4.33,22.75)	9.81 (-4.38,23.22)	10.05 (-4.41,23.63)	10.25 (-4.44,23.83)
40	3.39 (-1.77,12.53)	5.52 (-2.73,17.19)	6.91 (-3.28,19.02)	7.87 (-3.59,20.62)	8.57 (-3.80,21.30)	9.09 (-3.95,22.09)	9.50 (-4.06,22.97)	9.81 (-4.15,23.05)	10.06 (-4.23,23.50)	10.27 (-4.30,23.93)
30	3.29 (-1.64,12.28)	5.41 (-2.57,16.99)	6.82 (-3.14,19.12)	7.80 (-3.49,20.36)	8.52 (-3.70,21.23)	9.06 (-3.85,22.01)	9.47 (-3.97,22.65)	9.80 (-4.06,23.13)	10.06 (-4.14,23.44)	10.26 (-4.21,23.69)
20	3.17 (-1.54,11.97)	5.28 (-2.39,16.73)	6.69 (-2.95,19.23)	7.69 (-3.33,20.91)	8.43 (-3.64,21.53)	8.98 (-3.83,22.39)	9.41 (-3.93,22.85)	9.75 (-4.03,23.22)	10.01 (-4.12,23.56)	10.23 (-4.28,23.79)
10	3.05 (-1.51,11.61)	5.12 (-2.46,16.76)	6.54 (-3.04,19.68)	7.55 (-3.45,21.14)	8.30 (-3.74,22.12)	8.86 (-3.97,22.88)	9.30 (-4.16,23.36)	9.65 (-4.32,23.76)	9.92 (-4.46,24.00)	10.15 (-4.57,24.46)
0	2.93 (-1.39,11.37)	4.95 (-2.29,16.99)	6.35 (-2.93,19.77)	7.36 (-3.43,21.76)	8.12 (-3.83,22.68)	8.69 (-4.11,23.61)	9.14 (-4.41,24.15)	9.49 (-4.71,24.54)	9.77 (-4.87,24.74)	10.00 (-5.12,24.99)

(p) £85/dose; VaR; Some-unwilling; 1.5, 3 years

Uptake of 2nd dose (%) \ Initial vaccination uptake (%)	10	20	30	40	50	60	70	80	90	100
100	-0.08 (-1.97,3.39)	-0.11 (-3.90,6.68)	-0.11 (-5.81,9.45)	-0.13 (-7.66,11.91)	-0.22 (-9.44,13.34)	-0.44 (-11.21,13.94)	-0.82 (-12.87,13.73)	-1.40 (-14.53,13.05)	-2.19 (-16.10,12.08)	-3.20 (-17.53,10.81)
90	-0.13 (-1.96,3.23)	-0.21 (-3.89,6.35)	-0.27 (-5.79,9.07)	-0.35 (-7.66,11.50)	-0.49 (-9.43,13.05)	-0.75 (-11.15,13.54)	-1.17 (-12.85,13.08)	-1.78 (-14.55,12.60)	-2.59 (-16.09,11.64)	-3.61 (-17.57,10.36)
80	-0.19 (-1.96,3.12)	-0.33 (-3.89,6.12)	-0.46 (-5.77,8.59)	-0.60 (-7.59,10.99)	-0.80 (-9.41,12.36)	-1.11 (-11.19,12.90)	-1.57 (-12.83,12.44)	-2.21 (-14.51,11.84)	-3.04 (-16.06,11.33)	-4.06 (-17.56,9.69)
70	-0.26 (-1.95,2.89)	-0.47 (-3.86,5.69)	-0.67 (-5.73,8.25)	-0.88 (-7.57,10.24)	-1.15 (-9.38,11.54)	-1.51 (-11.16,12.00)	-2.02 (-12.86,11.84)	-2.69 (-14.54,11.24)	-3.53 (-16.09,10.58)	-4.56 (-17.68,9.09)
60	-0.33 (-1.96,2.61)	-0.63 (-3.86,5.19)	-0.91 (-5.72,7.71)	-1.20 (-7.58,9.49)	-1.54 (-9.40,10.74)	-1.97 (-11.12,10.93)	-2.52 (-12.84,11.13)	-3.22 (-14.55,10.55)	-4.08 (-16.23,9.96)	-5.10 (-17.79,8.57)
50	-0.42 (-1.95,2.40)	-0.81 (-3.86,4.71)	-1.18 (-5.74,6.88)	-1.56 (-7.55,8.92)	-1.98 (-9.33,9.48)	-2.47 (-11.12,10.04)	-3.07 (-12.87,10.10)	-3.80 (-14.54,9.68)	-4.67 (-16.14,8.99)	-5.70 (-17.78,7.44)
40	-0.52 (-1.93,2.17)	-1.01 (-3.82,4.15)	-1.49 (-5.67,6.13)	-1.96 (-7.50,7.52)	-2.47 (-9.31,8.47)	-3.03 (-11.09,9.18)	-3.68 (-12.85,9.07)	-4.44 (-14.51,8.36)	-5.32 (-16.17,7.78)	-6.34 (-17.87,6.41)
30	-0.63 (-1.93,1.74)	-1.24 (-3.83,3.47)	-1.82 (-5.70,4.95)	-2.40 (-7.53,6.28)	-3.00 (-9.34,7.35)	-3.64 (-11.11,7.89)	-4.34 (-12.85,8.07)	-5.13 (-14.55,7.24)	-6.02 (-16.21,6.38)	-7.03 (-17.79,5.31)
20	-0.75 (-1.90,1.43)	-1.48 (-3.77,2.83)	-2.18 (-5.60,4.20)	-2.87 (-7.40,5.08)	-3.57 (-9.19,5.74)	-4.29 (-10.95,6.28)	-5.05 (-12.69,6.44)	-5.88 (-14.41,6.32)	-6.78 (-16.09,5.47)	-7.76 (-17.76,4.73)
10	-0.88 (-1.91,1.14)	-1.73 (-3.81,2.25)	-2.56 (-5.69,3.33)	-3.37 (-7.53,4.23)	-4.17 (-9.35,5.03)	-4.98 (-11.17,5.40)	-5.81 (-12.92,5.45)	-6.68 (-14.67,5.11)	-7.59 (-16.42,5.02)	-8.57 (-18.14,4.28)
0	-1.01 (-1.95,0.84)	-1.99 (-3.88,1.68)	-2.94 (-5.78,2.45)	-3.87 (-7.67,3.18)	-4.79 (-9.54,3.91)	-5.71 (-11.39,4.54)	-6.62 (-13.24,4.58)	-7.55 (-15.05,4.78)	-8.49 (-16.84,4.61)	-9.47 (-18.60,4.09)

(q) £85/dose; VaR; Some-unwilling; 4 years

Uptake of 2nd dose (%) \ Initial vaccination uptake (%)	10	20	30	40	50	60	70	80	90	100
100	0.87 (-1.34,4.74)	1.82 (-2.66,9.17)	2.78 (-3.84,13.41)	3.72 (-4.98,16.72)	4.54 (-6.08,18.91)	5.15 (-7.08,19.71)	5.50 (-8.06,20.16)	5.55 (-8.99,20.22)	5.29 (-9.79,19.62)	4.74 (-10.60,18.38)
90	0.84 (-1.31,4.53)	1.74 (-2.56,8.93)	2.67 (-3.73,13.09)	3.56 (-4.88,16.52)	4.35 (-5.90,18.34)	4.95 (-6.95,19.43)	5.31 (-7.92,19.97)	5.38 (-8.79,20.15)	5.16 (-9.65,19.49)	4.65 (-10.49,18.40)
80	0.80 (-1.28,4.39)	1.66 (-2.51,8.65)	2.54 (-3.64,12.69)	3.40 (-4.71,15.93)	4.16 (-5.82,17.67)	4.75 (-6.83,19.15)	5.11 (-7.72,19.42)	5.21 (-8.62,19.97)	5.03 (-9.51,19.26)	4.58 (-10.24,18.41)
70	0.76 (-1.23,4.19)	1.57 (-2.42,8.17)	2.41 (-3.56,12.08)	3.23 (-4.61,15.34)	3.96 (-5.60,17.31)	4.54 (-6.59,18.60)	4.91 (-7.54,19.33)	5.04 (-8.44,19.59)	4.91 (-9.29,19.06)	4.52 (-10.03,18.57)
60	0.72 (-1.18,4.05)	1.48 (-2.31,7.95)	2.28 (-3.43,11.57)	3.05 (-4.49,14.52)	3.76 (-5.47,16.70)	4.33 (-6.41,18.18)	4.72 (-7.35,18.85)	4.88 (-8.17,19.17)	4.80 (-9.00,18.68)	4.47 (-9.81,18.07)
50	0.67 (-1.12,3.77)	1.39 (-2.21,7.45)	2.14 (-3.28,10.85)	2.88 (-4.27,13.79)	3.55 (-5.25,16.25)	4.12 (-6.21,17.71)	4.52 (-7.07,18.13)	4.72 (-7.92,18.64)	4.70 (-8.73,18.56)	4.45 (-9.35,17.99)
40	0.63 (-1.08,3.56)	1.30 (-2.12,7.08)	2.00 (-3.11,10.23)	2.70 (-4.09,13.08)	3.35 (-5.04,15.60)	3.91 (-5.96,17.44)	4.32 (-6.85,17.70)	4.56 (-7.68,18.00)	4.61 (-8.44,18.14)	4.44 (-9.18,18.14)
30	0.58 (-1.04,3.36)	1.21 (-2.06,6.69)	1.86 (-3.03,9.87)	2.52 (-3.98,12.37)	3.14 (-4.89,14.79)	3.69 (-5.77,16.55)	4.13 (-6.61,17.52)	4.41 (-7.42,17.90)	4.53 (-8.20,17.80)	4.46 (-8.88,17.76)
20	0.54 (-1.01,3.22)	1.12 (-1.98,6.51)	1.73 (-2.93,9.54)	2.35 (-3.84,12.28)	2.94 (-4.72,14.66)	3.49 (-5.59,16.05)	3.94 (-6.45,17.40)	4.27 (-7.29,18.20)	4.45 (-7.98,18.12)	4.47 (-8.74,17.91)
10	0.50 (-0.99,3.14)	1.04 (-1.96,6.25)	1.60 (-2.91,9.17)	2.18 (-3.83,12.01)	2.75 (-4.70,14.30)	3.28 (-5.59,16.27)	3.75 (-6.40,17.47)	4.12 (-7.27,18.35)	4.37 (-8.13,18.74)	4.49 (-8.93,18.51)
0	0.46 (-1.01,3.10)	0.96 (-2.00,6.10)	1.49 (-2.97,8.93)	2.03 (-3.94,11.85)	2.57 (-4.90,14.29)	3.09 (-5.84,16.29)	3.56 (-6.71,17.80)	3.96 (-7.63,18.70)	4.28 (-8.49,19.50)	4.48 (-9.32,19.58)

(r) £85/dose; VaR; Some-unwilling; 7.5 years

Uptake of 2nd dose (%) \ Initial vaccination uptake (%)	10	20	30	40	50	60	70	80	90	100
100	1.61 (-0.86,5.79)	3.29 (-1.65,11.26)	5.00 (-2.37,16.43)	6.63 (-3.00,20.00)	8.08 (-3.59,22.76)	9.23 (-4.08,23.91)	10.01 (-4.52,24.66)	10.41 (-4.86,24.84)	10.44 (-5.25,24.55)	10.12 (-5.51,23.17)
90	1.56 (-0.83,5.51)	3.19 (-1.61,10.87)	4.84 (-2.26,15.88)	6.43 (-2.85,19.52)	7.85 (-3.43,22.38)	9.01 (-3.85,23.79)	9.83 (-4.34,24.76)	10.28 (-4.64,24.63)	10.37 (-5.00,24.60)	10.14 (-5.40,23.31)
80	1.50 (-0.78,5.30)	3.08 (-1.53,10.52)	4.67 (-2.19,15.24)	6.22 (-2.76,18.91)	7.62 (-3.33,21.62)	8.78 (-3.86,23.28)	9.63 (-4.20,24.37)	10.14 (-4.47,24.45)	10.31 (-4.71,24.37)	10.16 (-4.95,23.60)
70	1.45 (-0.74,5.11)	2.96 (-1.44,10.04)	4.50 (-2.07,14.72)	6.00 (-2.68,18.24)	7.38 (-3.21,20.99)	8.55 (-3.72,22.99)	9.43 (-4.10,24.14)	10.00 (-4.31,24.06)	10.25 (-4.69,24.15)	10.19 (-4.98,23.60)
60	1.39 (-0.70,4.88)	2.85 (-1.36,9.58)	4.33 (-1.96,13.90)	5.79 (-2.54,17.49)	7.14 (-3.07,20.41)	8.31 (-3.53,22.56)	9.22 (-3.88,23.55)	9.85 (-4.12,24.08)	10.18 (-4.44,23.84)	10.22 (-4.67,23.95)
50	1.34 (-0.66,4.56)	2.74 (-1.28,9.14)	4.16 (-1.86,13.38)	5.57 (-2.39,17.08)	6.89 (-2.89,20.16)	8.06 (-3.32,21.75)	9.01 (-3.69,23.08)	9.70 (-4.00,23.59)	10.11 (-4.22,23.83)	10.25 (-4.44,23.83)
40	1.28 (-0.61,4.43)	2.62 (-1.19,8.77)	3.99 (-1.74,12.70)	5.34 (-2.25,16.04)	6.64 (-2.69,19.30)	7.80 (-3.10,21.31)	8.78 (-3.47,22.39)	9.52 (-3.78,23.34)	10.02 (-4.06,23.95)	10.27 (-4.30,23.93)
30	1.23 (-0.59,4.25)	2.50 (-1.16,8.50)	3.81 (-1.70,12.38)	5.12 (-2.17,15.77)	6.38 (-2.59,18.53)	7.53 (-2.96,20.95)	8.53 (-3.33,22.27)	9.33 (-3.65,23.04)	9.91 (-3.94,23.47)	10.26 (-4.21,23.69)
20	1.17 (-0.58,4.11)	2.39 (-1.13,8.20)	3.64 (-1.62,12.07)	4.89 (-2.12,15.62)	6.11 (-2.59,18.58)	7.25 (-3.01,20.56)	8.27 (-3.38,22.47)	9.12 (-3.72,23.15)	9.77 (-4.02,23.86)	10.23 (-4.28,23.79)
10	1.11 (-0.57,3.98)	2.27 (-1.12,7.93)	3.46 (-1.64,11.79)	4.66 (-2.14,15.24)	5.84 (-2.62,18.27)	6.96 (-3.07,20.68)	7.98 (-3.49,22.57)	8.87 (-3.89,23.51)	9.60 (-4.25,24.01)	10.15 (-4.57,24.46)
0	1.06 (-0.63,3.93)	2.16 (-1.24,7.74)	3.29 (-1.83,11.42)	4.44 (-2.36,15.06)	5.57 (-2.89,18.09)	6.66 (-3.44,20.57)	7.68 (-3.97,22.25)	8.59 (-4.39,23.72)	9.37 (-4.80,24.64)	10.00 (-5.12,24.99)

(s) £85/dose; VoD; All-willing; 1.5, 3 years

Uptake of 2nd dose (%) \ Initial vaccination uptake (%)	10	20	30	40	50	60	70	80	90	100
100	-1.66 (-3.76,1.23)	-2.78 (-6.51,2.20)	-3.60 (-8.67,2.94)	-4.21 (-10.36,3.51)	-4.70 (-11.78,3.95)	-5.10 (-12.96,4.33)	-5.44 (-13.97,4.65)	-5.73 (-14.76,4.92)	-5.99 (-15.50,5.15)	-6.23 (-16.23,5.35)
90	-1.59 (-3.56,1.09)	-2.70 (-6.20,1.96)	-3.52 (-8.32,2.68)	-4.16 (-10.03,3.24)	-4.66 (-11.43,3.70)	-5.08 (-12.63,4.07)	-5.44 (-13.61,4.38)	-5.75 (-14.52,4.64)	-6.03 (-15.27,4.87)	-6.28 (-15.99,5.06)
80	-1.52 (-3.35,0.95)	-2.61 (-5.91,1.71)	-3.44 (-7.98,2.34)	-4.09 (-9.69,2.93)	-4.62 (-11.05,3.43)	-5.06 (-12.28,3.79)	-5.44 (-13.30,4.09)	-5.77 (-14.22,4.35)	-6.06 (-15.10,4.55)	-6.33 (-15.76,4.68)
70	-1.45 (-3.14,0.91)	-2.52 (-5.65,1.65)	-3.35 (-7.66,2.25)	-4.01 (-9.30,2.71)	-4.56 (-10.69,3.08)	-5.02 (-11.85,3.38)	-5.42 (-12.93,3.64)	-5.77 (-13.89,3.90)	-6.08 (-14.73,4.08)	-6.37 (-15.45,4.26)
60	-1.38 (-2.95,0.83)	-2.42 (-5.31,1.50)	-3.25 (-7.27,2.04)	-3.92 (-8.90,2.47)	-4.48 (-10.25,2.84)	-4.97 (-11.44,3.14)	-5.39 (-12.48,3.38)	-5.76 (-13.49,3.59)	-6.09 (-14.38,3.79)	-6.40 (-15.22,3.95)
50	-1.30 (-2.76,0.70)	-2.32 (-5.01,1.28)	-3.14 (-6.86,1.77)	-3.82 (-8.48,2.19)	-4.40 (-9.80,2.54)	-4.90 (-11.02,2.84)	-5.34 (-12.13,3.09)	-5.74 (-13.19,3.31)	-6.09 (-14.03,3.50)	-6.42 (-14.80,3.67)
40	-1.22 (-2.53,0.55)	-2.20 (-4.64,1.07)	-3.01 (-6.45,1.52)	-3.70 (-8.02,1.89)	-4.29 (-9.43,2.20)	-4.81 (-10.60,2.47)	-5.28 (-11.72,2.71)	-5.69 (-12.71,2.91)	-6.07 (-13.58,3.08)	-6.42 (-14.38,3.24)
30	-1.14 (-2.30,0.41)	-2.08 (-4.30,0.78)	-2.88 (-6.07,1.11)	-3.57 (-7.59,1.40)	-4.17 (-8.93,1.67)	-4.71 (-10.11,1.91)	-5.19 (-11.22,2.12)	-5.63 (-12.23,2.29)	-6.03 (-13.22,2.44)	-6.40 (-14.07,2.60)
20	-1.05 (-2.11,0.39)	-1.95 (-3.99,0.75)	-2.73 (-5.61,1.08)	-3.41 (-7.04,1.38)	-4.02 (-8.35,1.65)	-4.57 (-9.63,1.87)	-5.07 (-10.82,2.04)	-5.53 (-11.78,2.23)	-5.96 (-12.65,2.40)	-6.35 (-13.55,2.56)
10	-0.97 (-1.91,0.42)	-1.81 (-3.64,0.81)	-2.57 (-5.22,1.16)	-3.24 (-6.62,1.48)	-3.85 (-7.94,1.75)	-4.41 (-9.16,2.00)	-4.93 (-10.31,2.24)	-5.41 (-11.36,2.42)	-5.85 (-12.36,2.57)	-6.27 (-13.30,2.71)
0	-0.88 (-1.72,0.42)	-1.67 (-3.32,0.78)	-2.38 (-4.81,1.11)	-3.04 (-6.21,1.40)	-3.65 (-7.52,1.65)	-4.22 (-8.76,1.88)	-4.74 (-9.95,2.08)	-5.24 (-11.03,2.27)	-5.70 (-12.04,2.45)	-6.14 (-13.04,2.62)

(t) £85/dose; VoD; All-willing; 4 years

Uptake of 2nd dose (%) \ Initial vaccination uptake (%)	10	20	30	40	50	60	70	80	90	100
100	-0.05 (-2.75,3.76)	0.09 (-4.65,6.50)	0.28 (-6.08,8.57)	0.49 (-7.16,10.13)	0.68 (-7.99,11.34)	0.86 (-8.74,12.22)	1.02 (-9.43,13.04)	1.16 (-9.99,13.79)	1.28 (-10.50,14.43)	1.38 (-10.85,14.94)
90	-0.03 (-2.58,3.49)	0.11 (-4.39,6.10)	0.31 (-5.76,8.11)	0.51 (-6.83,9.70)	0.70 (-7.67,10.80)	0.88 (-8.34,11.80)	1.03 (-9.01,12.69)	1.17 (-9.58,13.45)	1.29 (-10.06,14.11)	1.39 (-10.44,14.68)
80	0.00 (-2.40,3.34)	0.15 (-4.13,5.85)	0.34 (-5.43,7.77)	0.54 (-6.43,9.15)	0.72 (-7.28,10.45)	0.90 (-7.95,11.42)	1.05 (-8.60,12.32)	1.19 (-9.17,13.08)	1.30 (-9.60,13.75)	1.41 (-9.97,14.29)
70	0.03 (-2.23,3.20)	0.18 (-3.86,5.51)	0.37 (-5.09,7.31)	0.57 (-6.10,8.80)	0.75 (-6.96,10.02)	0.92 (-7.61,11.03)	1.08 (-8.15,11.91)	1.21 (-8.65,12.65)	1.33 (-9.09,13.29)	1.43 (-9.51,13.85)
60	0.06 (-2.05,3.03)	0.22 (-3.57,5.35)	0.41 (-4.79,7.14)	0.60 (-5.76,8.56)	0.79 (-6.60,9.72)	0.95 (-7.19,10.69)	1.11 (-7.70,11.56)	1.24 (-8.16,12.32)	1.36 (-8.65,12.95)	1.46 (-9.09,13.54)
50	0.09 (-1.88,2.88)	0.25 (-3.34,5.10)	0.45 (-4.45,6.86)	0.64 (-5.43,8.26)	0.83 (-6.20,9.39)	0.99 (-6.78,10.35)	1.14 (-7.34,11.25)	1.28 (-7.81,11.94)	1.39 (-8.21,12.51)	1.50 (-8.67,13.11)
40	0.12 (-1.73,2.64)	0.30 (-3.10,4.77)	0.49 (-4.15,6.47)	0.69 (-5.04,7.85)	0.87 (-5.78,8.99)	1.04 (-6.44,9.89)	1.19 (-6.99,10.70)	1.32 (-7.41,11.42)	1.44 (-7.86,12.04)	1.55 (-8.30,12.63)
30	0.15 (-1.59,2.51)	0.34 (-2.86,4.47)	0.54 (-3.87,6.05)	0.74 (-4.66,7.43)	0.93 (-5.38,8.56)	1.09 (-6.02,9.57)	1.24 (-6.57,10.38)	1.38 (-7.07,11.08)	1.50 (-7.51,11.76)	1.61 (-7.92,12.40)
20	0.18 (-1.46,2.52)	0.39 (-2.67,4.56)	0.60 (-3.62,6.22)	0.80 (-4.46,7.62)	0.99 (-5.17,8.81)	1.16 (-5.80,9.82)	1.31 (-6.34,10.69)	1.45 (-6.82,11.45)	1.57 (-7.28,12.14)	1.68 (-7.73,12.72)
10	0.22 (-1.35,2.51)	0.44 (-2.46,4.57)	0.66 (-3.41,6.30)	0.87 (-4.20,7.76)	1.06 (-4.94,8.99)	1.23 (-5.54,10.05)	1.39 (-6.06,10.93)	1.53 (-6.56,11.78)	1.65 (-7.04,12.50)	1.76 (-7.50,13.13)
0	0.25 (-1.24,2.49)	0.50 (-2.31,4.55)	0.73 (-3.25,6.28)	0.94 (-4.07,7.78)	1.14 (-4.78,9.07)	1.32 (-5.42,10.23)	1.47 (-6.03,11.23)	1.62 (-6.58,12.08)	1.75 (-7.07,12.87)	1.86 (-7.54,13.57)

(u) £85/dose; VoD; All-willing; 7.5 years

Uptake of 2nd dose (%) \ Initial vaccination uptake (%)	10	20	30	40	50	60	70	80	90	100
100	0.99 (-2.03,5.32)	1.94 (-3.40,9.04)	2.79 (-4.40,11.71)	3.54 (-5.01,13.73)	4.18 (-5.44,15.44)	4.74 (-5.89,16.63)	5.22 (-6.15,17.61)	5.65 (-6.43,18.49)	6.02 (-6.68,19.30)	6.35 (-6.91,20.00)
90	0.98 (-1.88,5.01)	1.92 (-3.13,8.65)	2.76 (-4.11,11.33)	3.49 (-4.72,13.44)	4.13 (-5.15,15.08)	4.69 (-5.50,16.39)	5.17 (-5.79,17.54)	5.60 (-6.06,18.49)	5.97 (-6.31,19.34)	6.30 (-6.60,20.07)
80	0.97 (-1.74,4.82)	1.90 (-2.97,8.22)	2.73 (-3.81,10.90)	3.45 (-4.39,13.03)	4.09 (-4.84,14.74)	4.64 (-5.20,16.07)	5.12 (-5.50,17.14)	5.54 (-5.79,18.03)	5.92 (-6.06,18.81)	6.24 (-6.25,19.51)
70	0.97 (-1.63,4.56)	1.88 (-2.72,7.92)	2.69 (-3.51,10.45)	3.41 (-4.11,12.49)	4.04 (-4.56,14.06)	4.59 (-4.91,15.50)	5.07 (-5.19,16.56)	5.49 (-5.43,17.48)	5.86 (-5.64,18.34)	6.19 (-5.82,19.12)
60	0.96 (-1.47,4.36)	1.86 (-2.50,7.64)	2.66 (-3.26,10.19)	3.37 (-3.82,12.08)	3.99 (-4.26,13.76)	4.53 (-4.58,15.14)	5.01 (-4.86,16.30)	5.43 (-5.11,17.24)	5.81 (-5.30,18.05)	6.14 (-5.47,18.77)
50	0.95 (-1.35,4.20)	1.84 (-2.33,7.39)	2.63 (-3.00,9.84)	3.33 (-3.53,11.82)	3.94 (-3.93,13.49)	4.48 (-4.24,14.82)	4.96 (-4.54,15.93)	5.38 (-4.77,16.95)	5.75 (-4.96,17.78)	6.08 (-5.12,18.49)
40	0.95 (-1.21,3.90)	1.82 (-2.09,6.87)	2.60 (-2.75,9.27)	3.29 (-3.25,11.20)	3.89 (-3.69,12.78)	4.43 (-4.04,14.19)	4.90 (-4.25,15.41)	5.32 (-4.53,16.46)	5.69 (-4.71,17.37)	6.02 (-4.90,18.15)
30	0.94 (-1.12,3.74)	1.80 (-1.95,6.68)	2.57 (-2.55,9.00)	3.25 (-3.02,10.88)	3.85 (-3.39,12.54)	4.37 (-3.70,13.90)	4.84 (-4.01,15.05)	5.26 (-4.25,16.11)	5.63 (-4.49,16.99)	5.96 (-4.71,17.78)
20	0.93 (-1.08,3.81)	1.78 (-1.86,6.81)	2.54 (-2.43,9.20)	3.21 (-2.88,11.11)	3.80 (-3.29,12.73)	4.32 (-3.64,14.15)	4.78 (-3.93,15.31)	5.20 (-4.12,16.31)	5.56 (-4.31,17.19)	5.89 (-4.57,17.95)
10	0.93 (-1.01,3.77)	1.77 (-1.79,6.72)	2.51 (-2.42,9.06)	3.17 (-2.88,11.03)	3.75 (-3.23,12.67)	4.26 (-3.57,14.07)	4.72 (-3.89,15.29)	5.13 (-4.14,16.28)	5.50 (-4.38,17.18)	5.83 (-4.62,18.05)
0	0.92 (-0.95,3.72)	1.75 (-1.71,6.73)	2.48 (-2.35,9.20)	3.13 (-2.90,11.24)	3.70 (-3.37,12.83)	4.21 (-3.73,14.16)	4.66 (-4.13,15.27)	5.07 (-4.47,16.23)	5.43 (-4.74,17.05)	5.75 (-5.02,17.93)

(v) £85/dose; VoD; Some-unwilling; 1.5, 3 years

Uptake of 2nd dose (%) \ Initial vaccination uptake (%)	10	20	30	40	50	60	70	80	90	100
100	-0.67 (-1.79,0.86)	-1.33 (-3.53,1.60)	-1.97 (-5.23,2.22)	-2.61 (-6.89,2.73)	-3.23 (-8.54,3.21)	-3.85 (-10.13,3.62)	-4.45 (-11.66,3.99)	-5.05 (-13.18,4.35)	-5.64 (-14.61,4.58)	-6.22 (-16.03,4.80)
90	-0.68 (-1.76,0.80)	-1.35 (-3.49,1.50)	-2.01 (-5.17,2.12)	-2.65 (-6.82,2.61)	-3.28 (-8.42,3.04)	-3.90 (-10.01,3.41)	-4.51 (-11.56,3.77)	-5.11 (-13.01,4.05)	-5.70 (-14.44,4.26)	-6.28 (-15.84,4.48)
80	-0.70 (-1.74,0.75)	-1.38 (-3.45,1.40)	-2.04 (-5.11,1.99)	-2.69 (-6.73,2.48)	-3.33 (-8.33,2.88)	-3.95 (-9.87,3.22)	-4.57 (-11.37,3.50)	-5.17 (-12.83,3.82)	-5.76 (-14.25,4.02)	-6.34 (-15.63,4.22)
70	-0.71 (-1.71,0.70)	-1.40 (-3.39,1.31)	-2.07 (-5.04,1.83)	-2.73 (-6.66,2.29)	-3.37 (-8.22,2.71)	-4.00 (-9.73,3.04)	-4.62 (-11.20,3.32)	-5.22 (-12.68,3.54)	-5.81 (-14.09,3.73)	-6.39 (-15.46,3.90)
60	-0.72 (-1.68,0.64)	-1.42 (-3.34,1.21)	-2.10 (-4.97,1.70)	-2.76 (-6.53,2.11)	-3.41 (-8.07,2.47)	-4.04 (-9.56,2.79)	-4.66 (-11.00,3.10)	-5.27 (-12.43,3.35)	-5.85 (-13.86,3.54)	-6.43 (-15.26,3.69)
50	-0.73 (-1.65,0.58)	-1.43 (-3.27,1.09)	-2.12 (-4.84,1.53)	-2.79 (-6.37,1.92)	-3.44 (-7.90,2.27)	-4.08 (-9.36,2.60)	-4.69 (-10.78,2.87)	-5.30 (-12.15,3.12)	-5.88 (-13.54,3.34)	-6.46 (-14.92,3.50)
40	-0.73 (-1.60,0.52)	-1.44 (-3.17,0.98)	-2.14 (-4.69,1.39)	-2.81 (-6.21,1.75)	-3.46 (-7.71,2.08)	-4.09 (-9.13,2.40)	-4.71 (-10.52,2.68)	-5.31 (-11.91,2.92)	-5.90 (-13.29,3.13)	-6.46 (-14.64,3.30)
30	-0.73 (-1.54,0.45)	-1.45 (-3.05,0.87)	-2.14 (-4.54,1.24)	-2.81 (-6.02,1.57)	-3.46 (-7.46,1.89)	-4.09 (-8.87,2.19)	-4.71 (-10.25,2.47)	-5.31 (-11.61,2.70)	-5.88 (-12.98,2.90)	-6.45 (-14.31,3.10)
20	-0.73 (-1.48,0.39)	-1.44 (-2.94,0.76)	-2.13 (-4.38,1.08)	-2.80 (-5.80,1.40)	-3.44 (-7.20,1.71)	-4.07 (-8.57,1.99)	-4.68 (-9.94,2.25)	-5.27 (-11.28,2.49)	-5.84 (-12.59,2.72)	-6.40 (-13.87,2.93)
10	-0.72 (-1.42,0.33)	-1.42 (-2.81,0.64)	-2.10 (-4.19,0.93)	-2.76 (-5.55,1.21)	-3.40 (-6.88,1.49)	-4.02 (-8.19,1.74)	-4.62 (-9.47,1.98)	-5.20 (-10.77,2.22)	-5.76 (-12.05,2.47)	-6.31 (-13.31,2.70)
0	-0.70 (-1.33,0.27)	-1.39 (-2.65,0.53)	-2.05 (-3.95,0.78)	-2.69 (-5.24,1.03)	-3.32 (-6.50,1.29)	-3.92 (-7.77,1.52)	-4.51 (-9.02,1.77)	-5.08 (-10.25,2.00)	-5.63 (-11.46,2.23)	-6.17 (-12.63,2.43)

(w) £85/dose; VoD; Some-unwilling; 4 years

Uptake of 2nd dose (%)	10	20	30	40	50	60	70	80	90	100
100	0.15 (-1.29,2.14)	0.31 (-2.55,4.12)	0.47 (-3.76,5.94)	0.63 (-4.90,7.55)	0.79 (-5.98,9.16)	0.93 (-6.99,10.53)	1.06 (-7.94,11.87)	1.19 (-8.83,13.07)	1.30 (-9.67,14.02)	1.40 (-10.53,14.77)
90	0.14 (-1.27,2.09)	0.30 (-2.49,4.02)	0.45 (-3.66,5.81)	0.61 (-4.77,7.42)	0.76 (-5.82,8.98)	0.90 (-6.80,10.34)	1.04 (-7.73,11.63)	1.16 (-8.60,12.84)	1.28 (-9.47,13.85)	1.39 (-10.36,14.69)
80	0.14 (-1.23,2.02)	0.28 (-2.41,3.93)	0.43 (-3.55,5.67)	0.59 (-4.63,7.25)	0.74 (-5.65,8.76)	0.88 (-6.61,10.14)	1.02 (-7.51,11.37)	1.15 (-8.38,12.59)	1.27 (-9.30,13.70)	1.38 (-10.12,14.60)
70	0.13 (-1.19,1.97)	0.27 (-2.33,3.85)	0.42 (-3.43,5.53)	0.57 (-4.48,7.03)	0.72 (-5.46,8.53)	0.87 (-6.39,9.87)	1.01 (-7.27,11.12)	1.14 (-8.18,12.28)	1.27 (-9.08,13.43)	1.39 (-9.85,14.46)
60	0.13 (-1.14,1.92)	0.27 (-2.24,3.75)	0.41 (-3.30,5.38)	0.56 (-4.31,6.90)	0.71 (-5.25,8.28)	0.86 (-6.15,9.69)	1.00 (-7.05,10.85)	1.14 (-7.96,12.04)	1.28 (-8.77,13.10)	1.40 (-9.54,14.17)
50	0.13 (-1.09,1.88)	0.26 (-2.14,3.64)	0.41 (-3.15,5.24)	0.56 (-4.12,6.75)	0.71 (-5.03,8.06)	0.86 (-5.91,9.43)	1.01 (-6.83,10.69)	1.15 (-7.65,11.78)	1.30 (-8.43,12.84)	1.43 (-9.19,13.81)
40	0.13 (-1.03,1.82)	0.26 (-2.03,3.56)	0.41 (-3.00,5.18)	0.56 (-3.92,6.62)	0.72 (-4.79,7.96)	0.87 (-5.69,9.13)	1.03 (-6.51,10.45)	1.18 (-7.31,11.58)	1.33 (-8.07,12.61)	1.47 (-8.82,13.55)
30	0.13 (-0.98,1.77)	0.27 (-1.92,3.46)	0.42 (-2.83,5.05)	0.57 (-3.70,6.54)	0.73 (-4.56,7.85)	0.90 (-5.38,9.03)	1.06 (-6.17,10.12)	1.22 (-6.94,11.34)	1.38 (-7.70,12.38)	1.53 (-8.45,13.33)
20	0.14 (-0.91,1.72)	0.28 (-1.79,3.36)	0.44 (-2.64,4.92)	0.60 (-3.47,6.38)	0.76 (-4.27,7.74)	0.93 (-5.05,8.89)	1.10 (-5.82,9.98)	1.27 (-6.57,11.10)	1.44 (-7.31,12.12)	1.61 (-8.01,13.10)
10	0.14 (-0.84,1.67)	0.30 (-1.66,3.25)	0.46 (-2.45,4.77)	0.63 (-3.22,6.21)	0.81 (-3.98,7.57)	0.99 (-4.72,8.80)	1.17 (-5.45,9.85)	1.35 (-6.16,10.93)	1.53 (-6.86,11.93)	1.71 (-7.52,12.85)
0	0.16 (-0.77,1.60)	0.32 (-1.51,3.16)	0.50 (-2.24,4.63)	0.68 (-2.96,6.04)	0.87 (-3.66,7.38)	1.06 (-4.35,8.64)	1.25 (-5.03,9.79)	1.45 (-5.69,10.89)	1.64 (-6.33,11.91)	1.84 (-6.96,12.85)

Initial vaccination uptake (%)

(x) £85/dose; VoD; Some-unwilling; 7.5 years

Uptake of 2nd dose (%)	10	20	30	40	50	60	70	80	90	100
100	0.73 (-0.93,3.05)	1.47 (-1.79,5.90)	2.19 (-2.59,8.52)	2.89 (-3.33,10.96)	3.57 (-4.01,13.14)	4.21 (-4.64,15.22)	4.81 (-5.25,16.91)	5.37 (-5.80,18.23)	5.89 (-6.25,19.47)	6.38 (-6.67,20.57)
90	0.72 (-0.89,2.97)	1.43 (-1.72,5.76)	2.14 (-2.49,8.34)	2.83 (-3.21,10.67)	3.49 (-3.87,12.86)	4.12 (-4.49,14.90)	4.72 (-5.12,16.71)	5.28 (-5.63,17.94)	5.81 (-6.08,19.30)	6.30 (-6.52,20.44)
80	0.70 (-0.85,2.90)	1.40 (-1.65,5.60)	2.09 (-2.39,8.11)	2.76 (-3.08,10.41)	3.41 (-3.72,12.52)	4.04 (-4.38,14.51)	4.63 (-4.95,16.45)	5.20 (-5.45,17.79)	5.73 (-5.91,18.99)	6.22 (-6.35,20.23)
70	0.68 (-0.81,2.83)	1.36 (-1.57,5.45)	2.04 (-2.28,7.86)	2.70 (-2.95,10.20)	3.34 (-3.61,12.20)	3.96 (-4.23,14.20)	4.55 (-4.76,16.03)	5.11 (-5.25,17.62)	5.64 (-5.72,18.71)	6.15 (-6.17,19.86)
60	0.66 (-0.77,2.76)	1.33 (-1.49,5.29)	1.99 (-2.17,7.61)	2.64 (-2.83,9.97)	3.27 (-3.48,11.96)	3.88 (-4.05,13.89)	4.47 (-4.56,15.59)	5.03 (-5.05,17.34)	5.57 (-5.52,18.43)	6.07 (-5.99,19.57)
50	0.65 (-0.72,2.67)	1.30 (-1.41,5.15)	1.94 (-2.07,7.43)	2.58 (-2.71,9.67)	3.21 (-3.31,11.72)	3.81 (-3.84,13.52)	4.40 (-4.35,15.26)	4.96 (-4.84,16.86)	5.49 (-5.32,18.24)	6.00 (-5.78,19.31)
40	0.63 (-0.68,2.61)	1.27 (-1.33,5.06)	1.90 (-1.97,7.28)	2.53 (-2.57,9.33)	3.14 (-3.12,11.42)	3.74 (-3.64,13.22)	4.33 (-4.13,14.93)	4.89 (-4.62,16.44)	5.42 (-5.09,17.97)	5.94 (-5.54,19.02)
30	0.62 (-0.64,2.53)	1.24 (-1.25,4.94)	1.86 (-1.85,7.13)	2.48 (-2.39,9.13)	3.09 (-2.92,11.08)	3.68 (-3.42,12.92)	4.26 (-3.91,14.56)	4.82 (-4.39,16.10)	5.36 (-4.84,17.48)	5.88 (-5.24,18.82)
20	0.61 (-0.59,2.44)	1.22 (-1.16,4.79)	1.83 (-1.70,6.97)	2.44 (-2.22,8.86)	3.04 (-2.72,10.77)	3.62 (-3.19,12.56)	4.20 (-3.67,14.22)	4.76 (-4.12,15.74)	5.30 (-4.55,17.14)	5.82 (-4.89,18.40)
10	0.59 (-0.54,2.36)	1.19 (-1.06,4.61)	1.80 (-1.56,6.78)	2.40 (-2.04,8.69)	2.99 (-2.50,10.51)	3.57 (-2.97,12.23)	4.15 (-3.41,13.91)	4.70 (-3.83,15.36)	5.25 (-4.19,16.77)	5.77 (-4.50,18.07)
0	0.59 (-0.49,2.28)	1.18 (-0.96,4.47)	1.77 (-1.41,6.52)	2.36 (-1.85,8.51)	2.95 (-2.28,10.33)	3.53 (-2.71,12.05)	4.10 (-3.12,13.63)	4.66 (-3.47,15.09)	5.20 (-3.79,16.40)	5.73 (-4.08,17.86)

Initial vaccination uptake (%)

References

[1] Whittles LK, Didelot X, White PJ. Public health impact and cost-effectiveness of gonorrhoea vaccination: an integrated transmission-dynamic health-economic modelling analysis. Lancet Infectious Diseases. 2022;22(7):1030-41.

[2] Garnett GP, Mertz KJ, Finelli L, Levine WC, St Louis ME. The transmission dynamics of gonorrhoea: modelling the reported behaviour of infected patients from Newark, New Jersey. Philosophical Transactions of the Royal Society of London Series B: Biological Sciences. 1999;354:787-97.

[3] McLean AR, Blower SM. Imperfect vaccines and herd immunity to HIV. Proceedings of the Royal Society B: Biological Sciences. 1993;253:9-13.

[4] UK Health Security Agency. Sexually transmitted infections (STIs): annual data tables;. Available from: https://www.gov.uk/government/statistics/sexually-transmitted-infections-stis-annual-data-tables.

[5] UK Health Security Agency. Gonococcal resistance to antimicrobials surveillance programme report;. Available from: https://www.gov.uk/government/publications/gonococcal-resistance-to-antimicrobials-surveillance-programme-grasp-report.

[6] Whittles LK, White PJ, Didelot X. Assessment of the potential of vaccination to combat antibiotic resistance in gonorrhea: a modeling analysis to determine Preferred Product Characteristics. Clinical Infectious Diseases. 2020;71(8):1912-9.

[7] Public Health England. HPV vaccination uptake in gay, bisexual and other men who have sex with men (MSM): national programme, 2019 annual report. London; 2019. Available from: https://assets.publishing.service.gov.uk/government/uploads/system/uploads/attachment_data/file/968944/MSM_uptake_annual_report_2019.pdf.

[8] Public Health England. Meningococcal B vaccination. Information for healthcare practitioners. London: Public Health England; 2021. Available from: https://assets.publishing.service.gov.uk/government/uploads/system/uploads/attachment_data/file/998409/Meningococcal_B_vaccination_information_for_healthcare_practitioners_July21.pdf.

[9] Nolan T, Santolaya ME, de Looze F, et al. Antibody persistence and booster response in adolescents and young adults 4 and 7.5 years after immunization with 4CMenB vaccine. Vaccine. 2019;37(9):1209-18.

[10] Office for National Statistics. United Kingdom population mid-year estimate; 2016. Available from: www.ons.gov.uk/peoplepopulationandcommunity/populationandmigration/populationestimates/timeseries/ukpop/pop.

[11] Johnson A, University College London, Centre for Sexual Health and HIV Research. National Survey of Sexual Attitudes and Lifestyles, 2010-2012. [data collection]. UK Data Service. SN: 7799; 2021. Available from: http://doi.org/10.5255/UKDA-SN-7799-2.

[12] Abara WE, Bernstein KT, Lewis FMT, et al. Effectiveness of a serogroup B outer membrane vesicle meningococcal vaccine against gonorrhoea: a retrospective observational study. Lancet Infectious Diseases. 2022;22(7):1021-9.

[13] Fitzjohn RG, et al. odin;. Available from: https://github.com/mrc-ide/odin.

[14] Jones KC, Burns A. Unit Costs of Health and Social Care 2021. Personal Social Services Research Unit, University of Kent; 2021.

[15] Adams EJ, Ehrlich A, Turner KME, et al. Mapping patient pathways and estimating resource use for point of care versus standard testing and treatment of chlamydia and gonorrhoea in genitourinary medicine clinics in the UK. BMJ Open. 2014;4(e005322).

[16] National Institute for Health and Care Excellence (NICE). Developing NICE guidelines: the manual; 2014. Available from: https://www.nice.org.uk/Media/Default/About/what-we-do/our-programmes/developing-NICE-guidelines-the-manual.pdf.

[17] Reitsema M, Van Hoek AJ, Van Der Loeff MS, et al. Preexposure prophylaxis for men who have sex with men in the Netherlands: Impact on HIV and Neisseria gonorrhoeae transmission and cost-effectiveness. Aids. 2020;34(4):621-30.

[18] Institute of Medicine. Vaccines for the 21st Century: A Tool for Decision Making. Washington, DC: The National Academies Press; 2000. Available from: https://doi.org/10.17226/5501.

[19] Joint Committee on Vaccination and Immunisation. Code of Practice. Department of Health; 2013. Available from: www.gov.uk/government/uploads/system/uploads/attachment_data/file/224864/JCVI_Code_of_Practice_revision_2013_-_final.pdf.

[20] British National Formulary. Meningococcal group B Vaccine (rDNA, component, absorbed);. Available from: https://bnf.nice.org.uk/medicinal-forms/meningococcal-group-b-vaccine-rdna-component-adsorbed.html.

[21] NHS England. Human Papilloma Virus (HPV) immunisation programme for men that have sex with men (MSM) 2018/19; 2019. Available from: https://www.england.nhs.uk/wp-content/uploads/2019/01/hpv-msm-faqs-for-hcp.pdf.

[22] Christensen H, Hickman M, Edmunds WJ, Trotter CL. Introducing vaccination against serogroup B meningococcal disease: An economic and mathematical modelling study of potential impact. Vaccine. 2013;31(23):2638-46.
